# Grafting Electron‐Accepting Fragments on [4]cyclo‐2,7‐carbazole Scaffold: Tuning the Structural and Electronic Properties of Nanohoops

**DOI:** 10.1002/advs.202309115

**Published:** 2024-01-22

**Authors:** Clément Brouillac, Nemo McIntosh, Benoît Heinrich, Olivier Jeannin, Olivier De Sagazan, Nathalie Coulon, Joëlle Rault‐Berthelot, Jérôme Cornil, Emmanuel Jacques, Cassandre Quinton, Cyril Poriel

**Affiliations:** ^1^ Univ Rennes CNRS ISCR‐UMR 6226 Rennes F‐35000 France; ^2^ Laboratory for Chemistry of Novel Materials University of Mons Mons B‐7000 Belgium; ^3^ Institut de Physique et Chimie des Matériaux de Strasbourg (IPCMS) UMR 7504 CNRS‐Université de Strasbourg 23 rue du Loess, BP 43, Cedex 2 Strasbourg 67034 France; ^4^ Univ Rennes CNRS IETR‐UMR 6164 Rennes F‐35000 France

**Keywords:** bridged cyclo‐oligophenylenes, charge transport, nanohoops, organic electronics, organic semiconductors

## Abstract

Since the first applications of nanohoops in organic electronics appear promising, the time has come to go deeper into their rational design in order to reach high‐efficiency materials. To do so, systematic studies dealing with the incorporation of electron‐rich and/or electron‐poor functional units on nanohoops have to be performed. Herein, the synthesis, the electrochemical, photophysical, thermal, and structural properties of two [4]cyclo‐2,7‐carbazoles, **[4]C‐Py‐Cbz**, and **[4]C‐Pm‐Cbz**, possessing electron‐withdrawing units on their nitrogen atoms (pyridine or pyrimidine) are reported. The synthesis of these nanohoops is first optimized and a high yield above 50% is reached. Through a structure‐properties relationship study, it is shown that the substituent has a significant impact on some physicochemical properties (eg HOMO/LUMO levels) while others are kept unchanged (eg fluorescence). Incorporation in electronic devices shows that the most electrically efficient Organic Field‐Effect transistors are obtained with **[4]C‐Py‐Cbz** although this compound does not present the best‐organized semiconductor layer. These experimental data are finally confronted with the electronic couplings between the nanohoops determined at the DFT level and have highlighted the origin in the difference of charge transport properties. **[4]C‐Py‐Cbz** has the advantage of a more 2D‐like transport character than **[4]C‐Pm‐Cbz**, which alleviates the impact of defects and structural organization.

## Graphical abstract

The synthesis, the electrochemical, photophysical, thermal, and structural properties of two [4]cyclo‐2,7‐carbazoles, [4]C‐Py‐Cbz and [4]C‐Pm‐Cbz, possessing electron‐withdrawing units on their nitrogen atoms (i.e., pyridine and pyrimidine) are reported. Incorporation in electronic devices shows that the most electrically efficient OFETs are obtained with [4]C‐Py‐Cbz although this compound does not present the best‐organized semiconductor layer.

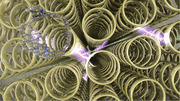

## 1 Introduction

Due to their unique architecture, nanohoops–fully conjugated macrocycles possessing radially directed molecular orbitals–display singular properties in comparison with their acyclic analogs.^[^ ^1^, ^2^, ^3^, ^4^, ^5^, ^6^, ^7^, ^8^, ^9^, ^10^, ^11^, ^12^, ^13^, ^14^, ^15^, ^16^, ^17^, ^18^, ^19^, ^20^, ^21^, ^22^, ^23^ ^]^ For example, due to the strain and the resulting geometry, the cyclo‐*para*‐phenylenes (CPPs), the flagship family of nanohoops, display shorter HOMO‐LUMO gap than their linear oligo‐*para*‐phenylenes counterparts. The modulation of the energy of the frontiers orbitals is at the heart of the molecular design of organic semiconductors (OSC) and at the origin of the development of organic electronics. Very recently, the first applications of nanohoops in electronic devices have shown the potential of this emerging generation of OSCs.^[^ ^1^, ^2^, ^24^ ^]^ Indeed, when used as a host in red phosphorescent OLEDs, a nanohoop is significantly more efficient than its acyclic analog, providing a high potential for the future of nanohoops.^[^ ^1^ ^]^ With more accurate molecular designs, there is no doubt that the performance of nanohoops as active layers in organic electronics will rapidly increase. Studying the evolution of the electronic and structural properties of nanohoops as a function of the substituent grafted to the nanohoop backbone is, therefore, an interesting direction to design high‐efficiency organic materials. One classical way to shorten the HOMO‐LUMO gap of an OSC consists of introducing some electron‐withdrawing and ‐donating fragments.^[^ ^25^, ^26^, ^27^, ^28^ ^]^ Thus, the synthesis of donor‐acceptor nanohoops could be a very efficient way to reach OSCs with a very short HOMO‐LUMO gap. Whereas CPPs incorporating electron‐rich and/or electron‐poor functional units in their backbone start to be synthesized,^[^ ^2^, ^4^, ^29^, ^30^, ^31^, ^32^, ^33^, ^34^, ^35^, ^36^, ^37^, ^38^, ^39^, ^40^, ^41^, ^42^, ^43^, ^44^, ^45^, ^46^, ^47^, ^48^, ^49^, ^50^, ^51^, ^52^ ^]^ electron‐rich nanohoops substituted by electron‐poor fragments in the periphery have never been reported. Since the interest in donor‐acceptor architectures in organic electronics is no longer to be demonstrated, designing donor‐acceptor nanohoops represents a key challenge for their future applications. In the present work, two well‐known accepting units, i.e., 2‐pyridine in **[4]C‐Py‐Cbz** and 2,5‐pyrimidine in **[4]C‐Pm‐Cbz** have been grafted on the nitrogen atoms of [4]cyclo‐2,7‐carbazoles. The [4]cyclo‐2,7‐carbazole platform has been investigated in light of recent advances in the field of organic electronics.^[^ ^1^, ^24^ ^]^ Note that a phenylene unit (**[4]C‐Ph‐Cbz**) was also introduced as model compound. This compound was previously synthesized by Yamago and co‐workers for the purpose of an NMR study, which aimed to investigate the inner regions of nanohoops.^[^ ^53^ ^]^ In the present work, based on a dual experimental and theoretical approach, we report the electrochemical, photophysical, thermal, and structural properties of a series of nanohoops, which show the impact of the electron‐withdrawing effect of nitrogen‐based heterocycles. Since the synthesis remains a central concern in the field of nanohoops, we present an optimized synthetic approach of [4]cyclo‐2,7‐carbazoles substituted with *N*‐based heterocycles (with a high yield higher than 50%). Finally, charge transport studies have been performed in field‐effect transistors and analyzed based on the electronic couplings between the nanohoops determined at the density functional theory (DFT) level in the single crystal structures. We particularly show that the most electrically efficient organic field effect transistor (OFET), obtained with **[4]C‐Py‐Cbz**, does not present the best‐organized semiconductor layer. Electronic couplings between the nanohoops have revealed the origin of the different charge transport properties. Indeed, **[4]C‐Py‐Cbz** has the advantage of a more 2D‐like transport character than **[4]C‐Pm‐Cbz**, which alleviates the impact of defects and structural organization.

### 1.1 Synthesis

**Scheme 1:**
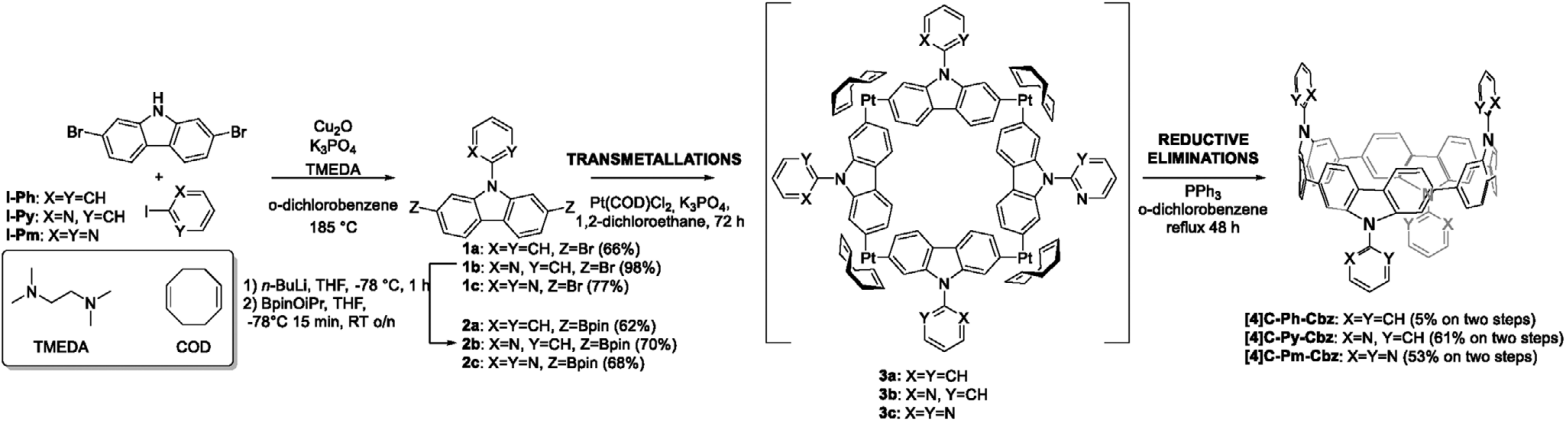
Synthesis of the targeted nanohoops **[4]C‐Ph‐Cbz**, **[4]C‐Py‐Cbz**, and **[4]C‐Pm‐Cbz**.

The synthetic pathway reported in **Scheme** **1** is inspired by the Pt‐mediated cyclization of Yamago,^[^ ^54^ ^]^ the boryl‐route modification of Isobe,^[^ ^55^ ^]^ and the optimizations performed by our group over the years.^[^ ^24^ ^]^ The brominated precursors **1a‐c** were obtained in good yields thanks to a classical Ullman reaction between 2,7‐dibromocarbazole and adequate iodinated compounds. The diboronic esters **2a‐c** were synthesized through a classical halogen‐lithium exchange followed by a borylation. The intermediates **3a‐c** were then formed (not isolated) by stirring the dipinacol compounds **2a‐c** with Pt(cod)Cl_2_ and CsF in refluxing 1,2‐dichloroethane for 24 h (see Table S1 in Supporting Information). These conditions have been reported as very efficient for the synthesis of cyclocarbazoles. Then, the dried crude mixtures containing **3a‐c** were treated with triphenylphosphine in refluxing *o*‐dichlorobenzene to give **[4]C‐Ph‐Cbz, [4]C‐Py‐Cbz** and **[4]C‐Pm‐Cbz** with 5, 33 and 30% yield on two steps, respectively (Table S1, in Supporting Information). However, we were surprised that these yields were lower than those reported for *N*‐alkyl analogs,^[^ ^24^ ^]^ highlighting the significant effect of the pending substituent on the yield of the reaction. Optimizations of the transmetallation reaction conditions were then performed on **2b** by modifying the reaction time (24–72 h) and the base (K_3_PO_4_ and CsF) and keeping the reductive eliminations unchanged. According to this study (see Table S1, Supporting Information), increasing the reaction time of the transmetallations to 72 h in the presence of CsF does not improve (nor damage) the reaction (yield of 28 % on two steps). By using K_3_PO_4_ instead of CsF and keeping the reaction time of 24 h, the yield becomes really low (3% on two steps). Finally, the use of K_3_PO_4_ and the increase of the reaction time to 48 and 72 h significantly improved the reaction yield to 61%. These optimal conditions (K_3_PO_4_, 72 h) were also successfully applied to **2c**, leading to **[4]C‐Pm‐Cbz** with a high yield of 53%. However, in the case of 2a, these conditions did not improve the yield of **[4]C‐Ph‐Cbz** (5%) surely due to insolubility issues. Thus, the optimal synthesis conditions of [4]cyclo‐2,7‐carbazoles (and nanohoops in general) appear to be strongly dependent on the diboronic precursors (and particularly the resulting solubility of the corresponding tetraplatinum intermediates) and a case‐by‐case analysis is always needed. This is the reason why large series of nanohoops are still very difficult to prepare via this approach. In the present case, we managed to reach a high yield of over 50% for both **[4]C‐Py‐Cbz** and **[4]C‐Pm‐Cbz**.

**Figure 1:**
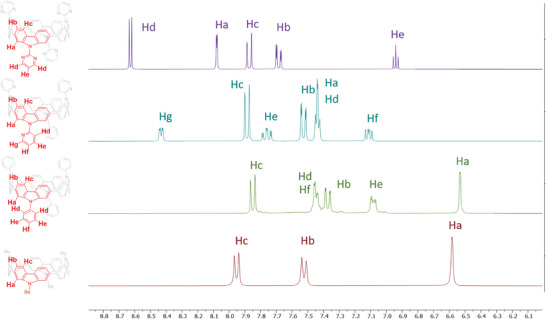
^1^H‐NMR spectrum of **[4]C‐Pm‐Cbz** (purple lines), **[4]C‐Py‐Cbz** (blue lines), **[4]C‐Ph‐Cbz** (green lines), and **[4]C‐Bu‐Cbz** (red lines) in CD_2_Cl_2_.

Pyridine and pyrimidine fragments are strong electron‐withdrawing units and their electronic effect on the carbazole nanohoop backbone can be visualized by ^1^H NMR spectroscopy. The strength of the electron‐withdrawing character of the pyridine and pyrimidine fragments can be directly correlated with the resulting shielding/deshielding effects of the hydrogen atoms of the nanohoop core. The ^1^H‐NMR spectrum of **[4]C‐Pm‐Cbz**, **[4]C‐Py‐Cbz**, **[4]C‐Ph‐Cbz** in CD_2_Cl_2_ are represented in **Figure** **1**. For comparison purposes, the ^1^H NMR spectrum of **[4]C‐Bu‐Cbz**, previously reported in the literature,^[^ ^24^ ^]^ is also presented. The protons of each nanohoop were assigned with 2D NMR experiments (COSY, HMBC, HSQC, see SI). First, it is important to mention that, for both **[4]C‐Ph‐Cbz** and **[4]C‐Bu‐Cbz**, no significant difference is observed neither for Ha (ca 6.5 ppm), Hb (ca 7.4 ppm), nor Hc (ca 7.9 ppm), showing that a phenyl ring and a butyl chain have a similar impact on the surrounding nanohoop hydrogen atoms. When pyridine and pyrimidine are grafted on the nitrogen, a different behavior is detected. Whereas the Hb and Hc signals are not strongly modified, the impact on Ha, which is the closest to the nitrogen bridge, is impressive. For **[4]C‐Py‐Cbz**, Ha (7.5 ppm) is significantly deshielded by 1 ppm compared to its analog in **[4]C‐Ph‐Cbz** and **[4]C‐Bu‐Cbz** (ca 6.5 ppm). Switching from a pyridine to a pyrimidine unit leads to a strong deshielding effect for the Ha resonance recorded at 8.1 ppm in **[4]C‐Pm‐Cbz**. This clearly translates the stronger electron‐withdrawing character of the pyrimidine unit compared to that of pyridine, which in turn should lead to different electronic properties (see below). Modifying the nature of the bridge is hence an interesting strategy to tune the electronic properties of a cyclocarbazole.

### 1.2 Structural Properties

**Figure 2:**
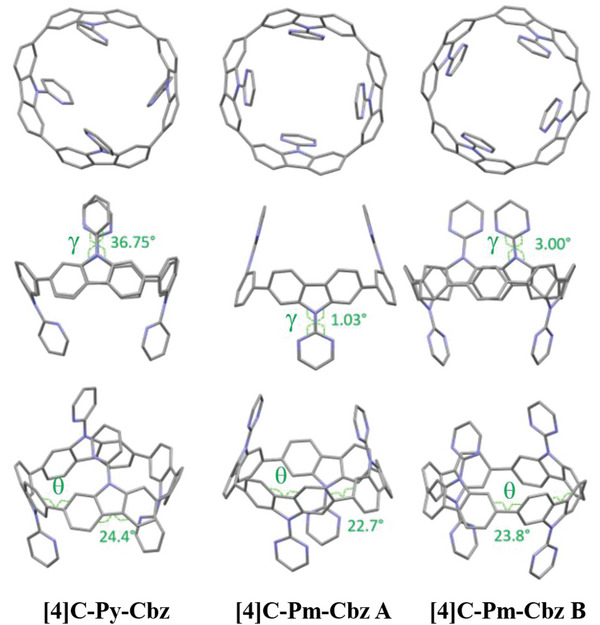
Different views of single‐crystal structures of **[4]C‐Py‐Cbz** (Left) and **[4]C‐Pm‐Cbz** (Molecule A, middle and Molecule B, right). For the sake of clarity, hydrogens and solvents are not shown.

Molecular structures of **[4]C‐Py‐Cbz** (CCDC n° 2 265 379) and **[4]C‐Pm‐Cbz** (CCDC n°2 265 380) were fully revealed by X‐ray diffraction on single crystals (**Figure** **2**). We note that **[4]C‐Py‐Cbz** crystallizes with molecules of dichloromethane localized inside the nanohoop whereas **[4]C‐Pm‐Cbz** crystallizes with molecules of dichloromethane localized outside the nanohoop. For both molecules, and in agreement with what is usually observed for nanohoops with four carbazole units,^[^ ^24^, ^56^ ^]^ an αβαβ conformation is observed. They form a slightly distorted ellipsoidal nanohoop with an averaged axis of 10.9 Å (minimal/maximal C‐C axis of 10.61/11.16 Å for **[4]C‐Py‐Cbz** and 10.65/11.12 Å for **[4]C‐Pm‐Cbz**). These data are similar to those reported for the alkyl‐substituted counterparts.^[^ ^24^ ^]^

**Figure 3:**
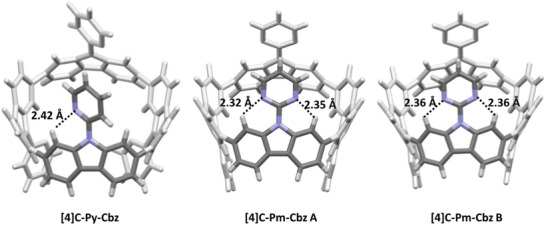
Intramolecular N‐H interactions for **[4]C‐Py‐Cbz** and **[4]C‐Pm‐Cbz**.

The deformations of the phenylenes and their relative arrangement are at the origin of the specific properties of nanohoops.^[^ ^3^, ^24^, ^52^, ^57^, ^58^, ^59^, ^60^ ^]^ Two other structural parameters are usually considered to describe a nanohoop: the displacement angles ω (bending of the phenylene units) and the torsion angles θ (torsion between neighboring phenylene units). These two structural parameters are of key importance to understanding the evolution of the HOMO and LUMO energy levels. The averaged displacement angles are similar for both nanohoops (ω of 6.8° for **[4]C‐Py‐Cbz** vs 6.7 and 7.0° for the two molecules of **[4]C‐Pm‐Cbz**) and slightly lower than that of [4]cyclo‐2,7‐carbazoles bearing alkyl chains (ω = 7.0–7.2°).^[^ ^24^ ^]^ The averaged torsion angle of **[4]C‐Py‐Cbz** (θ= 24.4°) is slightly higher than that of **[4]C‐Pm‐Cbz** (θ of 22.7 and 23.8°), both of them being higher than in *N*‐alkylated cyclocarbazoles (θ = 19.0–20.7°).^[^ ^24^ ^]^ This shows that the substituent borne by the nitrogen atom of the carbazoles has a non‐negligible impact on the structural characteristics of the nanohoops core. Furthermore, the pyrimidine substituent is in the plane of the carbazole (averaged dihedral angle between the carbazole and the substituent γ of 1.03 and 3.00° for **[4]C‐Pm‐Cbz**) whereas the pyridine substituent is twisted (γ of 36.75° for **[4]C‐Py‐Cbz**). In the case of **[4]C‐Pm‐Cbz**, the low torsion angle between carbazole and pyrimidine is favored by the formation of two hydrogen bonds (d_N‐H_<2.4 Å), this distance being significantly shorter than the sum of the van der Walls radii of nitrogen and hydrogen (2.75 Å, r_vdw_H /r_vdw_N = 1.20/1.55 Å), **Figure** **3**, middle and right.^[^ ^61^ ^]^ These interactions lock the pyrimidine ring in an almost planar conformation for both molecule A and molecule B. Oppositely, in the case of **[4]C‐Py‐Cbz**, only one short N‐H distance is measured, d_N‐H_ = 2.42 Å, (Figure 3, left). Thus, the steric repulsion between the hydrogen atoms Ha and Hd prevents the planarization of the pyridine. The more efficient planarization of the carbazole with respect to the substituent in the case of **[4]C‐Pm‐Cbz** compared to **[4]C‐Py‐Cbz** will induce a better conjugation between these two fragments and hence a stronger influence of pyrimidine compared to pyridine on the electronic properties of the cyclocarbazole, as detected by ^1^H NMR. Moreover, the number of nitrogen atoms of the heterocycle drives the carbazole/heterocycle dihedral angle γ and therefore influences the intermolecular packing as detailed below. Such a type of structural characteristic has already been observed in literature with the building units *N*‐pyridine and *N*‐pyrimidine carbazoles.^[^ ^62^ ^]^ It is interesting to note that the dihedral angles γ were higher compared to those measured in the present work (γ = 47/57° for *N*‐pyridine‐carbazole versus 37° for **[4]C‐Py‐Cbz**, 6° for *N*‐pyrimidine‐carbazole versus 1–3° for **[4]C‐Pm‐Cbz**), highlighting that the curvature in nanohoops significantly decreases the dihedral angles γ. This is corroborated by the different N‐H measurements. Indeed, the N‐H distance appears to be different for the *N*‐pyridine compounds, 2.5/2.8° in *N*‐pyridine‐carbazole versus 2.4° for **[4]C‐Py‐Cbz** whereas it is very similar for *N*‐pyrimidine compounds, 2.5° for *N*‐pyrimidine‐carbazole versus 2.4° for **[4]C‐Pm‐Cbz**.

**Figure 4:**
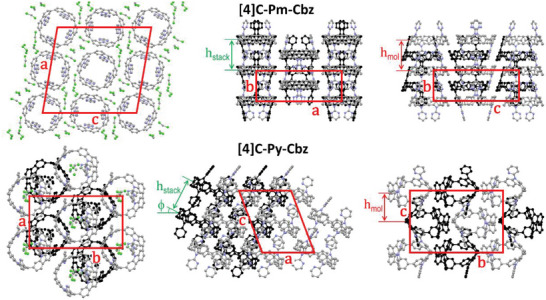
Views of the single‐crystal structure of the nanohoops: **[4]C‐Pm‐Cbz**, from left to right, views along the b‐axis, the c‐axis (molecular slices in the foreground at z = 0.0, in the background and drawn in black at z = 0.5) and the normal to (bc)‐plane (molecular slices at x = 0.0 in the foreground, in the background and drawn in black at x = 0.5); **[4]C‐Py‐Cbz**, from left to right, views along the normal to (ab)‐plane (molecular slices in the foreground at z = 0.0, in the background and drawn in black at z = 0.5), the b‐axis (molecular slices in the foreground with centroids at y = 0.0 and y = 0.2, in the background and drawn in black with centroids at y = 0.5 and y = 0.7) and the a‐axis (molecular slices in the foreground at x = 0.0, in the background and drawn in black at x = 0.5). For the sake of clarity, hydrogens are not shown and solvent molecules are only displayed for the views on the left.

The supramolecular organization of the nanohoops comes down to two‐dimensional arrangements of hollow columns, formed by the stacking of the hoop‐shaped molecules interlocking their heterocyclic substituents (**Figure** **4**). The stacking distance *h*_stack_ is logically the same for both derivatives (9.185 Å for **[4]C‐Pm‐Cbz** and 9.180 Å for **[4]C‐Py‐Cbz**), since the substituents have equivalent sizes. However, the organizations are clearly different: **[4]C‐Pm‐Cbz** molecules stack on top of each other into straight columns orthogonal to the 2D sublattice plane, while **[4]C‐Py‐Cbz** molecules stack with an out‐of‐plane tilt‐angle ϕ of 27° (in *(ac)*‐plane) and alternating lateral shifts of ± 3.8 Å (in *(bc)*‐plane). The molecular periodicity perpendicular to the sublattice *h*_mol_ is therefore equal to *h*_stack_ for the straight columns (9.185 Å) and lowered to 8.18 Å for the tilted and shifted stacks. Moreover, co‐crystallized dichloromethane (two molecules per nanohoop) occupies the voids left by the lateral shifts in **[4]C‐Py‐Cbz** stacks. The **[4]C‐Pm‐Cbz** structure also contains co‐crystallized dichloromethane (four molecules per nanohoop), which is however located in the periphery of the straight columns and swells the arrangement. Although the roughly cylindrical columns might have been arranged in a hexagonal way, the swelling reduces the number of first‐neighboring columns to 4 and lowers the symmetry to oblique. On the contrary, the solvent location in the **[4]C‐Py‐Cbz** structure preserves the approximately cylindrical shape of molecular stacks, although with dissymmetric in‐plane directions. The result is a distorted hexagonal arrangement: six first‐neighboring stacks, rectangular centered symmetry, and lattice parameter ratio of 1.766 compared to $\sqrt{3}$ for the hexagonal geometry.

The average spacing of **[4]C‐Py‐Cbz** stacks is 13.91 Å, which is only slightly larger than the equivalent cylinder size reported for [4]cyclo‑*N*‑alkyl‐2,7‐carbazoles (*D*_ring,cyl_ = 13.3–13.4 Å).^[^ ^24^ ^]^ This finding meets the expectations, since these substituents are less bulky than cyclocarbazole and constrain only a little the lateral packing. On the other hand, the alkyl substituents of [4]cyclo‑*N*‑alkyl‐2,7‐carbazoles are characterized by their strong propensity to nanosegregate from conjugated moieties.^[^ ^63^ ^]^ This feature leads to lamello‐columnar structures, in which layers of cyclocarbazole and alkyl chains alternate, while **[4]C‐Py‐Cbz** and **[4]C‐Pm‐Cbz** form molecular stacks arranged in columnar‐type structures. Furthermore, the alkyl moiety also confers softness and the ability to improve molecular organizations by adapting the chain conformation. Here, the stacks of interlocked molecules are essentially rigid but the cocrystallized solvent fills up the interstices of the self‐assembly and provides some plasticity. Conversely, the solvent‐free powder materials were found to exhibit an undefined crystallized solid state giving neither thermal events (Figure S19, Supporting Information) nor structural changes (Figure S20, Supporting Information) until the degradation started above 250 °C (Figure S1, Supporting Information). Temperature decomposition Td was determined at 434 and 436°C for **[4]C‐Py‐Cbz** and **[4]C‐Pm‐Cbz** (**Table** **1**).

**Table 1:** Photophysical and thermal properties of **[4]C‐Ph‐Cbz**, **[4]C‐Py‐Cbz**, and **[4]C‐Pm‐Cbz**.

	[4]C‐Ph‐Cbz	[4]C‐Py‐Cbz	[4]C‐Pm‐Cbz
λ_abs (_nm)^a)^	260, 295, 341	256, 305, 335, 361*	244, 286, 330, 351*
λ_em sol_ (nm)^a)^, ^b)^	501	490	495
QY_sol_ ^a)^, ^b)^, ^c)^	0.18	0.18	0.17
λ_abs film_ (nm)^d)^	367	346	354
λ_em film_ (nm)^b)^, ^d)^	474	484	489
QY_film_ ^d)^, ^e)^	0.15	0.11	0.12
τ_s_ (ns)^a)^, ^f)^	6.92	7.09	7.15
k_r_ (ns^−1^)^g)^	0.026	0.025	0.025
k_nr_ (ns^−1^)^g)^	0.118	0.116	0.115
S_1_ (eV)^h)^	2.94	3.00	3.07
T_d_ (°C)^i)^	nd	434	436

^a)^ In dichloromethane at RT;

^b)^ λ_exc_ = 330 nm for **[4]C‐Py‐Cbz** and **[4]C‐Pm‐Cbz** and λ_exc_ = 350 nm for **[4]C‐Ph‐Cbz**;

^c)^ Quantum yield (QY) determined with sulfate quinine in 1 M H_2_SO_4_ as the reference;

^d)^ In spin‐coated films;

^e)^ QY determined in the integration sphere;

^f)^ At λ_em sol_ with λ_exc_ = 310 nm;

^g)^ k_r_ = QY_sol_/τ_s_ and k_nr_ = (1/τ_s_) × (1‐QY_sol_);

^h)^ From the onset of the emission spectrum in dichloromethane;

^i)^ From TGA. *Shoulder. nd: Not determined.

### 1.3 Photophysical Properties

**Figure 5:**
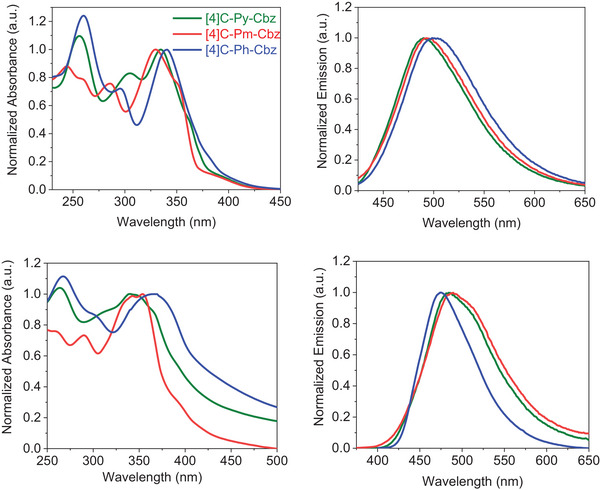
**[4]C‐Py‐Cbz** (green lines), **[4]C‐Pm‐Cbz** (red lines), and **[4]C‐Ph‐Cbz** (blue lines). Absorption in dichloromethane (top left) and in spin‐coated thin film (bottom left). Emission in dichloromethane (top right, λ_exc_ = 350 nm) and in spin‐coated thin films (bottom right, λ_exc_ = 340 nm).

**Figure 6:**
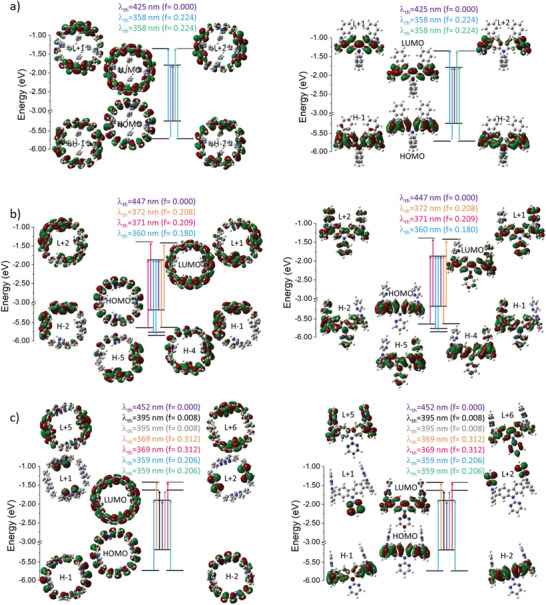
Representation of the energy levels and the molecular orbitals involved in main the electronic transitions of a) **[4]C‐Ph‐Cbz**, b) **[4]C‐Py‐Cbz**, and c) **[4]C‐Pm‐Cbz** (TD‐DFT, B3LYP/6‐311+g(d,p)), orbitals shown with an isovalue of 0.02 (e.bohr^‐3^)^−1/2.^

The three nanohoops were characterized by UV–Vis absorption spectroscopy as well as stationary and time‐resolved fluorescence in dichloromethane and in spin‐coated film, **Figure** **5**. As previously described for cyclocarbazoles substituted with alkyl chains, the absorption spectrum of **[4]C‐Ph‐Cbz** displays three bands at 260, 295, and 341 nm and a shoulder ≈400 nm. This classical shoulder is assigned to a symmetry‐forbidden HOMO‐LUMO transition in light of TD‐DFT calculations (λ_th_ = 425 nm, f = 0.000, **Figure** **6**a). The main band at ≈341 nm is due to two transitions involving the HOMO, LUMO, and the degenerated orbitals H‐1 and H‐2, L+1, and L+2, as also obtained for cyclocarbazoles substituted with alkyl chains.^[^ ^24^ ^]^ It should be noted that the phenyl substituent is not involved in these orbitals, its impact compared to alkyl chains is really weak. The case of **[4]C‐Pm‐Cbz** and **[4]C‐Py‐Cbz** is different since some orbitals are (partially) localized on the pyrimidine and pyridine substituents. First, the maximum is blue‐shifted from 341 nm in **[4]C‐Ph‐Cbz** to 335 and 330 nm in **[4]C‐Py‐Cbz** and **[4]C‐Pm‐Cbz** respectively. Thus, all orbitals of **[4]C‐Py‐Cbz** involved in the main transitions are localized both on the cyclocarbazole core and the pyridine, except for HOMO (Figure 6b). In the case of **[4]C‐Pm‐Cbz**, only the unoccupied orbitals are partially (LUMO, L+5 and L+6) or totally (L+1 and L+2) localized on the pyrimidine group (Figure 6c). In addition to the usual bands found in nanohoops, we note a shoulder at ≈351 nm for **[4]C‐Pm‐Cbz** and 361 nm for **[4]C‐Py‐Cbz**. The main band of **[4]C‐Py‐Cbz**, experimentally found at 335 nm, is assigned to a transition with two contributions (from H‐4 and H‐5 to the LUMO), and the shoulder at 361 nm is due to two transitions, each one with two distinct contributions (H‐2→LUMO, HOMO→L+2 and H‐1→LUMO, HOMO→L+1). Similarly, the main band of **[4]C‐Pm‐Cbz**, experimentally found at 330 nm, is described by two transitions (from H‐1 and H‐2 to LUMO) and the shoulder at 351 nm is due to two distinct transitions (HOMO→L+5 and HOMO→L+6). Note also, in the case of **[4]C‐Pm‐Cbz**, that two charge transfer transitions from the HOMO (localized on the cyclocarbazole core only) to the L+1 and the L+2 orbitals (localized only on the pyrimidine) are computed at λ_th_ = 395 nm (f = 0.008). But the oscillator strength is too weak and the corresponding band cannot be seen. Thus, it can be concluded that the nature of the substituents borne by the carbazole has a clear impact on the UV–Vis absorption spectrum and the nature of the transitions involved.

In emission, **[4]C‐Ph‐Cbz**, **[4]C‐Py‐Cbz** and **[4]C‐Pm‐Cbz** display a large and unresolved spectrum centered respectively at 501, 490, and 495 nm, showing, this time, the limited effect of the substituent on the nanohoop emission wavelengths. The fluorescence quantum yields QY_sol_ and the singlet lifetimes τ_s_ are also similar for the three nanohoops (≈0.18 and 7 ns respectively) leading to similar radiative and non‐radiative rates (see Table 1). Thus, the substituents borne by the nitrogen atom of the carbazoles have only a limited impact on the deactivation processes of the emissive state. This is also confirmed by solvatochromic experiments. Varying the polarity has only a very weak effect on the emission spectrum of **[4]C‐Py‐Cbz** and **[4]C‐Pm‐Cbz** (see Figure S3, Supporting Information). This behavior signs a very weak intramolecular charge transfer (ICT) between the carbazole and the grafted heterocycle.

Absorption and emission spectrum of the three nanohoops in spin‐coated thin film from a solution of THF (Figure 5, bottom) display similar maxima and similar shapes to their solution ones. They are nevertheless slightly larger and shifted in accordance with the different environments (solid vs solution). Thus, despite different supramolecular packing observed in single crystals (Figure 4), the optical properties remain in thin film similar to that in solution. This is also in accordance with the quantum yields measured in a thin film, which are in the same range as those measured in solution (**[4]C‐Ph‐Cbz**: 0.18 vs 0.15, **[4]C‐Py‐Cbz**: 0.18 vs 0.11, **[4]C‐Pm‐Cbz**: 0.17 vs 0.12, Table 1). This implies that the classical aggregation‐induced quenching, very often observed in linear materials, is absent here. This is one of the specificities of nanohoops, which could be further used to design materials with high fluorescence quantum yield in the solid state.

### 1.4 Electrochemical Properties

**Figure 7:**
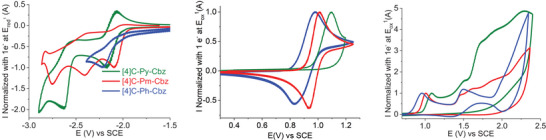
Normalized cyclic voltammograms of **[4]C‐Py‐Cbz** (green lines), **[4]C‐Pm‐Cbz** (red lines), and **[4]C‐Ph‐Cbz** (blue lines). Left: cathodic range, in DMF (0.1 M Bu_4_NPF_6_), middle and right: anodic range in CH_2_Cl_2_ (0.2 M Bu_4_NPF_6_). Sweep‐rate of 100 mV s^−1^, platinum disk (diameter 1 mm) working electrode.

The electrochemical properties of **[4]C‐Ph‐Cbz**, **[4]C‐Py‐Cbz** and **[4]C‐Pm‐Cbz** have been investigated by cyclic voltammetry (CV) in dichloromethane for oxidation (**Figure** **7**, middle and right) and in *N*, *N*‐dimethylformamide for reduction (Figure 7, left); potentials are given versus a saturated calomel electrode (SCE), **Table** **2**.

**Table 2:** Electrochemical and structural properties of **[4]C‐Ph‐Cbz**, **[4]C‐Py‐Cbz**, and **[4]C‐Pm‐Cbz**.

	[4]C‐Ph‐Cbz	[4]C‐Py‐Cbz	[4]C‐Pm‐Cbz
E_ox_ (V vs SCE)^a)^	0.96	1.08	1.01
E_red_ (V vs SCE)^b)^	−2.21	−2.17	−2.09
HOMO_EL_ (eV)^c)^	−5.22	−5.38	−5.32
LUMO_EL_ (eV)^c)^	−2.33	−2.38	−2.44
ΔE_EL_ (eV)^c)^, ^d)^	2.89	3.00	2.88
HOMO_theo_ (eV)^e)^	−5.23	−5.15	−5.16
LUMO_theo_ (eV)^e)^	−1.79	−1.87	−1.90
ΔE_theo_ (eV)^d)^, ^e)^	3.44	3.28	3.26
Mean torsion angle θ (°)^f)^	/	24.4	22.7 / 23.8
Mean displacement angle ω (°)^f)^	/	6.8	6.7 / 7.0
Mean dihedral angle γ (°)^f)^	/	36.75	1.03 / 3.00

^a)^ In dichloromethane;

^b)^ In DMF;

^c)^ From electrochemical data (see the formula in SI);

^d)^ ΔE = |HOMO – LUMO|;

^e)^ From theoretical calculations, DFT B3LYP/6‐311+G(d,p);

^f)^ From X‐ray structure.

In oxidation, the CVs of the three nanohoops are different highlighting the influence of the grafted heterocycle (Figure 7, middle and right). **[4]C‐Ph‐Cbz** presents, between 0 and 2.5 V, three oxidation waves with maxima at 0.96, 1.5 and above 2.0 V. **[4]C‐Py‐Cbz** and **[4]C‐Pm‐Cbz** present a first oxidation wave respectively at 1.08 and 1.01 V and several other waves which are less defined (≈1.5, 1.7 and 2.3 V for **[4]C‐Py‐Cbz** and 1.5, 1.6 and 2.0 V for **[4]C‐Pm‐Cbz**). As shown in Figure 7 (middle), the first oxidation wave is reversible for **[4]C‐Ph‐Cbz** and **[4]C‐Pm‐Cbz** at a sweep rate of 100 mV s^−1^ whereas that of **[4]C‐Py‐Cbz** is irreversible (partial reversibility is observed at 2 V s^−1^, Figure S16, Supporting Information). This indicates a different reactivity of the cation‐radical of the three nanohoops, directly correlated to the nature of the substituent. Without going in‐depth into the reactivity of these charged species, it must be recalled that electroactive deposits have been previously obtained during the oxidation of alkylated cyclocarbazoles. In the present study, the polymerization process only occurs from the second oxidation wave for **[4]C‐Ph‐Cbz** and **[4]C‐Pm‐Cbz** whereas it is observed from the first oxidation process for **[4]C‐Py‐Cbz** (Figures S13–S15, Supporting Information). For **[4]C‐Pm‐Cbz** and **[4]C‐Py‐Cbz**, the polymerization generates a non‐electroactive deposit which induces the progressive decrease of the current in the potential range of electrodeposition whereas electroactive deposits are obtained from **[4]C‐Ph‐Cbz**, as previously described for different alkylated cyclocarbazoles.^[^ ^24^, ^52^, ^56^ ^]^ This study shows that the substituents grafted on the carbazoles play a major role both in the reactivity of the cation‐radical of the nanohoops and in the nature of the deposit obtained by electropolymerization. As anodic electrodeposition is an efficient method to generate materials for various applications (i.e., catalysis,^[^ ^64^, ^65^ ^]^ detection,^[^ ^66^ ^]^ electrochromic devices^[^ ^67^ ^]^…), this may open new perspectives to nanohoops.

The HOMO levels have been evaluated from the onset oxidation potential at −5.22 eV for **[4]C‐Ph‐Cbz**, −5.38 eV for **[4]C‐Py‐Cbz** and −5.32 eV for **[4]C‐Pm‐Cbz**. As expected, the HOMO of **[4]C‐Ph‐Cbz** with a phenyl substituent attached is higher than those of **[4]C‐Py‐Cbz** and **[4]C‐Pm‐Cbz** incorporating an electron‐withdrawing group. However, the differences in the HOMO levels of **[4]C‐Py‐Cbz** and **[4]C‐Pm‐Cbz** are not fully driven by the electron‐withdrawing strength of the substituents (which are not involved in the HOMO delocalization according to quantum chemical calculations). Indeed, following the electron‐withdrawing strength of the substituent, a lower HOMO level for **[4]C‐Pm‐Cbz** compared to **[4]C‐Py‐Cbz** was expected. Actually, as already observed for other nanohoops,^[^ ^3^, ^24^, ^52^, ^57^, ^58^, ^59^, ^60^ ^]^ the evolution of the HOMO levels is also linked to the evolution of the geometrical parameters. The carbazoles in **[4]C‐Pm‐Cbz** are less twisted (smaller torsion angle θ) and thus more conjugated than in **[4]C‐Py‐Cbz**. This is the reason why the HOMO of **[4]C‐Pm‐Cbz** is higher than that of **[4]C‐Py‐Cbz** despite its more electron‐withdrawing substituent. This specificity of nanohoops appears herein particularly interesting to differently tune the HOMO and LUMO energy levels.

CVs recorded between −1.5 and −3.0 V present different reduction processes for the three nanohoops: two reduction waves with maxima at −2.17 and −2.63 V for **[4]C‐Py‐Cbz** and three waves with maxima at −2.09, −2.40, and −2.75 V for **[4]C‐Pm‐Cbz** (Figure 7, left). Due to a very low solubility of **[4]C‐Ph‐Cbz** in DMF, it was difficult to pinpoint its different reduction waves and only one irreversible reduction wave was recorded with a maximum of −2.21 V. The LUMO levels have been evaluated from their onset reduction potentials at −2.33 eV for **[4]C‐Ph‐Cbz**, −2.38 eV for **[4]C‐Py‐Cbz** and −2.44 eV for **[4]C‐Pm‐Cbz**. A similar evolution is also found by the calculations: −1.79 eV for **[4]C‐Ph‐Cbz**, −1.87 eV for **[4]C‐Py‐Cbz**, and −1.90 eV for **[4]C‐Pm‐Cbz**. The theoretical results also show that the LUMO of the three nanohoops is centered on the carbazoles with the participation of the substituent, which increases with their electron‐withdrawing ability. Due to a more electron‐withdrawing character of the pyrimidine compared to the pyridine and the smaller dihedral angle γ between the carbazole and its substituent in **[4]C‐Pm‐Cbz** compared to **[4]C‐Py‐Cbz**, the LUMO level of **[4]C‐Pm‐Cbz** is lowered compared to that of **[4]C‐Py‐Cbz**. Furthermore, according to the link between structural parameters and orbital levels, the decrease of the torsion angle from **[4]C‐Py‐Cbz** to **[4]C‐Pm‐Cbz** should induce a decrease in the LUMO level. Thus, both the structural parameters and the electron‐withdrawing ability of the substituents drive the LUMO level energy in the same direction.

### 1.5 Charge Transport Properties

**[4]C‐Py‐Cbz** and **[4]C‐Pm‐Cbz** were finally integrated into OFET‐type structures. From the transfer characteristics, the key electrical parameters of the transistors were extracted. Some of these parameters provide information on the electrical performance of the device, while others allow us to evaluate the degree of organization in the thin film. The raw device performances are established with the values of field‐effect mobility in the linear regime (µ_FE_lin_) which are affected by the structural organization in the thin film. The more disorganized the thin film, the lower the µ_FE_lin_ value due to the creation of an increased energetic disorder generating traps for the charge carriers in the tail of the distribution. It is therefore difficult to experimentally determine whether one molecule is intrinsically more efficient than another since the organization of the layer depends on the deposition parameters. Similarly, the threshold voltage (V_TH_) is linked to the device's ability to accumulate charges at the insulator/semiconductor interface and is therefore highly dependent on the concentration of defects at this interface.

To better analyze the performance of the two nanohoops, µ_FE_lin_ and V_TH_ will be herein combined with saturated field effect mobilities (µ_FE_sat_) and subthreshold slopes (SS) to shed light on the maximum carrier velocity and the quality of the insulator/semiconductor interface, respectively. In addition to these indicators, temperature and electrical stability measurements were carried out to extract activation energies (Ea) and defect‐related parameters, respectively. Ea is used to assess the energy required for a carrier to access the next conduction site for a transport operating in a hopping regime. The more the semiconductor is organized in a solid phase, the lower the Ea. Electrical stress can be used to characterize defects in terms of energy depth and average carrier trapping time.

**Figure 8:**
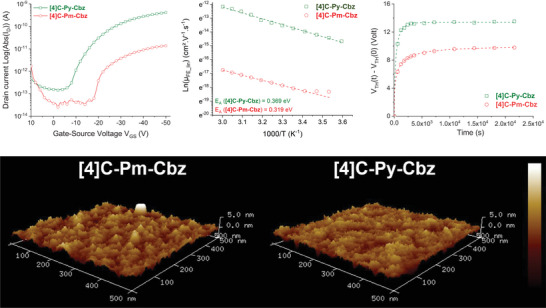
**[4]C‐Py‐Cbz** and **[4]C‐Pm‐Cbz**‐based OFETs. Top. Transfer Characteristics (Left), linear field‐effect mobility activation energy (Middle), and time‐dependent variations in V_TH_ under gate‐bias stress (Right). Bottom. AFM measurements.

**[4]C‐Py‐Cbz** and **[4]C‐Pm‐Cbz** have been successfully incorporated into a Bottom‐Gate Bottom‐Contact transistor structure (see device architecture in Figure S21, Supporting Information). The transfer characteristics clearly show higher electrical performance for **[4]C‐Py‐Cbz** (**Figure** **8**, Top‐left).

Linear field effect mobilities were extracted and evaluated at µ_FE_lin_ = 7 × 10^−7^ cm^2^ V^−1^ s^−1^ and 1.2 × 10^−8^ cm^2^ V^−1^ s^−1^ for **[4]C‐Py‐Cbz** and **[4]C‐Pm‐Cbz** respectively. V_TH_ shows the same trend, with a difference of 3 V between **[4]C‐Py‐Cbz** (V_TH_ = −20.8 V) and **[4]C‐Pm‐Cbz** (V_TH_ = −23.9 V). Surprisingly, observing the transfer characteristics, **[4]C‐Pm‐Cbz** appears to be better organized as a thin film than **[4]C‐Py‐Cbz**. Indeed, when analyzing field‐effect mobility values in the saturated regime, **[4]C‐Pm‐Cbz** appears to be at peak performance, with a mobility µ_FE_sat_ = 2.5 × 10^−8^ cm^2^ V^−1^ s^−1^, a value very close to field‐effect mobility in the linear regime (µ_FE_lin_ = 1.2 × 10^−8^cm^2^ V^−1^ s^−1^). Oppositely, the performance of **[4]C‐Py‐Cbz**‐based transistor could be increased, since the field‐effect mobility in the saturated regime is much higher, µ_FE_sat_ = 3.4 × 10^−6^cm^2^ V^−1^ s^−1^ than in the linear one. This clearly shows that the mobility of **[4]C‐Py‐Cbz** could be further improved in the future and two levels of optimization could be considered. The first deals with the interface between the insulator and semiconductor, since SS of the **[4]C‐Py‐Cbz**‐based transistor was evaluated at 3.4 V/dec whereas that of **[4]C‐Pm‐Cbz** was measured at 2.6 V/dec. The second optimization targets the structural organization of the semiconductor layer, given the differences between linear and saturated FE mobilities.

To confirm this second assertion, electrical temperature measurements coupled with AFM roughness measurements were carried out. While AFM measurements (Figure 8, Bottom) show a slightly lower relative average roughness (R_a_) for the **[4]C‐Pm‐Cbz**‐based thin film (R_a_ = 0.717 nm) compared to **[4]C‐Py‐Cbz** (R_a_ = 0.765 nm), the activation energy measurements provide more useful information. Indeed, measurements of linear FE mobilities as a function of temperature allow to extraction of the activation energy E_a_. In the hopping regime, the conduction takes place via carrier jumps between localized states and the transfer rate can be described by Marcus theory, see below.^[^ ^68^ ^]^ The activation barrier Ea for a hopping process is given by Ea = (ΔE + λ)^2^/4λ, with ΔE = E_f_–E_i_ the difference between the initial and final site energies and λ the reorganization energy typically dominated by the geometric changes in the molecule when going from the neutral to the charged state and vice versa.^[^ ^69^ ^]^

The logarithm plot of FE mobility ln(µ_FE_) evolves linearly with the inverse of the temperature (1000/T, Figure 8, Top‐middle) showing the better organization of **[4]C‐Pm‐Cbz** for which an E_a_ value of 319 meV is determined, lower than that of **[4]C‐Py‐Cbz**, E_a_ = 369 meV. On the basis of the reorganization energy λ computed for the alkylated cyclocarbazoles (322 meV)^[^ ^70^ ^]^ and the experimental activation energy Ea extracted from the temperature‐dependent measurements, we can deduce a typical energetic disorder ΔE of 319 meV for **[4]C‐Pm‐Cbz** and 367 meV for **[4]C‐Py‐Cbz**.

Interestingly, the most electrically efficient device does not necessarily have the best‐organized semiconductor layer. This is an interesting feature, which can be correlated to the transfer integrals computed for the crystalline structures, as discussed below. On the other hand, this analysis shows that the **[4]C‐Py‐Cbz**‐based transistor can be optimized in the future and its electrical performance significantly improved.

Finally, Gate‐Bias Stress measurements were carried out to determine the impact of defects on the transistor's electrical behavior over time (Figure 8, Top‐right). The results confirm previous observations, with shallower defects in **[4]C‐Pm‐Cbz** (β = 0.39 eV, definition in SI) than in **[4]C‐Py‐Cbz** (β = 0.63 eV), longer average trapping times for **[4]C‐Pm‐Cbz** (564.7 and 319.8 s respectively for **[4]C‐Pm‐Cbz** and **[4]C‐Py‐Cbz**) and higher variations in maximum threshold voltages in **[4]C‐Py‐Cbz**‐based transistors (ΔV_THmax_ = 13.39 V for **[4]C‐Py‐Cbz** and 9.92 V for **[4]C‐Pm‐Cbz**).

### 1.6 Molecular Modelling

**Figure 9:**
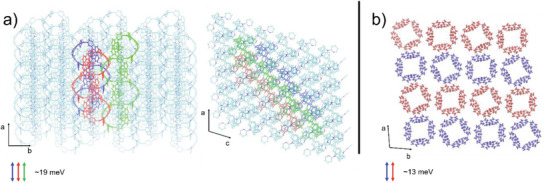
a) Transfer integrals in the experimental crystal structure of **[4]C‐Py‐Cbz**. The HOMO‐HOMO couplings between the colored molecules are 19 meV, while the values for all other dimers do not exceed 7 meV. b) Transfer integrals in the experimental crystal structure of **[4]C‐Pm‐Cbz**. The HOMO‐HOMO coupling between the red or blue nanohoops is ≈13 meV but is close to zero across.

To shed light on the intrinsic hole transport properties of the two nanohoops, we computed the transfer integrals from their experimental crystalline structures. Their small magnitude (roughly between 10 and 20 meV) indicates that the charge transport will most likely follow the hopping regime, as assumed in the previous section.^[^ ^71^ ^]^ Within Marcus theory, the transfer rate can thus be expressed as:

(1) $$k_{ET}=\frac{2\pi {\left|J_{ab}\right|}^{2}}{\hbar \sqrt{\pi \lambda k_{B}T}}e^{-\frac{{\left(\Delta E+\lambda \right)}^{2}}{4\lambda k_{B}T}}$$

where J_ab_ is the transfer integral between sites a and b. In this framework, the transfer integral typically plays the predominant role in charge transport in the bulk system. The relative magnitude of this parameter between different dimers (in different directions) will decide the dimensionality of the transport. The more easily the charge can move in all directions, the better emancipated it is from the random orientation of the crystal islands in the OFET with regard to the Source‐Drain electric field, point defects, internal grain boundaries, etc… It entails that the optimal configuration for high charge transport is that all transfer integrals participating in the charge transport must be of similar magnitude, and in all directions. This idea is also valid in high‐performing OSCs such as rubrene or dinaphtothienothiophene, although the sign of the relative transfer integrals also plays a role.^[^ ^72^ ^]^ In **Figure** **9**, we display the transfer integrals, as computed from the crystalline experimental structure, using a fragment approach as implemented in the ADF package,^[^ ^73^ ^]^ with the B3LYP functional and a DZ basis set.

Both materials exhibit a predominant 1D type transport, which is quite detrimental for charge transport properties and explains in part the quite low measured FET mobilities. Nevertheless, **[4]C‐Py‐Cbz** presents overall slightly higher transfer integrals, implying that the charge will move faster along the a‐axis. The other advantage of **[4]C‐Py‐Cbz** is the ratio between its predominant transport direction and other possible paths. A hole in **[4]C‐Py‐Cbz** has a higher chance of hopping on a neighboring column than in **[4]C‐Pm‐Cbz**. This effect favors **[4]C‐Py‐Cbz** as a better semiconductor and participates in explaining the differences in the mobility in the saturated regime.

## 2 Conclusion

We report in this work our investigations on the impact of the incorporation of functional units (electron‐withdrawing pyridine and pyrimidine groups) on the nitrogen atoms of electron‐rich [4]cyclo‐2,7‐carbazoles. Based on a dual experimental and theoretical approach, the synthesis and the electrochemical, photophysical, thermal, and structural properties of the two nanohoops **[4]C‐Py‐Cbz** and **[4]C‐Pm‐Cbz** are described and compared to a model compound possessing a phenyl unit on the nitrogen atom, **[4]C‐Ph‐Cbz**. As the synthesis of nanohoops remains a key feature in the field, especially when an application is targeted, the tetra‐platinum approach from the dipinacol carbazole intermediate has been optimized and a high yield of over 50% has been reached. This is a key point for further device incorporation. The structure‐properties relationship study shows that both structural parameters of the nanohoop and the electron‐withdrawing ability of the substituents are involved in the electrochemical properties and the resulting HOMO/LUMO energy levels. Thus, the pending substituent modifies the structural characteristics of the nanohoops and particularly the crystal packing, which in turn modifies the charge carrier mobilities. Incorporation of the nanohoops in electronic devices has been performed and it has been shown that the most electrically efficient OFETs are obtained with **[4]C‐Py‐Cbz** although this compound does not present the best‐organized semiconductor layer. These experimental data have been finally confronted with the electronic couplings between the nanohoops determined at the DFT level in the single crystal structures and have highlighted a fundamental origin in the difference of charge transport properties. **[4]C‐Py‐Cbz** has the advantage of a more 2D‐like transport character than **[4]C‐Pm‐Cbz**, which alleviates the impact of defects and structural organization. As the recent first incorporations of nanohoops in electronic devices have appeared very promising, particularly in OLEDs,^[^ ^1^ ^]^ going deeper into their rational designs and well understanding the specificity of this class of materials are the next challenges to construct, in the near future, efficient and versatile organic materials. We are convinced that systematic studies allowing us to evaluate the evolution of the electronic and structural properties as a function of the grafted substituent (electron‐rich and/or ‐poor functional units) are needed to improve notably the charge transport properties of nanohoops. We are putting our effort in this regard in order to be able to design a nanohoop with high charge mobility values.

## Conflict of Interest

The authors declare no conflict of interest.

## Supporting information

Supporting Information

## Data Availability

The data that support the findings of this study are available in the supplementary material of this article.
