# Supplementary material for: Grafting Electron‐Accepting Fragments on [4]cyclo‐2,7‐carbazole Scaffold: Tuning the Structural and Electronic Properties of Nanohoops
Source: Adv Sci (Weinh). 2024 Jan 22;11(13):2309115. doi: 10.1002/advs.202309115 (PMC10987112; doi:10.1002/advs.202309115)
Supplement: Supplementary file 1 — Supporting Information [file ADVS-11-2309115-s001.pdf]

ADVANCED SCIENCE
Open Access

Supporting Information

for *Adv. Sci.*, DOI 10.1002/advs.202309115

Grafting Electron-Accepting Fragments on [4]cyclo-2,7-carbazole Scaffold: Tuning the Structural and Electronic Properties of Nanohoops

Clément Brouillac, Nemo McIntosh, Benoît Heinrich, Olivier Jeannin, Olivier De Sagazan, Nathalie Coulon, Joëlle Rault-Berthelot, Jérôme Cornil, Emmanuel Jacques, Cassandre Quinton and Cyril Poriel**

Supporting information for

Grafting electron-accepting fragments on [4]cyclo-2,7-carbazole scaffold: Tuning the structural and electronic properties of nanohoops

Clément Brouillac,[a] Nemo McIntosh,[b] Benoît Heinrich,[c] Olivier Jeannin,[a] Olivier De Sagazan,[d] Nathalie Coulon,[d] Joëlle Rault-Berthelot,[a] Jérôme Cornil,[b] Emmanuel Jacques,[d] Cassandre Quinton[a*] and Cyril Poriel[a*]

[a] Univ Rennes, CNRS, ISCR-UMR 6226, F-35000 Rennes, France

[b] Laboratory for Chemistry of Novel Materials, University of Mons, Mons, Belgium

[c] Institut de Physique et Chimie des Matériaux de Strasbourg (IPCMS), UMR 7504, CNRS-Université de Strasbourg, 23 rue du Loess, BP 43, 67034 Strasbourg Cedex 2, France

[d] Univ Rennes, CNRS, IETR-UMR 6164, F-35000 Rennes, France

email: cyril.poriel@univ-rennes1.fr, cassandre.quinton@univ-rennes1.fr

1 General information 3
Synthesis 3
Spectroscopic studies 3
Electrochemical studies 4
Theoretical modelling 4
Thermal analysis 4
Atomic force microscopy measurement 5
Organic field effect transistors (OFETs) fabrication and characterization 5
2 Synthetic procedures 6
2,7-dibromo-9-phenyl-9H-carbazole (**1a**) 6
General procedure for Ullmann coupling reaction. 7
2,7-dibromo-9-(pyridin-2-yl)-9H-carbazole (**1b**) 7
2,7-dibromo-9-(pyrimidin-2-yl)-9H-carbazole (**1c**) 7
General procedure for the borylation reaction. 8
9-phenyl-2,7-bis(4,4,5,5-tetramethyl-1,3,2-dioxaborolan-2-yl)-9H-carbazole (**2a**) 8
9-(pyridin-2-yl)-2,7-bis(4,4,5,5-tetramethyl-1,3,2-dioxaborolan-2-yl)-9H-carbazole (**2b**) ... 9
9-(pyrimidin-2-yl)-2,7-bis(4,4,5,5-tetramethyl-1,3,2-dioxaborolan-2-yl)-9H-carbazole (**2c**)9
General procedure for nanohoops synthesis. 10
[4]cyclo-*N*-pyridine-2,7-carbazole **[4]C-Py-Cbz** 10
[4]cyclo-*N*-pyrimidine-2,7-carbazole **[4]C-Pm-Cbz** 11
[4]Cyclo-*N*-phenyl-2,7-carbazole **[4]C-Ph-Cbz** 11
Optimization for transmetallation step in nanohoops synthesis 12
3 Thermal Properties 13
4 Photophysical properties 14
5 Structural properties 21
Displacement angle 22
Torsion angle 23
6 S/WAXS measurement 24
7 Molecular modelling 25
8 Organic field effect transistor measurements 37
9 AFM 39
10 X-ray diffraction structures and tables 40
11 Copy of NMR Spectra 41
12 References 67

1 General information

Synthesis

All manipulations of oxygen and moisture-sensitive materials were conducted with a standard Schlenk technique. All glassware was kept in an oven at 80°C. Argon atmosphere was generated by three repetitive cycles of vacuum/Argon using a Schlenk ramp. Commercially available reagents and solvents were used without further purification other than those detailed below. THF was obtained through a PURE SOLV™ solvent purification system. Light petroleum refers to the fraction with bp 40-60°C. Analytical thin layer chromatography was carried out using aluminum backed plates coated with Merck Kieselgel 60 GF254 and visualized under UV light (at 254 and 360 nm). Flash chromatography was carried out using Teledyne Isco CombiFlash® Rf 400 (UV detection 200-360nm), over standard silica cartridges (Redisep® Isco or Puriflash® columns Interchim).^{1}H and ^{13}C NMR spectra were recorded using Bruker 300 MHz instruments (^{1}H frequency, corresponding ^{13}C frequency: 75 MHz); chemical shifts were recorded in ppm and J values in Hz. The residual signals for the NMR solvents used are 5.32 ppm (proton) and 54.00 ppm (carbon) for CD_2Cl_2 and 7.26 ppm (proton) and 77,16 ppm (carbon) for $CDCl_3$.[1] The following abbreviations have been used for the NMR assignment: s for singlet, d for doublet, t for triplet, q for quadruplet, dd for doublet-doublet and m for multiplet. High resolution mass spectra were recorded at the Centre Régional de Mesures Physiques de l'Ouest (CRMPO-Rennes) on a Thermo Ficher Q-Exactive instrument or a Bruker MaXis 4G or a Bruker Ultraflex III.

Spectroscopic studies

Cyclohexane (spectroscopic grade, Acros), THF (spectroscopic grade, Acros), dichloromethane (spectroscopic grade, Acros), acetronitrile (spectroscopic grade, Acros), ethyl acetate (spectroscopic grade, Acros), 2-MeTHF (spectroscopic grade, Sigma Aldrich), 1 N solution of sulfuric acid in water (Standard solution, Alfa Aesar), and quinine sulfate dihydrate (99+%, ACROS organics) were used without further purification.

UV-visible spectra were recorded using an UV-Visible spectrophotometer JASCO-V630BIO.

Emission spectra were recorded with a HORIBA Scientific Fluoromax-4 equipped with a Xenon lamp and a JASCO FP-8300. Conversion in electron-volt was obtained with the following formula:

$$E(eV) = \frac{h\nu}{\lambda}$$

with h = 6.62607×10^{-34} J.s, C = 2.99792×10^{17} nm.s^{-1} and 1 eV = 1.60218×10^{-19} J. This equation can be simplified as:

$$E(eV) = \frac{1239.84}{\lambda}$$

with λ formulated in nm.

Quantum yields in solution (QYsol) were calculated relative to quinine sulfate (QYref = 0.546 in H_2SO_4 1 N). QYsol was determined according to the following equation,

$$QYsol = QYref \times \frac{Grad_s}{Grad_r} \times \left(\frac{\eta_s}{\eta_r}\right)^2$$

where subscripts *s* and *r* refer respectively to the sample and reference, *Grad* is the gradient from the plot of integrated fluorescence intensity *vs* absorbance, η is the refracting index of the solvent (η_s = 1.421 for dichloromethane). Five solutions of different concentration (A < 0.1) of the sample and five solutions of the reference (quinine sulfate) were prepared. The integrated area of the fluorescence peak was plotted against the absorbance at the excitation wavelength for both the sample and reference. The gradients of these plots were then injected in the equation to calculate the reported quantum yield value for the sample.

Absolute quantum yields of the films were recorded using a deported HORIBA Scientific Quanta-Phi integrating sphere linked to the Fluoromax-4.

Emission decay measurements were carried out on the HORIBA Scientific Fluoromax-4 equipped with its TCSPC pulsed source interface.

Spin-coated films were prepared from a 1mg/mL in THF solution using a Labspins Tournette from Süss Microtec.

Electrochemical studies

Electrochemical experiments were performed under argon atmosphere using a Pt disk electrode (diameter 1 mm). The counter electrode was a vitreous carbon rod. The reference electrode was either a silver wire in a 0.1 M $AgNO_3$ solution in CH_3CN for the studies in oxidation or a Silver wire coated by a thin film of AgI (silver(I)iodide) in a 0.1 M Bu_4NI solution in DMF for the studies in reduction. Ferrocene was added to the electrolyte solution at the end of a series of experiments. The ferrocene/ferrocenium (Fc/Fc^+) couple served as internal standard. The three electrodes cell was connected either to a PAR Model 273 potentiostat/galvanostat (PAR, EG&G, USA) monitored with the ECHEM Software or to a potentiostat/galvanostat (Autolab/PGSTAT101) monitored with the Nova 2.1 Software. Activated Al_2O_3 was added in the electrolytic solution to remove excess moisture. For a further comparison of the electrochemical and optical properties, all potentials are referred to the SCE electrode that was calibrated at - 0.405 V *vs.* Fc/Fc^+ system. Following the work of Jenekhe,[2] we estimated the electron affinity (EA) or lowest unoccupied molecular orbital (LUMO) and the ionization potential (IP) or highest occupied molecular orbital (HOMO) from the redox data. The LUMO level was calculated from: LUMO (eV) = -[E_{onset}^{red} (*vs* SCE) + 4.4]. Similarly the HOMO level was calculated from: HOMO (eV) = -[E_{onset}^{ox} (*vs* SCE) + 4.4], based on a SCE energy level of 4.4 eV relative to the vacuum. The electrochemical gap was calculated from: ΔE^{el} =|HOMO-LUMO| (in eV).

Theoretical modelling

Geometry optimization of the fundamental state (S_0) was performed using Density Functional Theory (DFT) calculations using the B3LYP functional and the 6-31G(d) basis set. All stationary points were characterized as minima by analytical frequency calculations.

Optical transition diagrams were obtained through TD-DFT calculations performed using the B3LYP functionals and the 6-311+G(d,p) basis set from the geometry of S0.

This work was granted access to the HPC resources of CEA-TGCC under the allocation 2022-AD010814136 awarded by GENCI. Figures were generated with GaussView 6.0 and GaussSum 3.0.

Thermal analysis

Thermal Gravimetric Analysis (TGA) was carried out by using a Q50 apparatus from TA instruments. TGA curved were measured at 5°C/min under air. Differential Scanning

Calorimetry (DSC) was carried out by using a DSC Q1000 from TA instrument. DSC traces were measured at 5°C/min, 3 heating/cooling cycles were successively carried out under air.

The S/WAXS patterns were obtained with a transmission Guinier-like geometry. A linear focalized monochromatic Cu Kα1 beam (λ = 1.5405 Å) was obtained using a sealed-tube generator (600 W) equipped with a bent quartz monochromator. The samples were filled in home-made sealed cells of 1 mm path. The sample temperature was controlled within ±0.01 °C, and exposure times were varied from 4 to 24 h. The patterns were recorded with a curved Inel CPS120 counter gas-filled detector (periodicities up to 90 Å) and on image plates scanned by Amersham Typhoon IP with 25 μm resolution (periodicities up to 120 Å). I(2q) profiles were obtained from images, by using home-developed software.

Atomic force microscopy measurement

AFM images were recorded on the Bruker Multimode 8 using PeakForce Tapping with a resolution of 512 x 512 pixels on a 500 nm × 500 nm surface. Scan rate has been set at 1 Hz. Each scan line in the image was scanned from left to right (trace) and from right to left (retrace). The observed topographic features were verified for their consistency between trace and retrace images. Amplitude set point was set to 1.68 mV and the drive amplitude was set to 10.38 mV. Roughness has been extracted from these images using Nano Scope Analysis 1.8 after a clean image treatment.

Organic field effect transistors (OFETs) fabrication and characterization

The fabrication process is described as follows: 150 nm thick aluminium layer was evaporated and patterned by conventional photolithography on 5 × 5 cm² rigid glass substrate. Then, SU-8 2000.5 Photoresist from Microchem[3] was then spin-coated in order to obtain a 400 nm thick layer. 50 nm thick gold layer was then thermally evaporated and patterned by photolithography. Finally, the OSCs were deposited by evaporation under vacuum as a 40 nm thick layer.

Current/voltage characteristics

The evaluation of carrier mobility has been made using the transistor structure without applying any gate-source voltage. Thus, lateral current between drain and source has been measured according to drain-source voltage.

Transfer Characteristics:

The transfer characteristics correspond to the measurement of drain current I_D as a function of gate-source voltage V_{GS} at constant drain-source voltage V_{DS}. Current drain is represented in logarithmic scale. This measurement is carried out with low and high V_{DS} corresponding to linear and saturated regimes of the OFET. These measurements have been carried out using Keithley semiconductor characterization system 2636a.

Field effect mobility: In the linear regime ($V_{DS} \leq V_{GS}$-V_{TH}), the drain current I_D is given by:

$$I_D = \frac{WC_i}{L} \mu_{FE\_Lin}(V_{GS} - V_{TH})V_{DS}$$

Where W and L are the channel width and length, respectively. C_i is the capacitance per unit area of the insulator, μ_{FE_lin} is the field-effect mobility in the linear regime, respectively. V_{GS}

and V_{DS} are the gate-source voltage and drain-source voltage, respectively. V_{TH} is the threshold voltage. Field-effect mobility in the linear regime can be expressed by:

$$\mu_{FE\_Lin} = \frac{L}{WC_iV_{DS}}\frac{\partial I_D}{\partial V_{GS}}$$

To evaluate the field-effect mobility in the saturation regime ($V_{DS} \geq V_{GS}$-V_{TH}), the drain current I_D is given by:

$$I_D = \frac{WC_i}{L}\mu_{FE\_Sat}(V_{GS} - V_{TH})^2$$

Field-effect mobility in the saturation regime can be expressed by:

$$\mu_{FE\_Sat} = \frac{2L}{WC_i}\left(\frac{\partial\sqrt{I_D}}{\partial V_{GS}}\right)^2$$

Carrier mobility:

For the organic semiconductor, the carrier mobility is extracted using the Mott-Gurney formalism in the area where m = 2 where the traps are no longer involved in the electrical conduction through the semiconducting layer.

$$J_{SCLC} = \frac{9}{8}\varepsilon_0\varepsilon_r\Theta\mu\frac{V^2}{L^3} \qquad (1)$$

$$\Theta = \frac{n_f}{n_f+n_t} \qquad (2)$$

$$\mathrm{m} = \frac{\partial \mathrm{Log(J)}}{\partial \mathrm{Log(V)}} \qquad (3)$$

2 Synthetic procedures

2,7-dibromo-9-phenyl-9H-carbazole (**1a**)

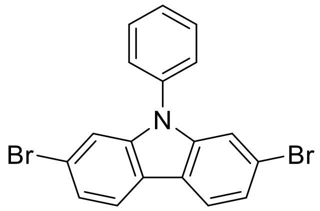

2,7-Dibromo-9H-carbazole (5.00 g, 15.4 mmol, 1.0 eq), iodobenzene (2.07 mL, 18.5 mmol, 1.2 eq), CuI (0.59 g, 3.08 mmol, 0.2 eq), K_2CO_3 (6.38 g, 46.2 mmol, 3.0 eq) and phenantroline (0.55 g, 3.08 mmol, 0.2 eq) were dissolved in DMF (45 mL). The resulting mixture was refluxed (155°C) under argon atmosphere for 36 h. The reaction mixture was concentrated under reduced pressure. The crude was dissolved in CH_2Cl_2 and filtered with a pad of Celite®. Solvent was then evaporated under reduced pressure and the product was purified with flash chromatography on silica gel [column conditions: silica cartridge (120 g); solid deposit on Celite®; λdetection: (254 nm, 280 nm); petroleum ether in 60 min at 80 mL/min], giving the

title compound as a white powder (4.08 g, **66%**). ^{1}H NMR (300 MHz, $CDCl_3$) δ 7.94 (d, *J* = 8.3 Hz, 2H), 7.68 – 7.60 (m, 2H), 7.56 – 7.46 (m, 5H), 7.40 (dd, *J* = 8.3, 1.7 Hz, 2H). Consistent with previously reported data[4]

General procedure for Ullmann coupling reaction.

2,7-Dibromo-9H-carbazole (1.0 eq), aromatic iodide (1.2 eq), Cu_2O (0.2 eq), K_3PO_4 (3.0 eq) and *N,N,N,N*-tetramethylethylenediamine (0.2 eq) were dissolved in *o*-dichlorobenzene (3 mL/mmol). The resulting mixture was refluxed (185°C) under argon atmosphere for 36 h. The reaction mixture was concentrated under reduced pressure and the crude was dissolved in CH_2Cl_2 and filtered with a pad of Celite®. Solvent was then evaporated under reduced pressure and the product was purified with flash chromatography on silica gel.

2,7-dibromo-9-(pyridin-2-yl)-9H-carbazole (**1b**)

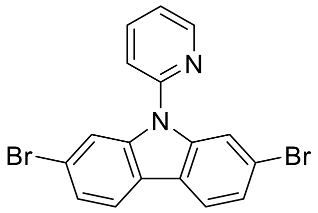

The title compound was synthesized using the general procedure for Ullmann coupling reaction with 2,7-dibromo-9H-carbazole (3.00 g, 9.23 mmol, 1.0 eq), 2-iodopyridine (1.18 mL, 11.1 mmol, 1.2 eq), Cu_2O (0.26 g, 1.85 mmol, 0.2 eq), K_3PO_4 (5.88 g, 27.7 mmol, 3.0 eq) and *N,N,N,N*-tetramethylethylenediamine (0.28 mL, 1.85 mmol, 0.2 eq) in *o*-dichlorobenzene (30 mL).

After purification with flash chromatography on silica gel [column conditions: silica cartridge (40 g); solid deposit on Celite®; λdetection: (254 nm, 280 nm); gradient petroleum ether/CH_2Cl_2 from 10% to 50% in 60 min at 40 mL/min], a colorless powder was obtained (3.62 g, **98%**).^{1}H NMR (300 MHz, CD_2Cl_2) δ 8.74 (ddd, *J* = 4.9, 2.0, 0.9 Hz, 1H), 8.03 – 7.94 (m, 5H), 7.61 (dt, *J* = 8.0, 0.9 Hz, 1H), 7.48 – 7.36 (m, 3H). ^{13}C NMR (75 MHz, CD_2Cl_2) δ 150.63, 149.92, 140.51, 138.97, 124.38, 122.50, 122.15, 121.34, 120.01, 119.05, 114.45. HRMS (ASAP, 150 °C): Found [M+H]+, 400.928, $C_{17}H_{11}N_2Br_2$ required 400.928.

2,7-dibromo-9-(pyrimidin-2-yl)-9H-carbazole (**1c**)

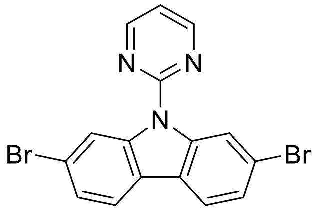

The title compound was synthesized using the general procedure for Ullmann coupling reaction with 2,7-dibromo-9H-carbazole (5.00 g, 15.4 mmol, 1.0 eq), 2-iodopyrimidine (3.80 g, 18.5 mmol, 1.2 eq), Cu_2O (0.44 g, 3.08 mmol, 0.2 eq), K_3PO_4 (9.80 g, 46.2 mmol, 3.0 eq) and

N,*N*,*N*,*N*-tetramethylethylenediamine (0.46 mL, 3.08 mmol, 0.2 eq) in *o*-dichlorobenzene (50 mL).

After purification with flash chromatography on silica gel [column conditions: silica cartridge (80 g); solid deposit on Celite®; λdetection: (254 nm, 280 nm); gradient petroleum ether/CH_2Cl_2 from 10% to 50% in 60 min at 40 mL/min], a colorless powder was obtained (4.76 g, **77%**).^{1}H NMR (300 MHz, CD_2Cl_2) δ 9.11 (dd, *J* = 1.8, 0.5 Hz, 2H), 8.88 (d, *J* = 4.8 Hz, 2H), 7.91 (d, *J* = 8.3 Hz, 2H), 7.50 (ddd, *J* = 8.3, 1.7, 0.6 Hz, 2H), 7.22 (td, *J* = 4.8, 0.6 Hz, 1H). ^{13}C NMR (75 MHz, CD_2Cl_2) δ 158.55, 158.09, 139.89, 125.67, 123.95, 120.55, 120.41, 119.64, 116.86. HRMS (ASAP, 150 °C): Found [M+H]+, 401.923, $C_{16}H_{10}N_3Br_2$ required 401.924.

General procedure for the borylation reaction.

(Di)-brominated *N*-substituted carbazole (1.0 eq) was dissolved in dry THF, *n*-BuLi (2.50 M in hexanes, 1.2 eq per bromine atom) was added dropwise at -78 °C under argon atmosphere. The solution was stirred for 1 h at -78 °C before the addition of 2-isopropoxy-4,4,5,5-tetramethyl-1,3,2-dioxaborolane (1.35 eq per bromine atom). The solution was stirred for an additional time of 15 min at -78°C and the mixture was allowed to warm to room temperature under stirring overnight. The excess of *n*-BuLi was quenched with ethanol and the solvent was removed under reduced pressure. The crude mixture was then dissolved in dichloromethane, washed with water 3 times, dried over Na_2SO_4 and filtered. The residue was purified by flash chromatography on silica gel.

9-phenyl-2,7-bis(4,4,5,5-tetramethyl-1,3,2-dioxaborolan-2-yl)-9H-carbazole (**2a**)

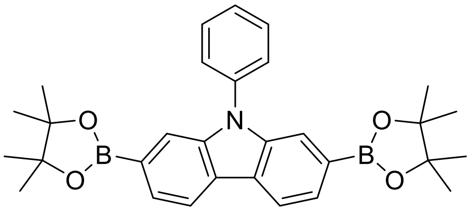

The title compound was synthesized using the general procedure for the borylation reaction with2,7-dibromo-9-phenyl-9H-carbazole (4.00 g, 9.97 mmol, 1.0 eq) in THF (200 mL), *n*-BuLi (2.50 M in hexanes, 10.4 mL, 25.9 mmol, 2.6 eq), 2-isopropoxy-4,4,5,5-tetramethyl-1,3,2-dioxaborolane (6.10 mL, 29.9 mmol, 3.0 eq).

The residue was purified with flash chromatography on silica gel [column conditions: silica cartridge (80 g); solid deposit on Celite®; λdetection: (254 nm, 280 nm); gradient petroleum ether/CH_2Cl_2 from 20% to 60% in 60 min at 60 mL/min], giving the title compound as a colorless powder (3.08 g, **62%**). ^{1}H NMR (300 MHz, CD_2Cl_2) δ 8.17 (dd, *J* = 7.8, 0.8 Hz, 2H), 7.74 (t, *J* = 0.8 Hz, 2H), 7.72 – 7.65 (m, 4H), 7.60 – 7.53 (m, 3H), 1.34 (s, 24H). ^{13}C NMR (75 MHz, CD_2Cl_2) δ 141.13, 137.58, 130.03, 127.78, 127.65, 125.84, 125.34, 119.80, 116.03, 83.79, 24.64. HRMS (ASAP, 190 °C): Found [M+H]+, 496.283, $C_{30}H_{36}NO_4B_2$ required 496.283.

9-(pyridin-2-yl)-2,7-bis(4,4,5,5-tetramethyl-1,3,2-dioxaborolan-2-yl)-9H-carbazole (**2b**)

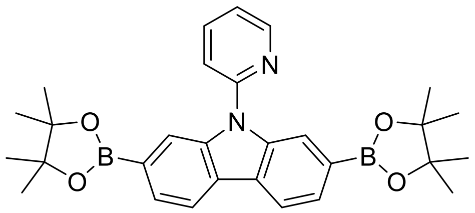

The title compound was synthesized using the general procedure for the borylation reaction with 2,7-dibromo-9-(pyridin-2-yl)-9H-carbazole (3.00 g, 7.46 mmol, 1.0 eq) in THF (100 mL), *n*-BuLi (2.50 M in hexanes, 7.16 mL, 17.9 mmol, 2.4 eq), 2-isopropoxy-4,4,5,5-tetramethyl-1,3,2-dioxaborolane (4.11 mL, 20.1 mmol, 2.7 eq).

After purification with flash chromatography on silica gel [column conditions: silica cartridge (80 g); solid deposit on Celite®; λdetection: (254 nm, 280 nm); gradient petroleum ether/CH_2Cl_2 from 50% to 80% in 60 min at 60 mL/min], the title compound was obtained as a colorless powder (2.60 g, **70%**). ^{1}H NMR (300 MHz, CD_2Cl_2) δ 8.79 (ddd, *J* = 4.9, 2.0, 0.8 Hz, 1H), 8.18 – 8.13 (m, 4H), 8.02 (ddd, *J* = 8.1, 7.5, 2.0 Hz, 1H), 7.75 – 7.65 (m, 3H), 7.39 (ddd, *J* = 7.4, 4.9, 1.0 Hz, 1H), 1.36 (s, 24H). ^{13}C NMR (75 MHz, CD_2Cl_2) δ 151.54, 149.87, 139.75, 138.72, 126.78, 126.18, 121.62, 119.79, 119.72, 117.27, 83.83, 24.67. HRMS (ASAP, 220 °C): Found [M+H]+, 497.278, $C_{29}H_{35}N_2O_4B_2$ required 497.277.

9-(pyrimidin-2-yl)-2,7-bis(4,4,5,5-tetramethyl-1,3,2-dioxaborolan-2-yl)-9H-carbazole (**2c**)

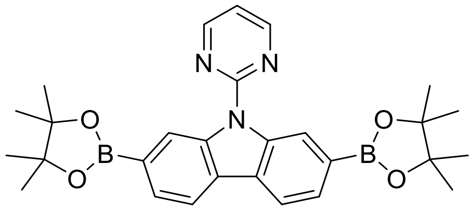

2,7-Dibromo-9-(pyrimidin-2-yl)-9H-carbazole (1.00 g, 2.48 mmol, 1.0 eq), 4,4,4',4',5,5,5',5'-octamethyl-2,2'-bi(1,3,2-dioxaborolane) (1.39 g, 5.46 mmol, 2.2 eq), KOAc (1.46 g, 14.89 mmol, 6.0 eq) and Pd(dppf)$_2$Cl$_2$ (181 mg, 0.25 mmol, 0.10 eq) were dissolved in 1,4-dioxane (20 mL). The reaction mixture was then stirred for 24 h at 80°C under an argon atmosphere. Solvent was then removed under reduced pressure. The crude mixture was dissolved in CH_2Cl_2, washed with a saturated NH_4Cl solution (1 time) and water (2 times), dried over sodium sulfate and filtered. The residue was purified by flash chromatography on silica gel [column conditions: silica cartridge (40 g); solid deposit on Celite®; λdetection: (254 nm, 280 nm); gradient Petroleum ether/CH_2Cl_2 from 30% to 60% in 60 min at 40 mL/min], giving the title compound as a colorless powder (0.84 g, **68%**). ^{1}H NMR (300 MHz, CD_2Cl_2) δ 9.12 (t, *J* = 0.9 Hz, 2H), 8.96 (d, *J* = 4.8 Hz, 2H), 8.12 (dd, *J* = 7.8, 0.8 Hz, 2H), 7.78 (dd, *J* = 7.7, 0.9 Hz, 2H), 7.21 (t, *J* = 4.8 Hz, 1H), 1.40 (s, 24H). ^{13}C NMR (75 MHz, CD_2Cl_2) δ 158.96, 158.22, 139.20, 128.29, 127.74, 121.99, 119.19, 116.41, 83.87, 24.71. HRMS (ASAP, 210 °C): Found [M+H]+, 498.274, $C_{28}H_{34}N_3O_4B_2$ required 498.273.

General procedure for nanohoops synthesis.

Diboronic pinacol ester derivative (1.0 eq), cesium fluoride or potassium phosphate (6.0 eq) and 1,5- cyclooctadieneplatinum (II) dichloride (1.0 eq) were dissolved in 1,2-dichloroethane (40 $mL.mmol^{-1}$ of diboronic derivative) under an argon atmosphere and stirred at 70°C for 24 h or 72 h. Solvent was then removed under reduced pressure and the crude product was dried under vacuum at 60°C overnight. Triphenylphosphine (15 eq) was then added to the crude product and *o*-dichlorobenzene (60 $mL.mmol^{-1}$ of diboronic derivative) was added under an argon atmosphere. The resulting mixture was stirred for one hour at room temperature and then at the reflux (180°C) for 48 h. Then, solvent was removed under reduced pressure. The crude product was dissolved in CH_2Cl_2, washed with water 3 times, dried over Na_2SO_4 and filtered. The residue was purified by flash chromatography on silica gel.

[4]cyclo-*N*-pyridine-2,7-carbazole **[4]C-Py-Cbz**

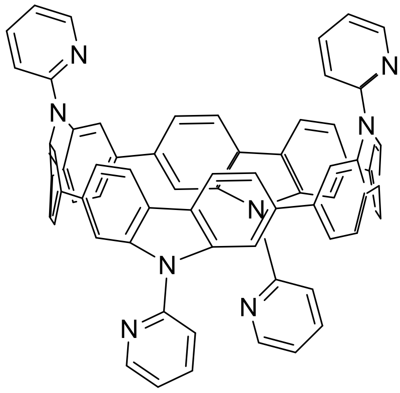

The title compound was synthesized using the general procedure for nanohoops synthesis.

1st step: 9-(pyridin-2-yl)-2,7-bis(4,4,5,5-tetramethyl-1,3,2-dioxaborolan-2-yl)-9H-carbazole (0.250 g, 0.504 mmol, 1.0 eq), potassium carbonate (0.641 g, 3.02 mmol, 6.0 eq) and 1,5-cyclooctadieneplatinum (II) dichloride (0.188 g, 0.504 mmol, 1.0 eq) in extra dry DCE (20 mL) for 72 h.

2nd step: Triphenylphosphine (1.98 g, 7.56 mmol, 15 eq) in 1,2-dichlorobenzene (30 mL). The residue was purified with flash chromatography on silica gel [column conditions: silica cartridge (40 g); solid deposit on Celite®; λdetection (254 nm, 280 nm); gradient dichloromethane/light petroleum 40 % to 100 % for 60 min at 40 mL/min] giving the title compound as a yellow solid (75 mg, **61%**) ^{1}H NMR (300 MHz, CD_2Cl_2) δ 8.43 (ddd, *J* = 4.9, 2.0, 0.8 Hz, 4H), 7.88 (d, *J* = 8.4 Hz, 8H), 7.76 (ddd, *J* = 8.2, 7.4, 2.0 Hz, 4H), 7.52 (dd, *J* = 8.3, 1.6 Hz, 8H), 7.47 – 7.41 (m, 12H), 7.11 (ddd, *J* = 7.4, 4.9, 1.0 Hz, 4H). ^{13}C NMR (75 MHz, CD_2Cl_2) δ 152.12, 148.95, 142.63, 141.35, 137.91, 123.75, 121.83, 119.95, 119.87, 118.71, 117.43. HRMS (MALDI): Found $[M]^+$, 968.336, $C_{68}H_{40}N_8$ required 968.337.

[4]cyclo-*N*-pyrimidine-2,7-carbazole **[4]C-Pm-Cbz**

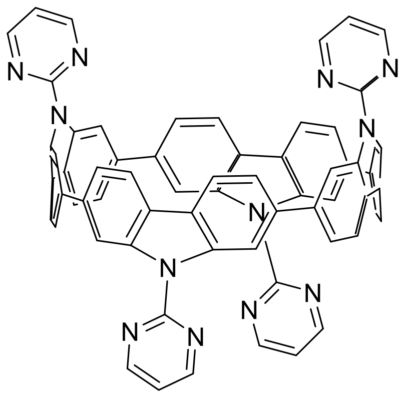

The title compound was synthesized using the general procedure for nanohoops synthesis.

1st step: 9-(pyrimidin-2-yl)-2,7-bis(4,4,5,5-tetramethyl-1,3,2-dioxaborolan-2-yl)-9H-carbazole (0.250 g, 0.503 mmol, 1.0 eq), potassium carbonate (0.640 g, 3.02 mmol, 6.0 eq) and 1,5-cyclooctadieneplatinum (II) dichloride (0.188 g, 0.503 mmol, 1.0 eq) in extra dry DCE (20 mL) for 72 h.

2nd step: Triphenylphosphine (1.98 g, 7.56 mmol, 15 eq) in 1,2-dichlorobenzene (30 mL). The residue was purified with flash chromatography on silica gel [column conditions: silica cartridge (40 g); solid deposit on Celite®; λdetection (254 nm, 280 nm); gradient dichloromethane/light petroleum 30 % to 90 % for 60 min at 40 mL/min] giving the title compound as a yellow solid (65 mg, **53%**). ^{1}H NMR (300 MHz, CD_2Cl_2) δ 8.63 (d, *J* = 4.8 Hz, 8H), 8.08 (dd, *J* = 1.6, 0.5 Hz, 8H), 7.87 (dd, *J* = 8.4, 0.6 Hz,8H), 7.69 (dd, *J* = 8.4, 1.7 Hz, 8H), 6.94 (t, *J* = 4.8 Hz, 4H). ^{13}C NMR (75 MHz, CD_2Cl_2) δ 158.11, 157.76, 141.92, 141.53, 125.07, 123.65, 121.22, 120.07, 115.32. HRMS (MALDI): Found $[M]^+$, 972.320, $C_{68}H_{40}N_8$ required 972.318.

[4]Cyclo-*N*-phenyl-2,7-carbazole **[4]C-Ph-Cbz**

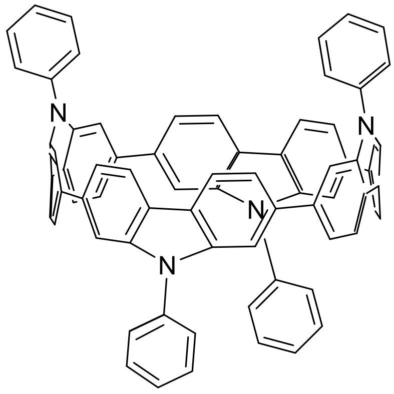

The title compound was synthesized using the general procedure for nanohoops synthesis.

1st step: 9-phenyl-2,7-bis(4,4,5,5-tetramethyl-1,3,2-dioxaborolan-2-yl)-9H-carbazole (0.250 g, 0.505 mmol, 1.0 eq), cesium fluoride (0.460 g, 3.03 mmol, 6.0 eq) and 1,5-cyclooctadieneplatinum (II) dichloride (0.189 g, 0.505 mmol, 1.0 eq) in extra dry DCE (20 mL) for 24 h.

2nd step: Triphenylphosphine (1.99 g, 7.57 mmol, 15 eq) in 1,2-dichlorobenzene (30 mL). The residue was purified first with flash chromatography on silica gel [column conditions: silica cartridge (40 g); solid deposit on Celite®; λdetection (254 nm, 280 nm); gradient dichloromethane/light petroleum 20 % to 70 % for 60 min at 40 mL/min] and then with recycling size exclusion chromatography ($CHCl_3$). Then, the product is recrystallized in DCM/MeOH giving the title compound as a yellow powder (6 mg, **5%**) ^{1}H NMR (300 MHz, CD_2Cl_2) δ 7.85 (d, *J* = 8.4 Hz, 8H), 7.48 – 7.43 (m, 12H), 7.37 (dd, *J* = 8.4, 1.6 Hz, 8H), 7.11 – 7.05 (m, 8H), 6.53 (d, *J* = 1.6 Hz, 8H). ^{13}C NMR (75 MHz, CD_2Cl_2) δ 144.47, 141.19, 137.39, 129.79, 127.58, 127.45, 122.49, 122.16, 117.26, 116.53. HRMS (MALDI): Found $[M]^+$, 964.357, $C_{68}H_{40}N_8$ required 964.356.

Optimization for transmetallation step in nanohoops synthesis

Table S 1 Optimization for transmetallation step in nanohoops synthesis

**Base**	CsF	CsF	K_3PO_4	K_3PO_4	K_3PO_4
**Reaction time**	24 h	72 h	24 h	48 h	72 h
**[4]C-Py-Cbz**	33 %	28 %	3 %	60 %	61 %
**[4]C-Pm-Cbz**	30 %	-	-	-	53 %
**[4]C-Ph-Cbz**	5 %	-	-	-	<1 %

3 Thermal Properties

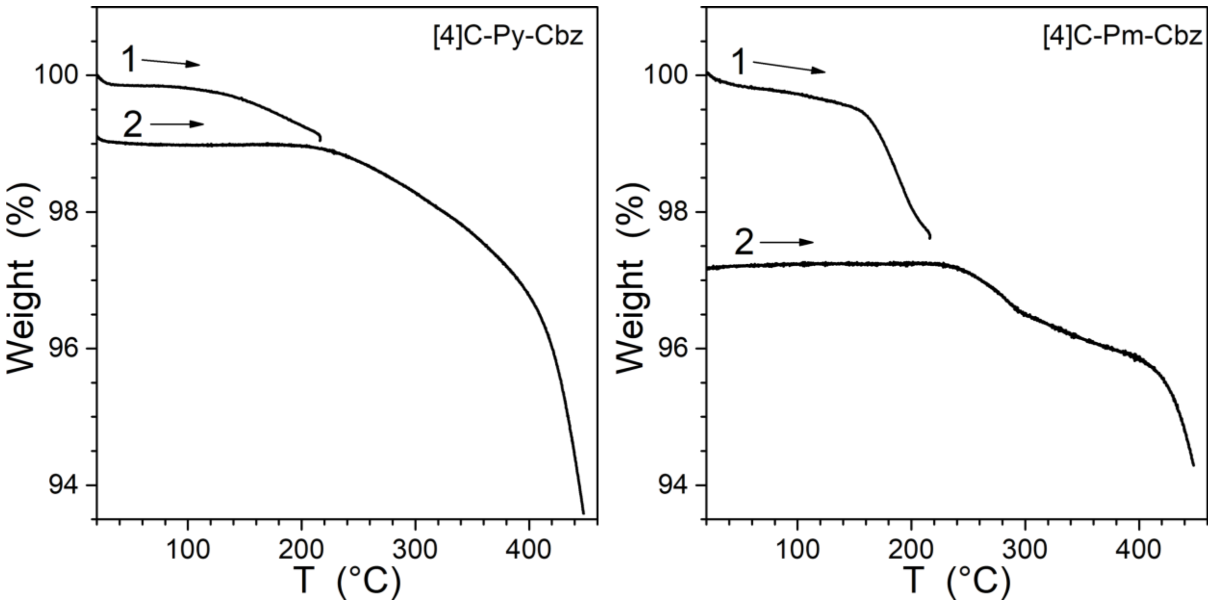

Figure S 1 TGA curves (Q50 from TA Instruments; conditions: 5°C/min, air) of the ***[4]C-Py-Cbz*** *and* ***[4]C-Pm-Cbz****. The nanohoops were heated twice: first heating run to 215°C and isotherm of 2 minutes at 215°C, second heating run to 450°C.*

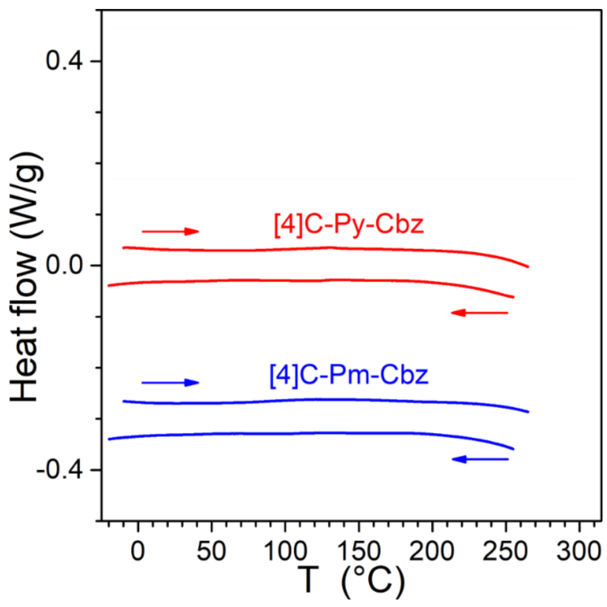

Figure S 2 DSC curves on second heating and cooling (DSC Q1000 from TA Instruments; conditions: 5°C/min, endotherm up)

4 Photophysical properties

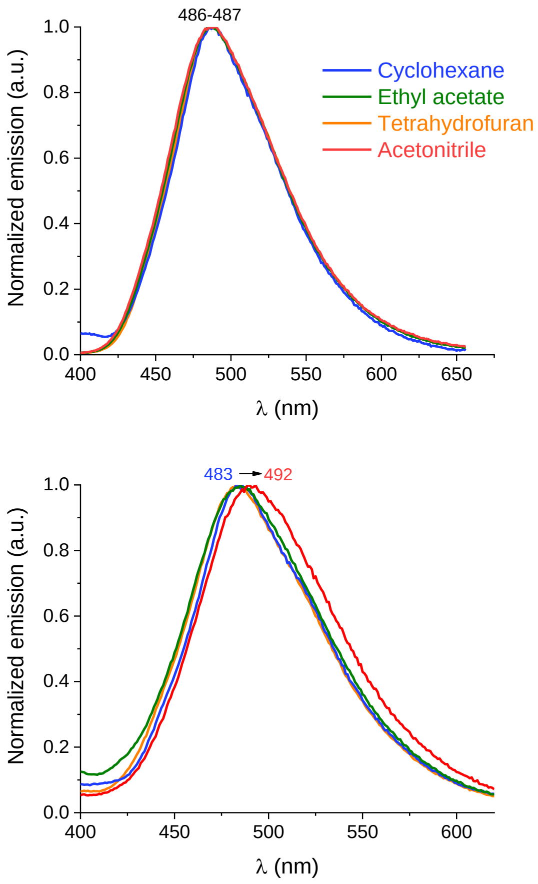

Figure S 3 Emission spectra in various solvent of ***[4]C-Py-Cbz*** *(left) et* ***[4]C-Pm-Cbz*** *(right).*

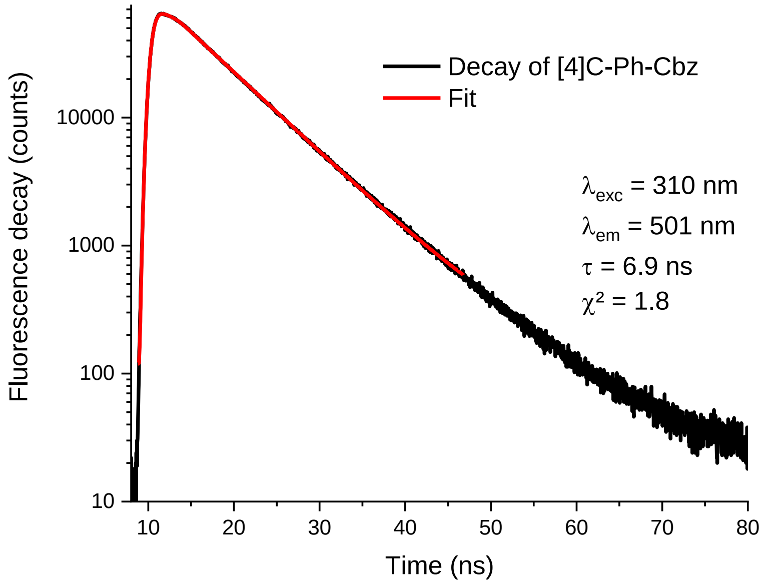

*Figure S 4 Fluorescence decay of **[4]C-Ph-Cbz** in dichloromethane*

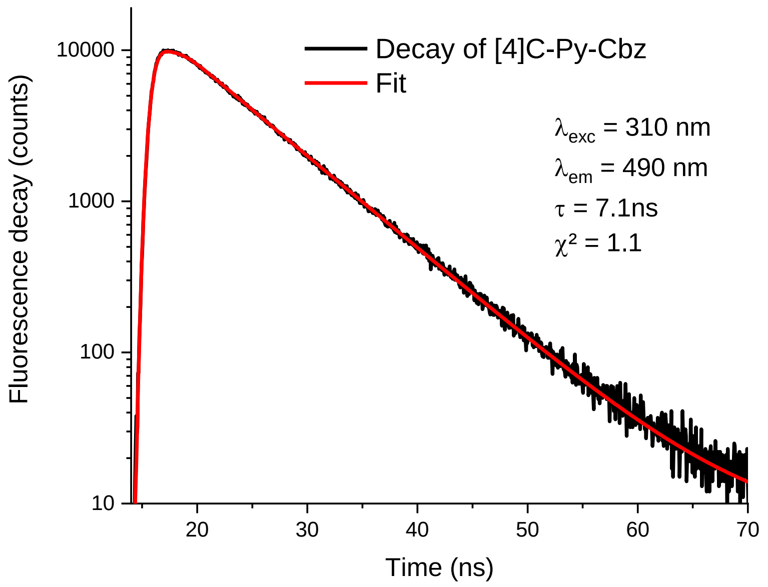

*Figure S 5 Fluorescence decay of **[4]C-Py-Cbz** in dichloromethane*

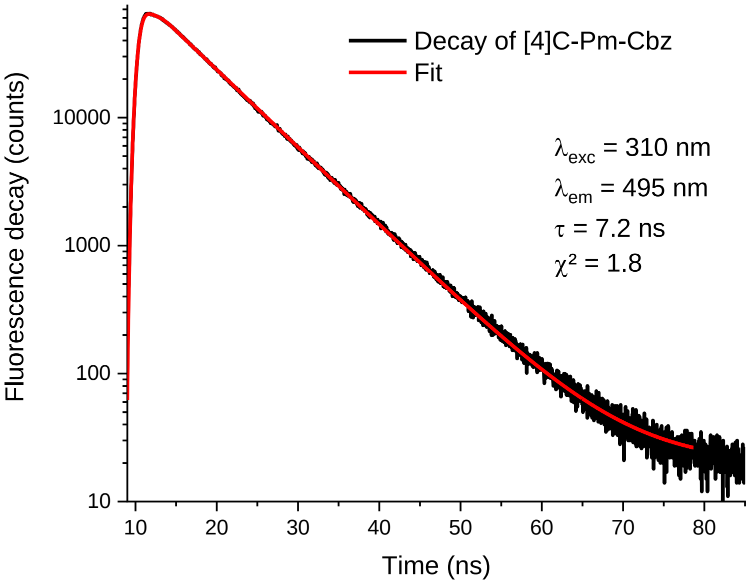

Figure S 6 Fluorescence decay of ***[4]C-Pm-Cbz*** *in dichloromethane*

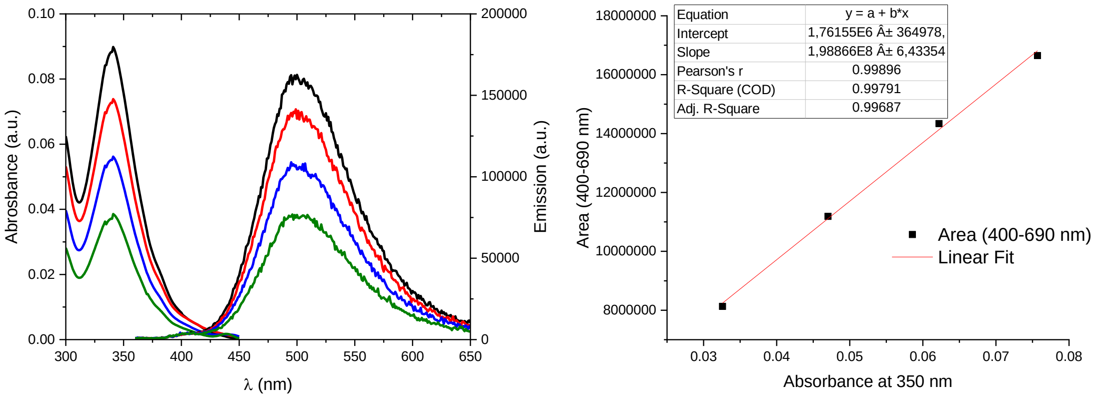

Figure S 7 Absorption and emission spectra of ***[4]C-Ph-Cbz*** *in dichloromethane (left) and linear fit of the integration of the fluorescence between 400 and 690 nm as a function of the absorbance at 350 nm (right).*

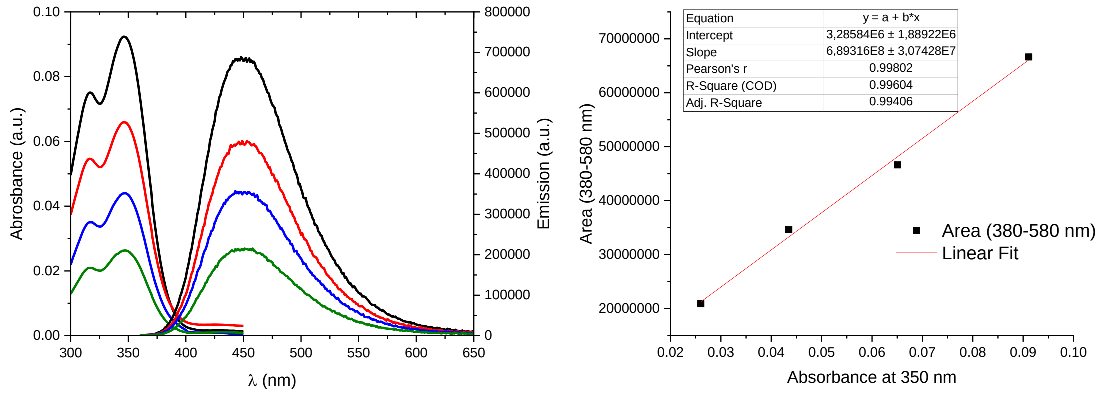

Figure S 8 Absorption and emission spectra of quinine sulfate in 1.0 N sulfuric acid (left) and linear fit of the integration of the fluorescence between 380 and 580 nm as a function of the absorbance at 350 nm (right).

Table S 2 Calculation of the quantum yield of ***[4]C-Ph-Cbz.***

	Quinine sulfate in 1.0 N H_2SO_4	**[4]C-Ph-Cbz** in dichloromethane
Slope	6.89×10^8	1.99×10^8
Refractive index	1.336	1.421
QY	0.546	0.178

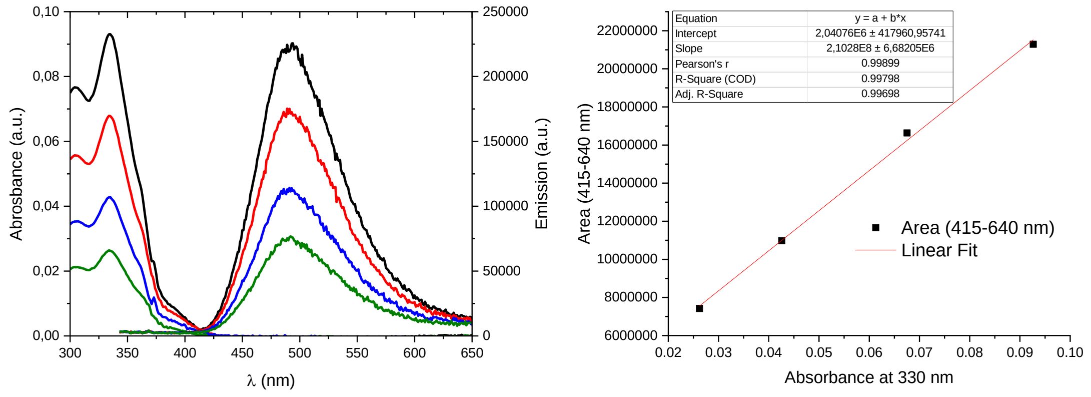

Figure S 9 Absorption and emission spectra of ***[4]C-Py-Cbz*** *in cyclohexane (left) and linear fit of the integration of the fluorescence between 415 and 640 nm as a function of the absorbance at 330 nm (right).*

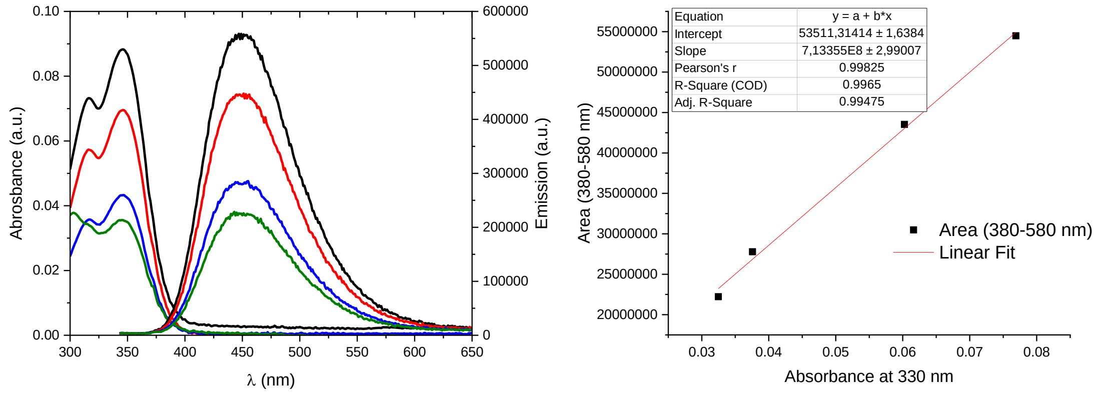

Figure S 10 Absorption and emission spectra of quinine sulfate in 1.0 N sulfuric acid (left) and linear fit of the integration of the fluorescence between 380 and 580 nm as a function of the absorbance at 330 nm (right).

Table S 3 Calculation of the quantum yield of ***[4]C-Py-Cbz****.*

	Quinine sulfate in 1.0 N H_2SO_4	**[4]C-Py-Cbz** in cyclohexane
Slope	7.13×10^{8}	2.10×10^{8}
Refractive index	1.336	1.421
QY	0.546	0.182

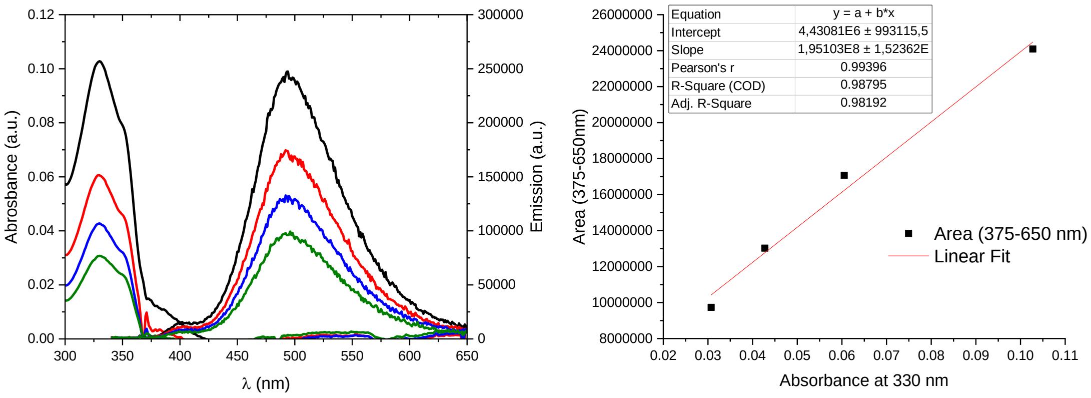

*Figure S 11 Absorption and emission spectra of **[4]C-Pm-Cbz** in cyclohexane (left) and linear fit of the integration of the fluorescence between 375 and 650 nm as a function of the absorbance at 330 nm (right).*

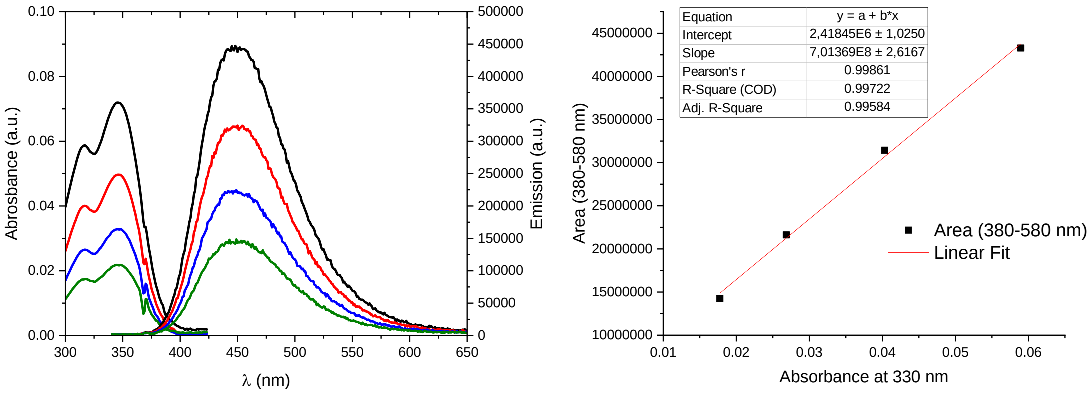

Figure S 12 Absorption and emission spectra of quinine sulfate in 1.0 N sulfuric acid (left) and linear fit of the integration of the fluorescence between 380 and 580 nm as a function of the absorbance at 330 nm (right).

*Table S 4 Calculation of the quantum yield of **[4]C-Pm-Cbz**.*

	Quinine sulfate in 1.0 N H_2SO_4	**[4]C-Pm-Cbz** in dichloromethane
Slope	7.01×10^8	1.95×10^8
Refractive index	1.336	1.421
QY	0.546	0.172

5 Electrochemical properties

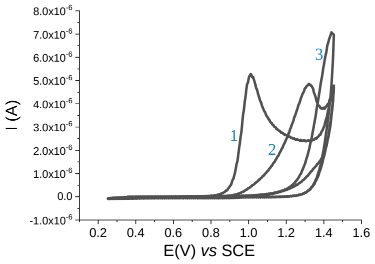

*Figure S 13 Three successive cyclic voltammograms of **[4]C-Pm-Cbz**, recorded between 0.25 and 1.45 V in CH_2Cl_2 + Bu_4NPF_6 0.2 M, platinum disk (diameter 1 mm) working electrode.*

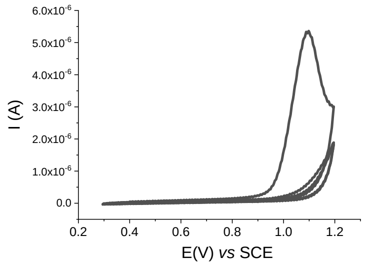

*Figure S 14 Three successive cyclic voltammograms of **[4]C-Py-Cbz**, recorded between 0.30 and 1.20 V in CH_2Cl_2 + Bu_4NPF_6 0.2 M, platinum disk (diameter 1 mm) working electrode.*

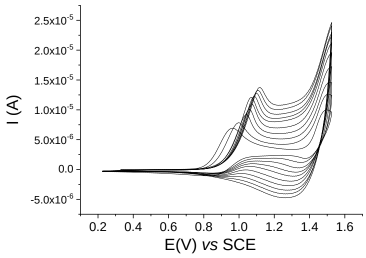

*Figure S 15 Ten successive cyclic voltammograms of **[4]C-Ph-Cbz**, recorded between 0.22 and A.53 V in CH_2Cl_2 + Bu_4NPF_6 0.2 M, platinum disk (diameter 1 mm) working electrode.*

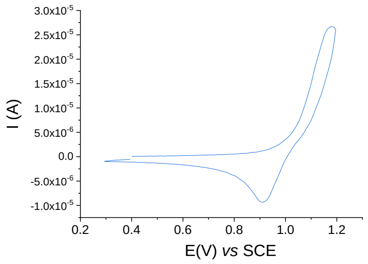

*Figure S 16 Cyclic voltammogram of **[4]C-Py-Cbz**, recorded between 0.29 and 1.20 V in CH_2Cl_2 + Bu_4NPF_6 0.2 M. Sweep-rate of 2 $V.s^{-1}$, platinum disk (diameter 1 mm) working electrode.*

6 Structural properties

Diameter

Table S 5 Diameter values.

	Axes (A)	Φ
**[4]C-Py-Cbz**	11.156	10.896
	10.892	
	10.641	
	10.605	
	10.701	
	10.967	
	11.043	
	11.159	
**[4]C-Pm-Cbz A**	10.78	10.895
	11.011	
	11.118	
	11.116	
	10.993	
	10.797	
	10.696	
	10.649	
**[4]C-Pm-Cbz B**	10.847	10.895
	10.906	
	10.948	
	10.889	
	10.874	
	10.927	
	10.899	
	10.869	

Displacement angle

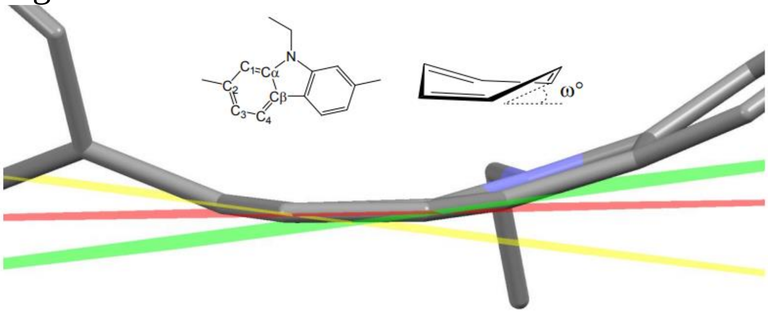

Figure S 17 Measurement of a displacement angle

The displacement angle can represent the deformation of a phenyl unit. Definition of a displacement angle: The mean planes passing by Cα-C1-C3-C4 (red), C1-C2- C3 (yellow) and Cα-Cβ-C4 (green) are drawn. The external displacement angle (ω_{ext}) is measured between red and yellow planes, and, the internal displacement angle (ω_{int}) between red and green planes. Then, the mean displacement angle is calculated considering two ω_{ext} and two ω_{int} for each carbazole building units.

Table S 6 Displacement angle values

	ω_{ext} (°)		ω_{int} (°)		ω (°)
	Values	Average	Values	Average	
**[4]C-Py-Cbz**	9.27	7.23	8.95	6.42	6.83
	7.65		5.96		
	6.33		5.15		
	7.97		6.1		
	7.6		8.02		
	4.11		3.03		
	8.57		8.39		
	6.37		5.76		
**[4]C-Pm-Cbz 1**	6.77	7.41	5.02	6.67	7.04
	7.42		6.57		
	7.89		7.75		
	7.56		7.35		
	6.77		5.02		
	7.42		6.57		
	7.56		7.35		
	7.89		7.75		
**[4]C-Pm-Cbz 2**	6.89	6.76	5.97	6.66	6.71
	7		6.72		
	5.94		6.63		
	7.2		7.33		
	6.89		5.97		
	7		6.72		
	5.94		6.63		
	7.2		7.33		

Torsion angle

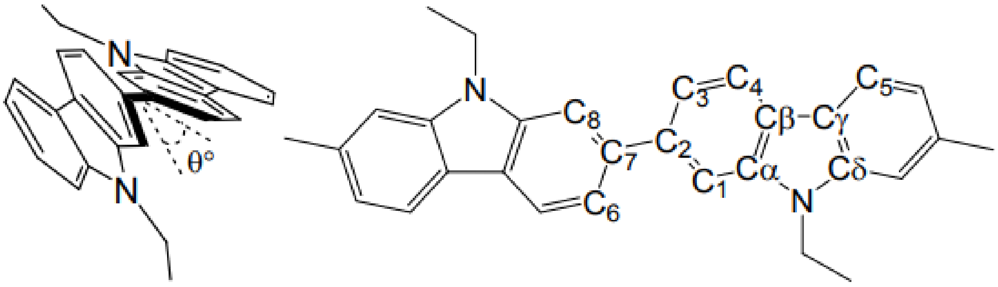

Figure S 18 Definition of the torsion angle between two carbazole units

Definition of a torsion angle: The external torsion angle (θ_{ext}) is the dihedral angle between two carbazole building units. Two angles are measured for each C2-C7 link (C8-C7-C2-C3 and C6-C7-C2-C1). The internal torsion angles (θ_{int}) are the dihedral angles within a building unit (C4-Cβ-Cγ-C5 and Cα-Cβ-Cγ-Cδ).

Table S 7 Torsion angle values

	θ_{ext} (°)		θ_{int} (°)		θ (°)
	Values	Average	Values	Average	
**[4]C-Py-Cbz**	49.15	46.43	0.95	2.31	24.37
	45.32		3.2		
	50.85		1.62		
	51.3		1.86		
	46.02		1.56		
	49.87		5.76		
	39.08		3.52		
	39.81		0.03		
**[4]C-Pm-Cbz A**	44.48	44.70	0.18	0.77	22.73
	44.85		0.39		
	44.52		1.7		
	44.93		0.82		
	44.48		0.18		
	44.85		0.39		
	44.93		1.7		
	44.52		0.82		
**[4]C-Pm-Cbz B**	48.13	46.91	0.02	0.62	23.76
	46.39		2.19		
	47.33		0.1		
	45.77		0.16		
	48.13		0.02		
	46.39		2.19		
	47.33		0.1		
	45.77		0.16		

Dihedral angle between carbazole and substituent

Table S 8 Dihedral angle between carbazole and substituent values

	Angle values (°)	γ
**[4]C-Py-Cbz**	41.98	36.7
	36.03	
	33.3	
	42.12	
	31.41	
	30.95	
	37.71	
	40.08	
**[4]C-Pm-Cbz A**	0.15	1.0
	1.76	
	1.76	
	0.15	
	0.27	
	1.93	
	0.27	
	1.93	
**[4]C-Pm-Cbz B**	7.55	3.0
	1.42	
	7.55	
	1.42	
	1.14	
	1.8	
	1.14	
	1.8	

7 S/WAXS measurement

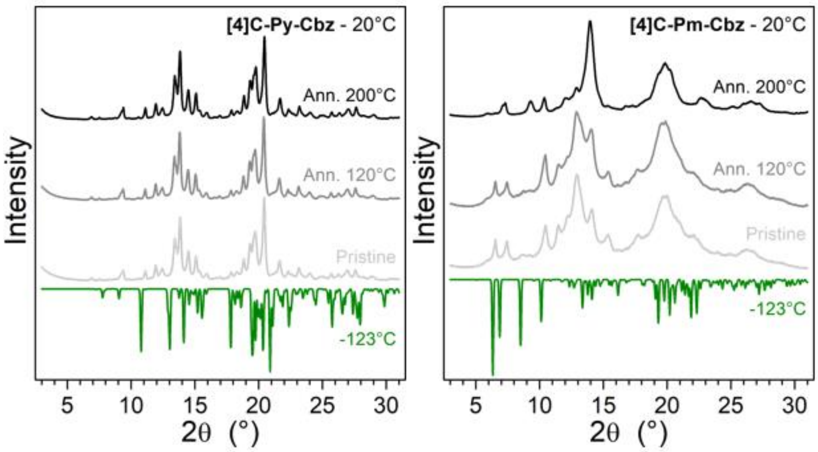

Figure S 19 Small- and wide-angle scattering (S/WAXS) patterns of ***[4]C-Py-Cbz*** *and* ***[4]C-Pm-Cbz*** *powders acquired at room temperature after preheating the sample at increasing annealing temperatures, and comparison to the simulated powder XRD pattern from the single crystal structure of same compounds acquired at -123°C (inverted trace in green).*

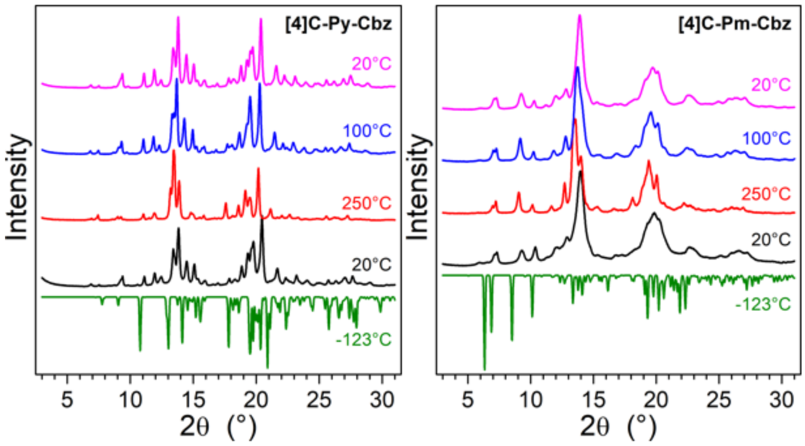

Figure S 20 Small- and wide-angle scattering (S/WAXS) patterns of ***[4]C-Py-Cbz*** *and* ***[4]C-Pm-Cbz*** *powders sequentially acquired at 20°C, 250°C, 100°C and 20°C (black, red, blue and magenta lines), as compared to the simulated powder XRD pattern from the single crystal structure of same compounds acquired at -123°C (green line).*

8 Molecular modelling

Table S 9 Results of TD-DFT calculations for ***[4]C-Ph-Cbz***

λ (nm)	Oscillator Strength	Major contributions	Minor contributions
425	0.0000	HOMO→LUMO (97%)	
358	0.2242	H-1→LUMO (48%),	H-6→LUMO (7%),

		HOMO→L+1 (28%)	H-3→L+2 (4%), HOMO→L+2 (7%)
358	0.2241	H-2→LUMO (48%), HOMO→L+2 (28%)	H-5→LUMO (7%), H-3→L+1 (4%), HOMO→L+1 (7%)
352	0.0000	H-3→LUMO (88%)	H-6→L+2 (3%), H-5→L+1 (3%)
352	0.0710	H-4→LUMO (85%)	H-2→L+1 (4%), H-1→L+2 (3%), HOMO→L+13 (3%)
351	0.0765	H-1→LUMO (32%), HOMO→L+1 (59%)	H-2→LUMO (5%)
351	0.0766	H-2→LUMO (32%), HOMO→L+2 (59%)	H-1→LUMO (5%)
337	0.3826	H-5→LUMO (78%)	H-4→L+1 (5%), H-2→LUMO (8%)
337	0.3829	H-6→LUMO (78%)	H-4→L+2 (5%), H-1→LUMO (8%)
335	0.0000	HOMO→L+3 (92%)	
332	0.0000	HOMO→L+4 (95%)	
329	0.025	HOMO→L+5 (94%)	
329	0.025	HOMO→L+6 (94%)	
323	0.0002	HOMO→L+7 (92%)	
322	0.0000	HOMO→L+8 (97%)	
319	0.0000	H-2→L+1 (24%), H-1→L+2 (24%), HOMO→L+11 (17%)	H-4→LUMO (4%), H-2→L+2 (9%), H-1→L+1 (9%)
318	0.0015	H-2→L+1 (11%), H-2→L+2 (23%), H-1→L+1 (24%), H-1→L+2 (11%)	H-4→LUMO (6%), H-3→L+3 (2%), HOMO→L+7 (5%), HOMO→L+11 (8%)
317	0.0000	H-2→L+2 (40%), H-1→L+1 (40%)	H-2→L+1 (8%), H-1→L+2 (8%)
317	0.0076	HOMO→L+9 (94%)	
317	0.0076	HOMO→L+10 (94%)	

Table S 10 Results of TD-DFT calculations for ***[4]C-Py-Cbz***

λ (nm)	Oscillator Strength	Major contributions	Minor contributions
447	0.0001	HOMO->LUMO (96%)	
372	0.2077	H-1->LUMO (61%), HOMO->L+1 (29%)	
371	0.2085	H-2->LUMO (65%), HOMO->L+2 (25%)	H-5->LUMO (2%)
367	0.0804	H-1->LUMO (29%), HOMO->L+1 (61%)	H-3->LUMO (4%)
365	0.091	H-2->LUMO (26%), HOMO->L+2 (69%)	
360	0.0177	H-6->LUMO (18%), H-3->LUMO (67%)	HOMO->L+1 (6%)
360	0.1799	H-5->LUMO (16%), H-4->LUMO (72%)	
351	0.0007	HOMO->L+3 (82%)	H-6->LUMO (6%), H-3->LUMO (4%), HOMO->L+7 (3%)
350	0.0021	HOMO->L+4 (85%)	H-5->LUMO (6%), H-4->LUMO (2%)
348	0.154	H-6->LUMO (42%), H-3->LUMO (16%), HOMO->L+5 (24%)	HOMO->L+3 (6%), HOMO->L+7 (5%)
347	0.2517	H-5->LUMO (57%), H-4->LUMO (15%), HOMO->L+8 (12%)	HOMO->L+4 (7%)
343	0.1755	H-6->LUMO (21%), HOMO->L+5 (68%)	H-3->LUMO (3%)
341	0.1209	HOMO->L+6 (72%), HOMO->L+8 (13%)	H-5->LUMO (3%), H-4->LUMO (2%), HOMO->L+10 (3%)
340	0.1235	HOMO->L+7 (70%), HOMO->L+9 (13%)	HOMO->L+3 (6%)
336	0.148	HOMO->L+6 (20%), HOMO->L+8 (57%)	H-5->LUMO (7%), HOMO->L+4 (2%), HOMO->L+10 (5%)

334	0.0135	HOMO->L+7 (14%), HOMO->L+9 (75%)	H-2->L+1 (2%)
333	0.0145	HOMO->L+8 (11%), HOMO->L+10 (79%)	H-1->L+1 (3%)
329	0.113	H-2->L+2 (54%), H-1->L+1 (19%)	H-2->LUMO (4%), H-1->L+3 (6%)
328	0.0092	H-2->L+1 (15%), H-1->L+2 (59%)	H-2->L+3 (5%), H-2->L+5 (2%), H-1->LUMO (4%), HOMO->L+7 (3%)
324	0.0043	H-2->L+2 (27%), H-1->L+1 (57%)	H-3->L+1 (8%), HOMO->L+10 (3%)

Table S 11 Results of TD-DFT calculations for ***[4]C-Pm-Cbz***

λ (nm)	Oscillator Strength	Major contributions	Minor contributions
452	0.0000	HOMO→LUMO (97%)	
395	0.0077	HOMO→L+1 (97%)	
395	0.0077	HOMO→L+2 (97%)	
395	0.0000	HOMO→L+3 (97%)	
394	0.0000	HOMO→L+4 (97%)	
369	0.3117	HOMO→L+5 (87%)	HOMO→L+6 (4%)
369	0.3117	HOMO→L+6 (87%)	HOMO→L+5 (4%)
359	0.2057	H-1→LUMO (91%)	H-5→LUMO (2%), HOMO→L+5 (4%)
359	0.2058	H-2→LUMO (91%)	H-6→LUMO (2%), HOMO→L+6 (4%)
354	0.0000	HOMO→L+7 (93%)	
347	0.0000	H-3→LUMO (89%)	
346	0.0074	H-5→LUMO (34%), HOMO→L+9 (29%), HOMO→L+10 (21%)	H-6→LUMO (4%)
346	0.0074	H-6→LUMO (34%), HOMO→L+9 (21%), HOMO→L+10 (29%)	H-5→LUMO (4%)

345	0.0000	HOMO→L+8 (90%)	
345	0.2462	H-4→LUMO (84%)	HOMO→L+12 (3%)
341	0.0000	H-6→L+2 (11%),<br>H-5→L+1 (11%),<br>H-4→L+4 (15%),<br>H-3→L+3 (17%),<br>H-2→L+2 (21%),<br>H-1→L+1 (22%)	
341	0.0000	H-6→L+2 (11%),<br>H-5→L+1 (10%),<br>H-4→L+3 (15%),<br>H-3→L+4 (16%),<br>H-2→L+2 (20%),<br>H-1→L+1 (20%)	H-2→L+1 (2%),<br>H-1→L+2 (2%),<br>HOMO→L+8 (3%)
341	0.0146	H-5→L+3 (11%),<br>H-5→L+4 (11%),<br>H-4→L+1 (16%),<br>H-3→L+1 (16%),<br>H-1→L+3 (21%),<br>H-1→L+4 (17%)	H-6→LUMO (4%)
341	0.0148	H-6→L+3 (11%),<br>H-6→L+4 (11%),<br>H-4→L+2 (16%),<br>H-3→L+2 (16%),<br>H-2→L+3 (21%),<br>H-2→L+4 (18%)	H-5→LUMO (4%)
338	0.3319	H-5→LUMO (42%),<br>HOMO→L+10 (33%)	H-2→L+4 (4%),<br>H-1→LUMO (3%),<br>HOMO→L+9 (6%)

Table S 12 Atomic coordinates of ***[4]C-Ph-Cbz*** *of optimized geometry (DFT B3LYP/6-31g(d))*

C	-4.922112	-0.726907	0.835831
C	-4.55547	-2.056561	1.036416
C	-4.63622	-2.94955	-0.042535
C	-5.291468	-2.535732	-1.227189
C	-5.705313	-1.218494	-1.403616

C	-5.44729	-0.270129	-0.400974
C	-5.346133	1.177973	-0.385806
C	-5.445978	2.173543	-1.37208
C	-4.823404	3.402123	-1.173412
C	-4.095078	3.677395	0.010592
C	-4.160193	2.765516	1.073346
C	-4.765213	1.527524	0.858655
C	-0.7268	4.921381	-0.834454
C	-2.056475	4.554935	-1.035461
C	-2.949768	4.63559	0.043232
C	-2.536168	5.290401	1.228217
C	-1.21895	5.704053	1.405086
C	-0.270324	5.446236	0.402606
C	1.177768	5.344977	0.387876
C	2.173177	5.444763	1.374303
C	3.40192	4.822442	1.175656
C	3.677458	4.094348	-0.008423
C	2.765704	4.15956	-1.071313
C	1.527646	4.764398	-0.856667
C	-3.960596	0.343932	2.904735
C	-2.071182	-0.27549	4.283852
C	-2.679143	0.320107	5.391095
C	-3.925705	0.933767	5.25157
C	0.345194	3.960534	-2.9034
C	-0.273762	2.072341	-4.284416
C	0.323981	2.680345	-5.39045
C	0.938499	3.926336	-5.249379
N	-4.625432	0.376866	1.644724
C	-2.701603	-0.254958	3.039188
C	-4.57039	0.940013	4.0147
N	0.377241	4.624937	-1.643141
C	-0.254571	2.702145	-3.039376
C	0.943467	4.570367	-4.012191
C	4.921797	0.726001	0.835664
C	4.55535	2.055589	1.037151
C	4.635954	2.949252	-0.041254
C	5.29099	2.536142	-1.226274
C	5.704745	1.219004	-1.403579
C	5.446785	0.270003	-0.40151
C	5.345693	-1.178121	-0.387235
C	5.445497	-2.173094	-1.374121
C	4.823101	-3.401858	-1.176095
C	4.095018	-3.677924	0.007874
C	4.160275	-2.76671	1.071184
C	4.76511	-1.52852	0.857139
C	0.726491	-4.920834	-0.837487
C	2.056185	-4.554534	-1.038449
C	2.949664	-4.636077	0.040062
C	2.536135	-5.291576	1.224645

C	1.218857	-5.705217	1.401386
C	0.270137	-5.446527	0.399267
C	-1.177974	-5.345343	0.384685
C	-2.173208	-5.44568	1.371256
C	-3.401999	-4.823364	1.173123
C	-3.677733	-4.094668	-0.01054
C	-2.766205	-4.159366	-1.073639
C	-1.528049	-4.764228	-0.859539
C	3.960999	-0.34643	2.904246
C	2.072901	0.272517	4.285423
C	2.681275	-0.324722	5.391546
C	3.927347	-0.939023	5.250406
C	-0.345523	-3.957595	-2.905234
C	0.273964	-2.067489	-4.283409
C	-0.324067	-2.673588	-5.390361
C	-0.939039	-3.91954	-5.251129
N	4.625083	-0.378333	1.643837
C	2.70243	0.253016	3.04028
C	4.571169	-0.94421	4.013097
N	-0.37758	-4.623873	-1.645952
C	0.254663	-2.699176	-3.039355
C	-0.944114	-4.565482	-4.014917
H	-4.029912	-2.348581	1.937808
H	-5.399596	-3.243039	-2.044963
H	-6.144714	-0.912418	-2.349635
H	-5.924877	1.959081	-2.324166
H	-4.820361	4.134017	-1.976618
H	-3.569402	2.930493	1.96778
H	-2.348375	4.029776	-1.937113
H	-3.243708	5.398477	2.045798
H	-0.913088	6.143249	2.351272
H	1.958542	5.923456	2.326454
H	4.133745	4.81947	1.978925
H	2.930909	3.569024	-1.965882
H	-1.09865	-0.750162	4.382695
H	-2.18464	0.30569	6.358456
H	-4.404383	1.398707	6.109001
H	-0.748982	1.100181	-4.38437
H	0.310612	2.186342	-6.35808
H	1.405092	4.405067	-6.10588
H	-2.22209	-0.696217	2.171368
H	-5.548335	1.397318	3.898442
H	-0.697586	2.222508	-2.172538
H	1.40149	5.547849	-3.894688
H	4.030014	2.347109	1.938817
H	5.399025	3.243962	-2.043617
H	6.144055	0.913514	-2.349829
H	5.924228	-1.958011	-2.326151
H	4.819959	-4.133243	-1.979764

H	3.569602	-2.932254	1.965595
H	2.348069	-4.028852	-1.939805
H	3.243732	-5.400277	2.042094
H	0.913083	-6.145017	2.347319
H	-1.958405	-5.924932	2.323089
H	-4.133707	-4.820839	1.9765
H	-2.931562	-3.568299	-1.967822
H	1.100726	0.747665	4.385486
H	2.18748	-0.311136	6.35928
H	4.406335	-1.405262	6.106958
H	0.749512	-1.095348	-4.381951
H	-0.310586	-2.178089	-6.357225
H	-1.405909	-4.396803	-6.1083
H	2.222527	0.695498	2.173308
H	5.548767	-1.401958	3.895647
H	0.697864	-2.221006	-2.171768
H	-1.402443	-5.542986	-3.898943

Number of imaginary frequency: 0

Table S 13 Atomic coordinates of ***[4]C-Py-Cbz*** *of optimized geometry (DFT B3LYP/6-31g(d))*

C	-1.155188	-4.83956	-0.991292
C	2.403309	-4.386093	-1.101349
C	5.576417	-1.698042	-1.318947
C	1.192242	4.956541	-0.753218
C	-2.362908	4.425993	-1.024459
C	-5.523554	1.757472	-1.471174
C	-5.561641	-1.60685	-1.543236
C	-2.431278	-4.316832	-1.199602
C	1.116682	-4.90546	-0.950801
C	5.044149	-2.974963	-1.170715
C	2.468262	4.417665	-0.92492
C	-5.445751	-0.671825	-0.503051
C	-3.34641	-4.32209	-0.134918
C	0.673822	-5.432754	0.284559
C	4.339546	-3.344516	-0.000126
C	3.33657	4.349562	0.174704
C	-5.412263	0.779902	-0.472015
C	-0.777655	-5.398495	0.255416
C	3.276139	-4.390824	-0.003455
C	5.422382	-0.75902	-0.287512
C	0.756567	5.432636	0.504788
C	-3.274353	4.387208	0.042962
C	-4.456788	-2.399694	0.92537
C	-1.757027	-5.562661	1.245422
C	2.896071	-5.090501	1.166725

C	4.889565	-1.194178	0.948221
C	4.355646	2.3623	1.198531
C	1.693898	5.53406	1.544385
C	-2.944332	5.032858	1.257014
C	-4.911304	1.157494	0.798658
C	-4.976268	-1.115515	0.754558
C	-3.025681	-5.026825	1.048607
C	1.610935	-5.602567	1.315271
C	4.350002	-2.472536	1.098436
C	4.858986	1.078491	0.989651
C	2.967905	5.001535	1.375486
C	-1.670831	5.548371	1.474004
C	-4.39022	2.430813	1.027477
C	-1.081455	4.926916	-0.796044
C	-0.695472	5.421736	0.474484
C	-4.987764	3.022541	-1.253826
C	-4.336827	3.341788	-0.039746
C	-5.048744	-2.88897	-1.375171
C	-4.40244	-3.268387	-0.174668
C	5.07288	2.946424	-1.050116
C	4.373922	3.277477	0.133987
C	5.588854	1.669717	-1.24777
C	5.410718	0.692793	-0.25777
N	0.003636	-4.681868	-1.786257
N	-4.794168	-0.002173	1.601635
N	4.684325	-0.078115	1.784449
N	0.07575	4.79196	-1.599679
C	-4.579347	-0.063531	2.988146
C	0.065424	-4.386067	-3.159445
C	0.133037	4.576714	-2.986316
C	4.390237	-0.135754	3.158116
N	-0.89463	3.93508	-3.553953
C	-0.861192	3.733706	-4.876061
C	0.186561	4.136877	-5.698242
C	1.24792	4.822259	-5.101292
C	1.225769	5.059938	-3.733874
N	-0.94177	-3.679437	-3.683485
C	-0.903279	-3.399593	-4.991239
C	0.130934	-3.785723	-5.838547
C	1.170349	-4.539288	-5.287404
C	1.14133	-4.858425	-3.936486
N	-3.922621	0.954205	3.556304
C	-3.721597	0.916956	4.878395
C	-4.140041	-0.12526	5.699938
C	-4.841017	-1.176087	5.102404
C	-5.078498	-1.149581	3.735058
N	3.702893	0.883615	3.684333
C	3.425484	0.849571	4.992714

C	3.795282	-0.191605	5.838766
C	4.528974	-1.243965	5.285393
C	4.845284	-1.22017	3.933712
H	2.65425	-3.802491	-1.979489
H	6.023611	-1.405555	-2.265424
H	-2.567038	3.899351	-1.944523
H	-5.942887	1.505377	-2.44182
H	-5.958958	-1.306523	-2.509309
H	-2.627478	-3.74428	-2.093623
H	5.088572	-3.671076	-2.003885
H	2.709272	3.870483	-1.828977
H	-3.913072	-2.649452	1.829164
H	-1.505738	-6.0279	2.195118
H	3.591893	-5.146844	1.999421
H	3.786902	2.56695	2.093078
H	1.40042	5.936239	2.510553
H	-3.669261	5.043493	2.066245
H	-3.755441	-5.075231	1.852416
H	1.312045	-6.045968	2.261527
H	3.76323	-2.714765	1.976893
H	3.659718	4.99745	2.213106
H	-1.411797	5.963545	2.444605
H	-3.860472	2.626382	1.947601
H	-4.986782	3.747792	-2.062838
H	-5.055085	-3.580136	-2.213316
H	5.132091	3.675714	-1.853625
H	6.049166	1.411475	-2.197993
H	-1.722855	3.214301	-5.292338
H	0.165627	3.934674	-6.764191
H	2.078961	5.187193	-5.698992
H	2.013954	5.626727	-3.253342
H	-1.747891	-2.828567	-5.373034
H	0.115983	-3.518483	-6.89014
H	1.988916	-4.893209	-5.908369
H	1.911409	-5.47763	-3.492417
H	-3.189499	1.770607	5.295142
H	-3.937423	-0.107992	6.765875
H	-5.218029	-2.002044	5.699651
H	-5.656852	-1.929024	3.254026
H	2.870435	1.704039	5.376147
H	3.53094	-0.172311	6.891019
H	4.869881	-2.06891	5.905198
H	5.449818	-2.000928	3.488124

Number of imaginary frequency: 0

Table S 14 Atomic coordinates of ***[4]C-Pm-Cbz*** *of optimized geometry (DFT B3LYP/6-31g(d))*

C	0.494131	5.015975	0.905654
C	-0.886387	4.933114	1.079692
H	-1.287272	4.513249	1.989512
C	-1.728319	5.190745	-0.014632
C	-1.171246	5.718671	-1.20097
H	-1.827229	5.970934	-2.029607
C	0.207163	5.811907	-1.366627
H	0.618537	6.13351	-2.319829
C	1.055994	5.372385	-0.341516
C	2.432365	4.905344	-0.341745
C	3.372972	4.737295	-1.36722
H	3.241935	5.242763	-2.320418
C	4.409824	3.824178	-1.201975
H	5.083434	3.624893	-2.030906
C	4.530942	3.066361	-0.015596
C	3.706746	3.374749	1.079106
H	3.769732	2.797803	1.98902
C	2.66172	4.280685	0.905387
C	5.016082	-0.494132	-0.905634
C	4.933213	0.886389	-1.079659
H	4.513381	1.287286	-1.989489
C	5.190812	1.728315	0.014676
C	5.718727	1.171239	1.20102
H	5.970968	1.827216	2.029668
C	5.811968	-0.20717	1.366665
H	6.133562	-0.618551	2.319868
C	5.372467	-1.055998	0.341539
C	4.905387	-2.432349	0.34177
C	4.737324	-3.372944	1.367253
H	5.242794	-3.241905	2.32045
C	3.82419	-4.40978	1.202018
H	3.624891	-5.083379	2.030955
C	3.066372	-4.530891	0.01564
C	3.374774	-3.706712	-1.079072
H	2.797828	-3.769701	-1.988983
C	4.280731	-2.661695	-0.905368
C	1.451745	4.275377	3.103839
C	0.248258	4.394166	5.031146
H	-0.696306	4.640446	5.51436
C	1.332609	3.922554	5.767864
H	1.285396	3.78273	6.841679
C	2.480547	3.639238	5.030991
H	3.380777	3.261663	5.51408
C	4.275648	-1.451733	-3.103876
C	4.394732	-0.248229	-5.031167
H	4.641215	0.696297	-5.514352

C	3.922979	-1.332486	-5.767935
H	3.783242	-1.285237	-6.841758
C	3.639404	-2.480376	-5.031091
H	3.261696	-3.380532	-5.514214
N	1.52307	4.486319	1.732803
N	0.290143	4.575074	3.711565
N	2.556883	3.808474	3.711412
N	4.486479	-1.523078	-1.732816
N	4.575574	-0.290177	-3.711579
N	3.808555	-2.556761	-3.711501
C	-0.494132	-5.015976	0.905654
C	0.886386	-4.933114	1.079694
H	1.287269	-4.513249	1.989514
C	1.728318	-5.190745	-0.01463
C	1.171247	-5.718672	-1.200968
H	1.82723	-5.970934	-2.029605
C	-0.207162	-5.811907	-1.366626
H	-0.618536	-6.13351	-2.319829
C	-1.055995	-5.372385	-0.341516
C	-2.432366	-4.905344	-0.341747
C	-3.372972	-4.737295	-1.367222
H	-3.241934	-5.242763	-2.320421
C	-4.409824	-3.824178	-1.201978
H	-5.083433	-3.624893	-2.03091
C	-4.530943	-3.066362	-0.0156
C	-3.706748	-3.374749	1.079103
H	-3.769734	-2.797803	1.989017
C	-2.661721	-4.280684	0.905385
C	-5.016082	0.494132	-0.905638
C	-4.933213	-0.886389	-1.079663
H	-4.51338	-1.287286	-1.989493
C	-5.190812	-1.728315	0.014672
C	-5.718728	-1.171239	1.201015
H	-5.970971	-1.827216	2.029663
C	-5.811969	0.20717	1.36666
H	-6.133565	0.618551	2.319863
C	-5.372468	1.055997	0.341535
C	-4.905388	2.432349	0.341766
C	-4.737326	3.372944	1.367248
H	-5.242796	3.241905	2.320445
C	-3.824191	4.40978	1.202014
H	-3.624893	5.08338	2.030951
C	-3.066373	4.530891	0.015636
C	-3.374774	3.706711	-1.079075
H	-2.797827	3.7697	-1.988986
C	-4.280731	2.661695	-0.905372
C	-1.451749	-4.275377	3.103838
C	-0.248264	-4.394166	5.031147

H	0.696299	-4.640447	5.514362
C	-1.332616	-3.922553	5.767864
H	-1.285403	-3.782728	6.841678
C	-2.480552	-3.639235	5.030988
H	-3.380782	-3.261659	5.514077
C	-4.275643	1.451732	-3.103879
C	-4.394723	0.248228	-5.03117
H	-4.641204	-0.696298	-5.514356
C	-3.922969	1.332485	-5.767936
H	-3.78323	1.285237	-6.84176
C	-3.639396	2.480376	-5.031092
H	-3.261687	3.380532	-5.514215
N	-1.523072	-4.486319	1.732802
N	-0.290148	-4.575075	3.711566
N	-2.556886	-3.808472	3.71141
N	-4.486477	1.523077	-1.732819
N	-4.575568	0.290176	-3.711582
N	-3.808549	2.556761	-3.711503

Number of imaginary frequency: 0

9 Organic field effect transistor measurements

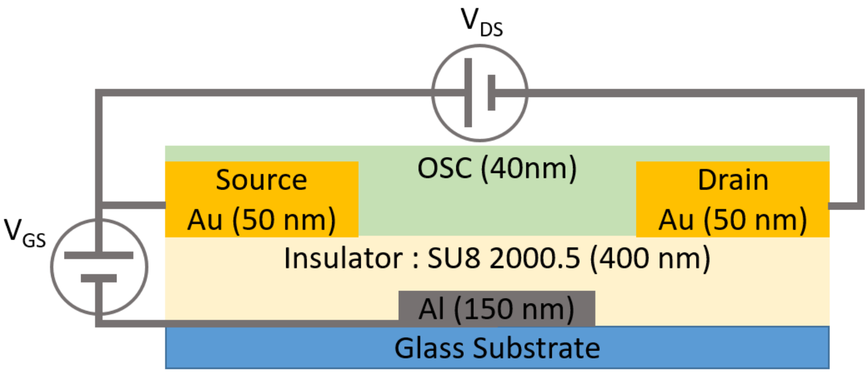

Figure S 21 Structure of the bottom-gate bottom-contact p-type channel OFETs on glass substrate.

OFET: Gate Bias Stress measurement

A gate bias stress is employed herein to estimate the electrical stability of **[4]C-Py-Cbz** and **[4]C-Pm-Cbz** based OFETs. The threshold voltage shift was evaluated by measuring transfer characteristics (I_D vs. V_{GS}) from V_{GS}=10 V to V_{GS}=-60 V every 10 minutes. Between two measurements, constant voltages were applied on gate electrode (V_{GS}=-40V) and on drain electrode (V_{DS}= -10 V).

The Stretched exponential model has been applied on ΔV_{TH} to evaluate the structural trap effect on the electrical stability of OFETs according to the following relationship.

$$V_{TH}(t) - V_{TH}(t=0) = \Delta V_{TH}\max\left(1 - e^{-\left(\frac{t}{\tau}\right)^{\beta}}\right)$$

Where $\Delta V_{TH}max$ is the maximum threshold voltage shift under unlimited stress time, $V_{TH}(t=0)$ is the initial threshold voltage, $V_{TH}(t)$ is threshold voltage at the stress time t. β is related to width of the involved trap distribution and τ is related to trapping time.

10 AFM

Atomic force microscopy measurement

AFM images were recorded on the Bruker Multimode 8 using PeakForce Tapping with a resolution of 512 × 512 pixels on a 500 nm × 500 nm surface. Scan rate has been set at 1Hz. Each scan line in the image was scanned from left to right (trace) and from right to left (retrace). The observed topographic features were verified for their consistency between trace and retrace images. Amplitude setpoint was set to 1.68mV and the drive amplitude was set to 10.38mV. Roughness has been extracted from these images using Nano Scope Analysis 1.8 after a clean image treatment.

*Table S13 Roughness parameters extracted from AFM images (500×500nm²) of **[4]C-Py-Cbz** and **[4]C-Pm-Cbz***

	R_a (Arithmetic Average Rougness) [nm]	R_q (RMS Rougness) [nm]	R_{max} (Maximum Rougness depth) [nm]
**[4]C-Py-Cbz**	0.77	0.98	8.65
**[4]C-Pm-Cbz**	0.72	0.91	6.86

11 X-ray diffraction structures and tables

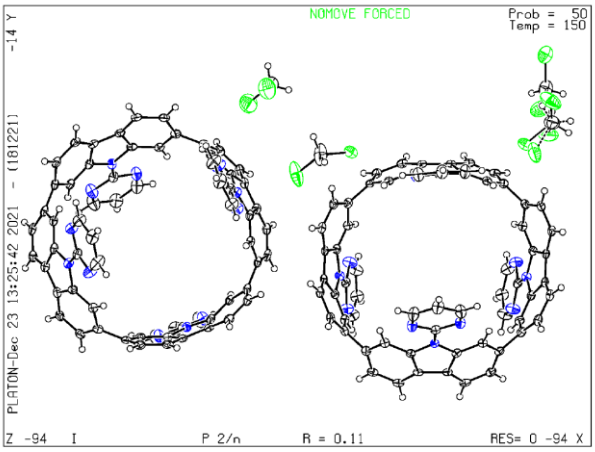

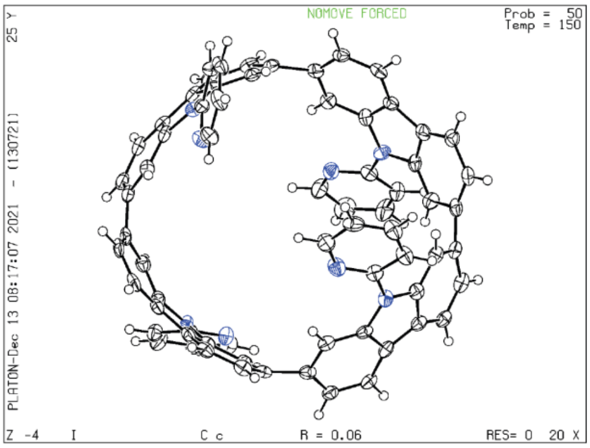

Table S 15 Crystal data and structure refinement for **[4]C-Pm-Cbz** and **[4]C-Py-Cbz**

	[4]C-Pm-Cbz	***[4]C-Py-Cbz***
Chemical formula	$C_{64}H_{36}N_{12}\cdot4(CH_2Cl_2)$	$C_{34}H_{20}N_4$
M_r	1312.75	484.54
Crystal system, space group	Monoclinic, *P*2/*n*	Monoclinic, *Cc*
Temperature (K)	150	150
a, *b*, *c* (Å)	26.088 (6), 9.185 (2), 28.301 (6)	13.7773 (13), 24.331 (2), 17.5236 (16)
β (°)	99.909 (7)	111.036 (4)
V ($Å^3$)	6680 (3)	5482.7 (9)
Z	4	8
Radiation type	Mo *K*α	Mo *K*α
μ (mm^{-1})	0.39	0.07
Crystal size (mm)	0.13 × 0.03 × 0.02	0.09 × 0.04 × 0.02
Absorption correction	Multi-scan	Multi-scan
T_{min}, T_{max}	0.986, 0.992	0.997, 0.999
No. of measured, independent and observed [$I > 2\sigma(I)$] reflections	278509, 15374, 11177	21229, 11588, 9338
R_{int}	0.156	0.047
$R[F^2 > 2\sigma(F^2)]$, $wR(F^2)$, S	0.105, 0.280, 1.05	0.061, 0.152, 1.03
No. of reflections	15374	11588
No. of parameters	812	688
$\Delta\rangle_{max}$, $\Delta\rangle_{min}$ (e $Å^{-3}$)	3.02, -1.37	0.79, -0.23

12 Copy of NMR Spectra

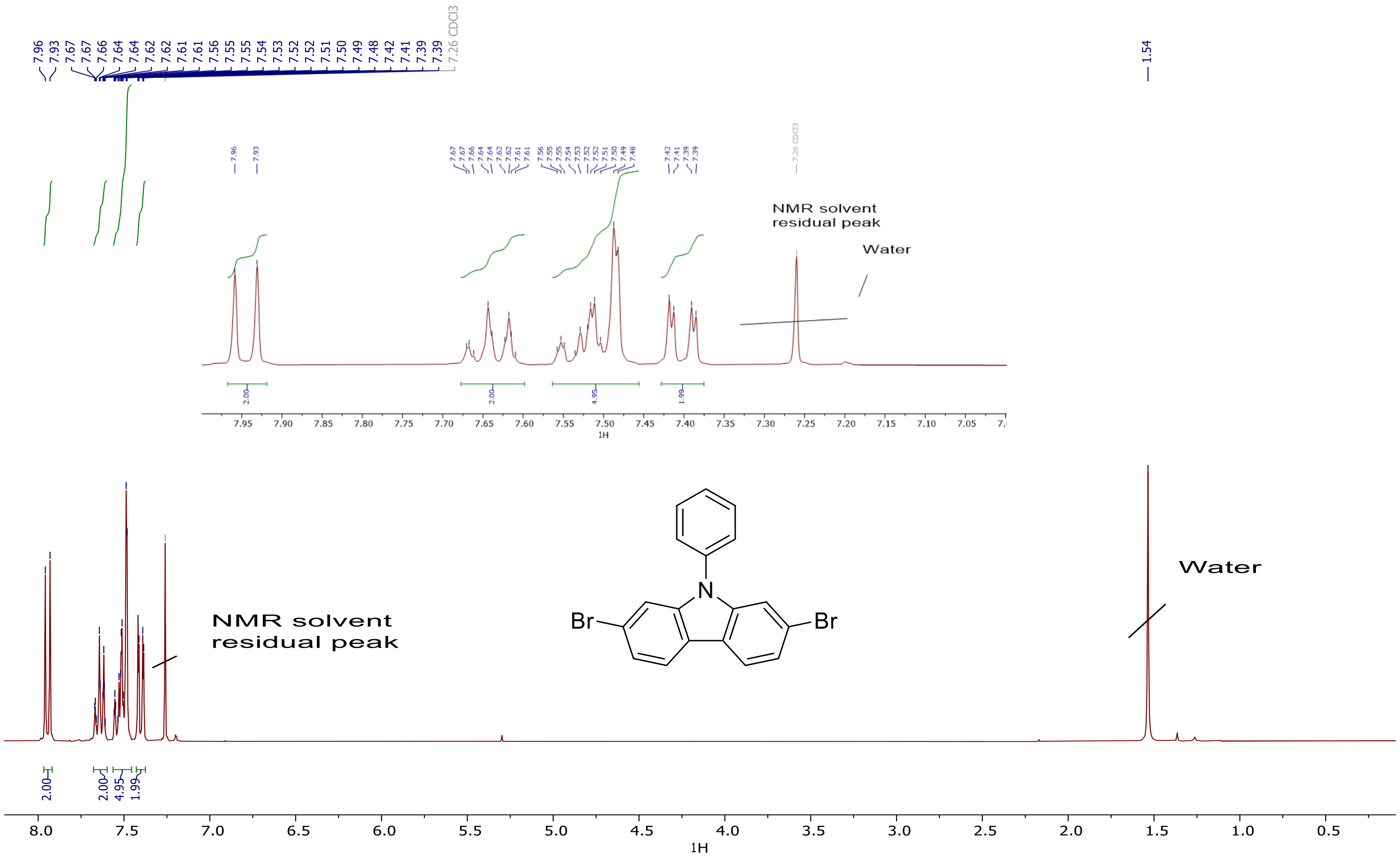

*Figure S 22 ^{1}H-NMR of **1a** in $CDCl_3$*

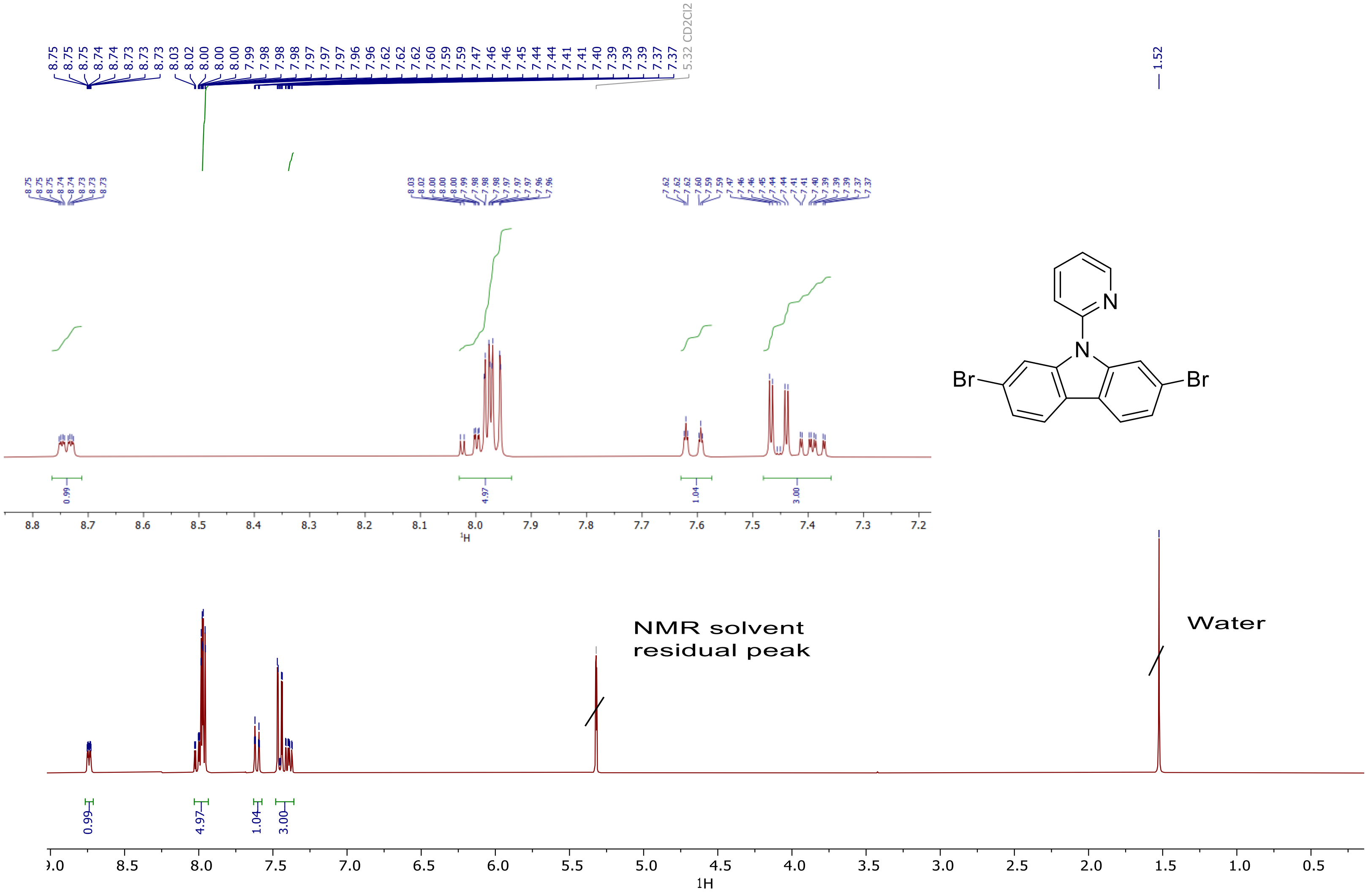

Figure S 23 ^{1}H-NMR of ***1b*** *in* CD_2Cl_2

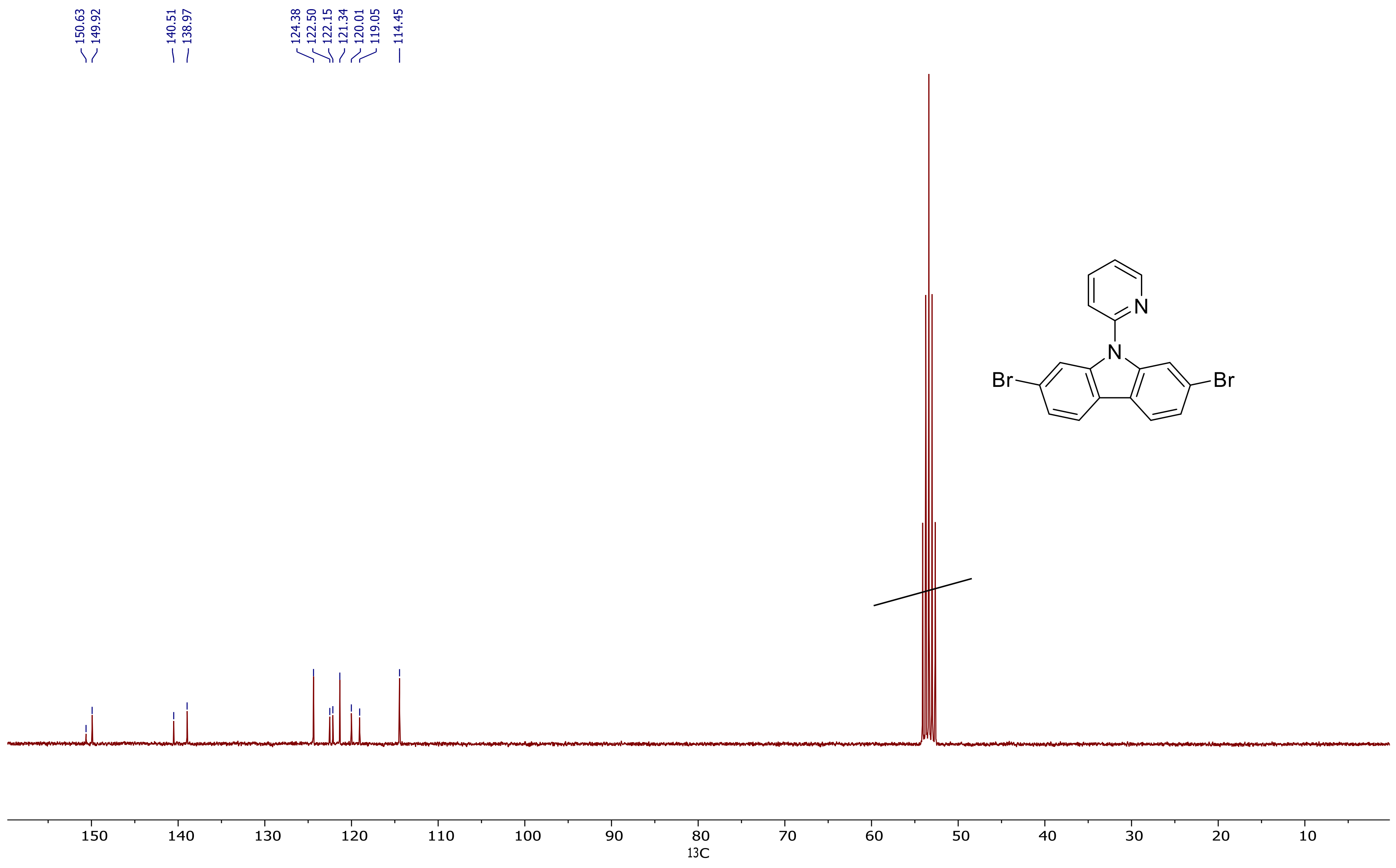

*Figure S 24 ^{13}C-NMR of **1b** in CD_2Cl_2*

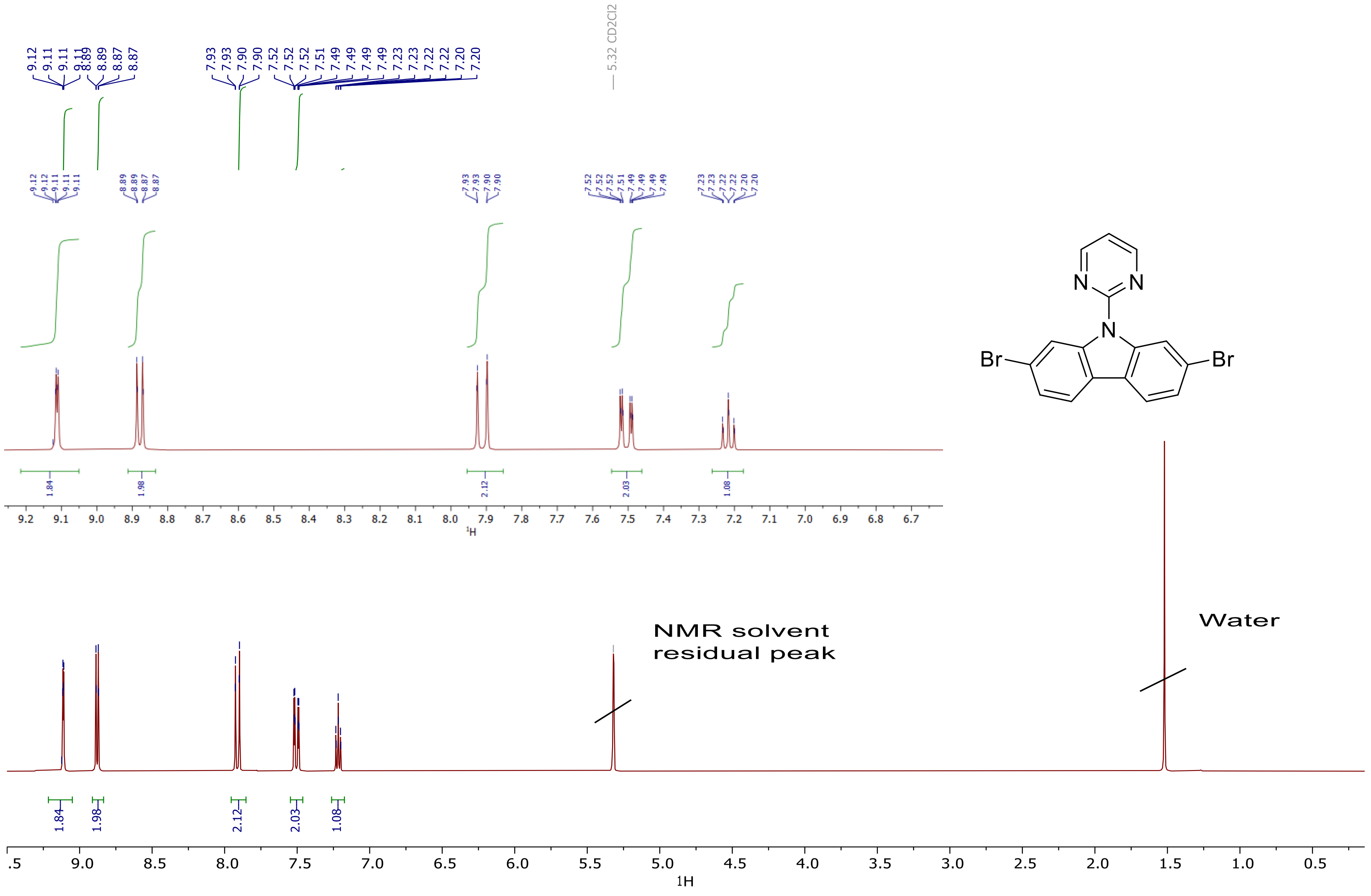

*Figure S 25 ^{1}H-NMR of **1c** in CD_2Cl_2*

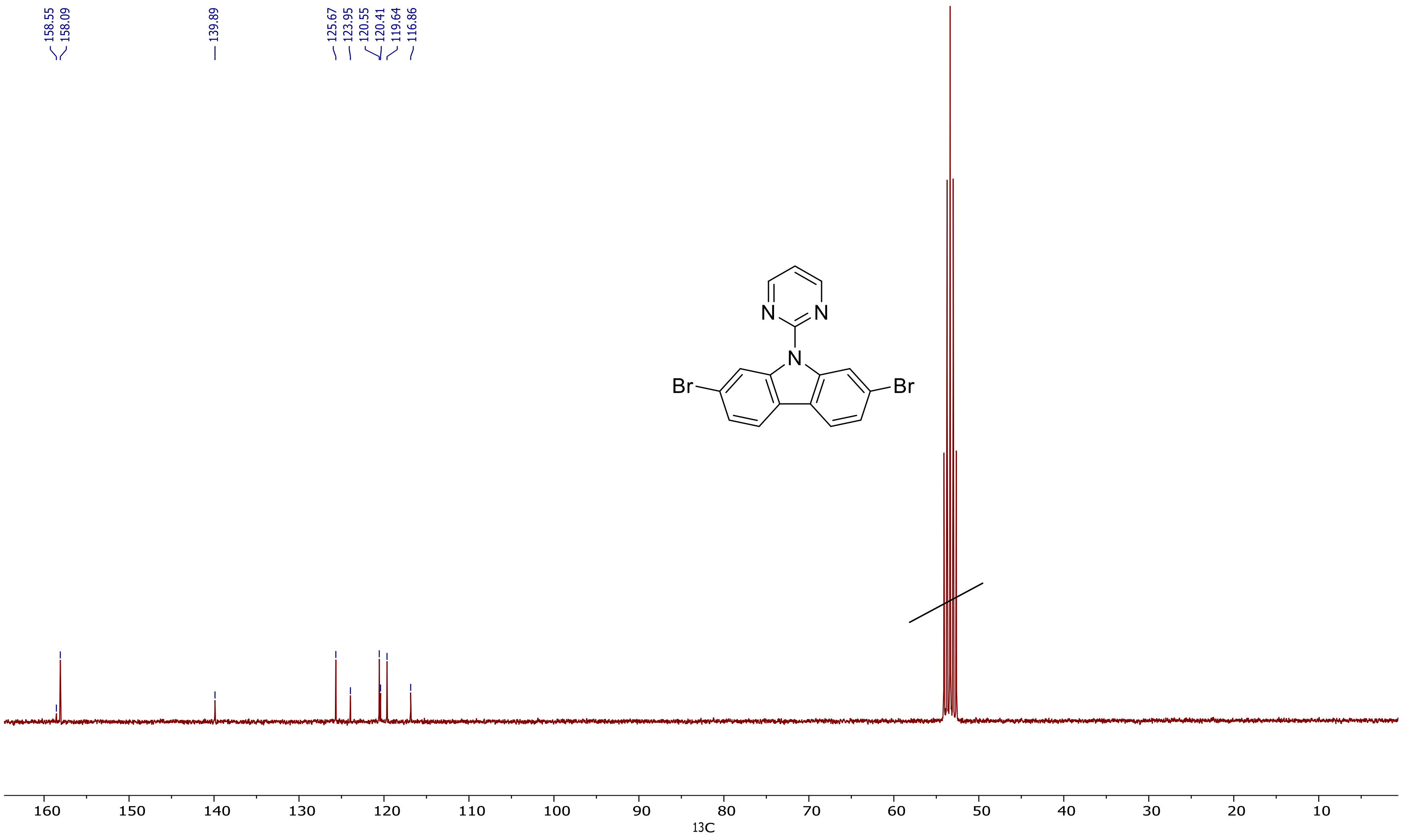

*Figure S 26 ^{13}C-NMR of **1c** in CD_2Cl_2*

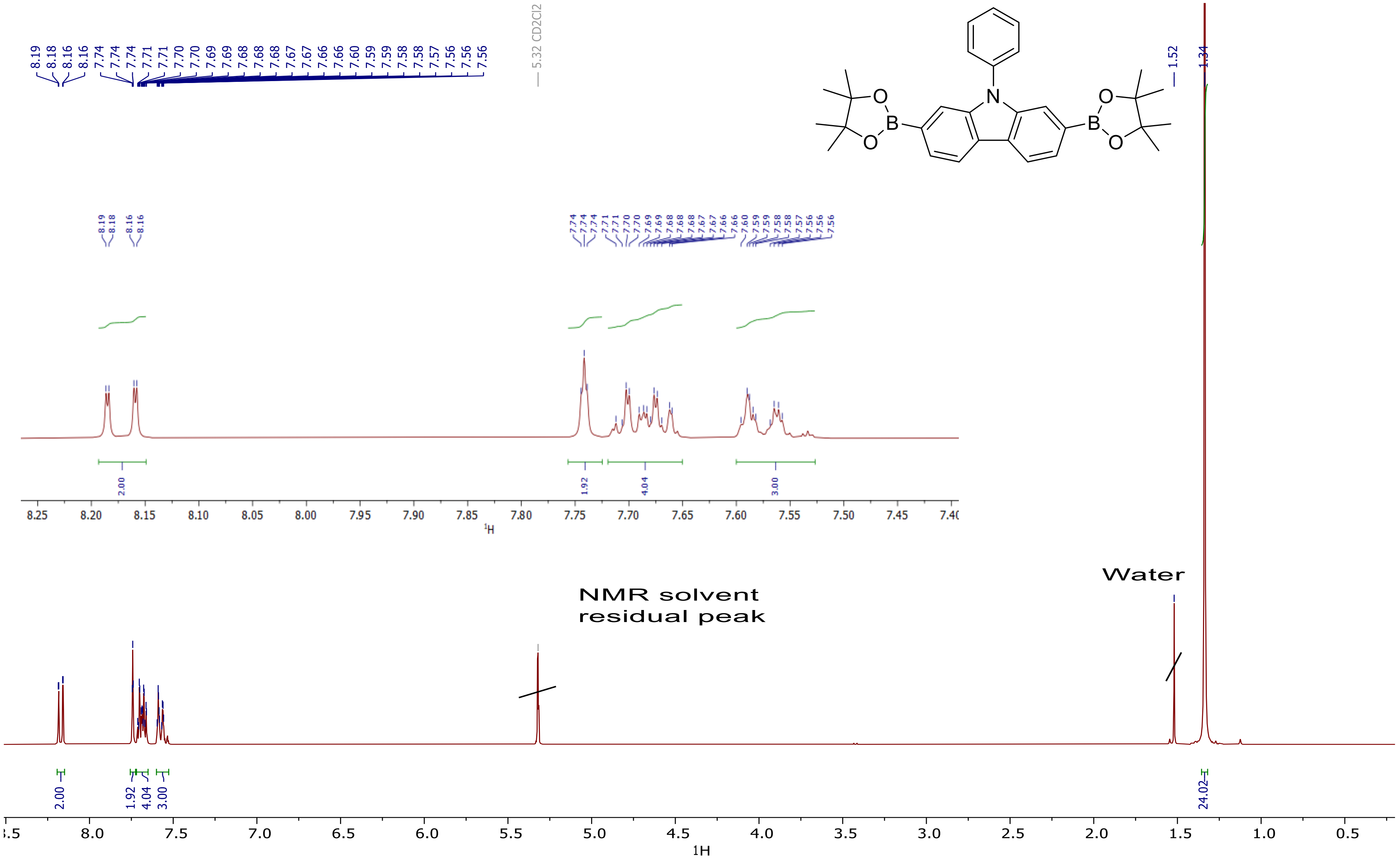

Figure S 27 [1]*H-NMR of* ***2a*** *in* CD_2Cl_2

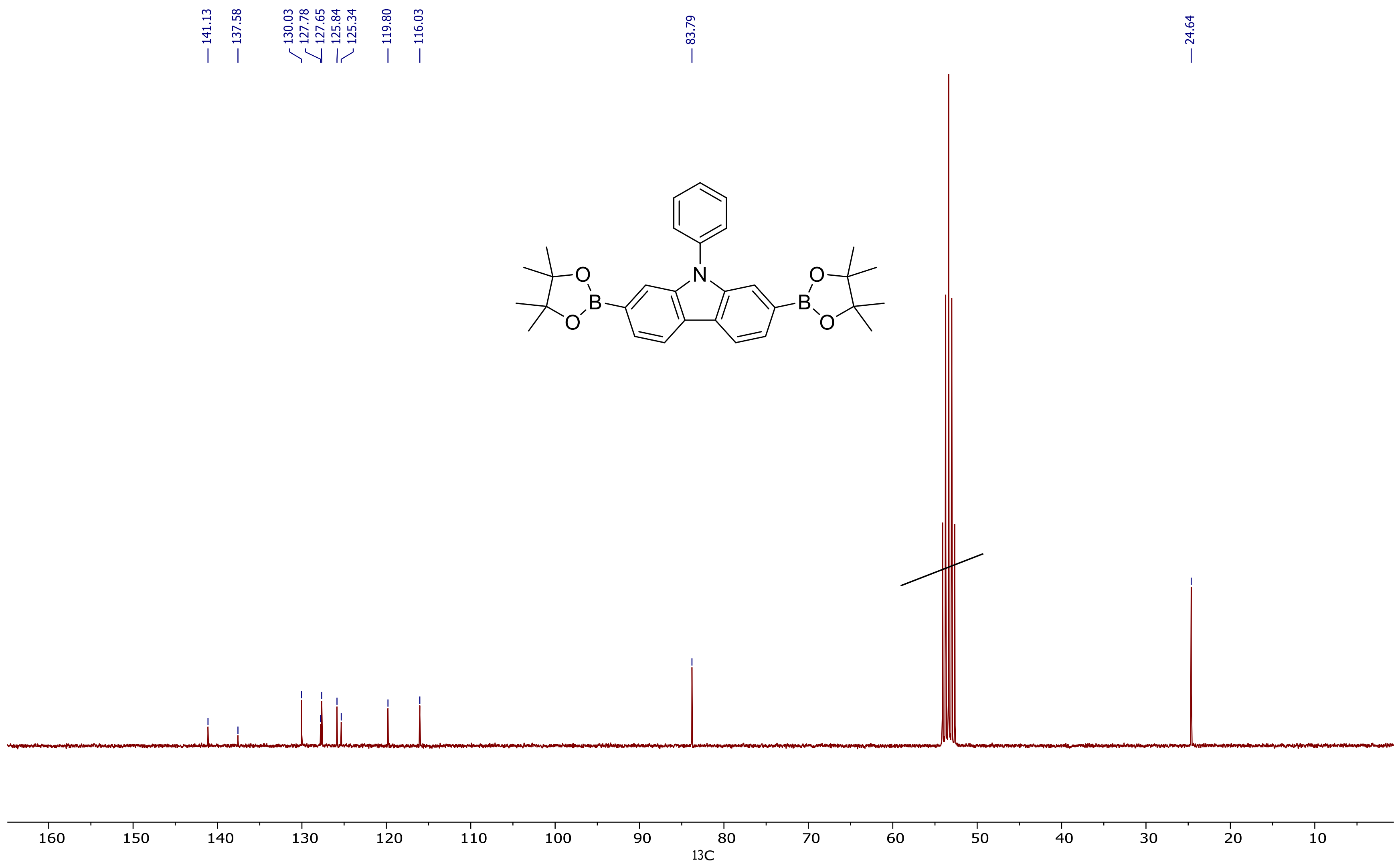

Figure S 28 ^{13}*C-NMR of* ***2a*** *in* CD_2Cl_2

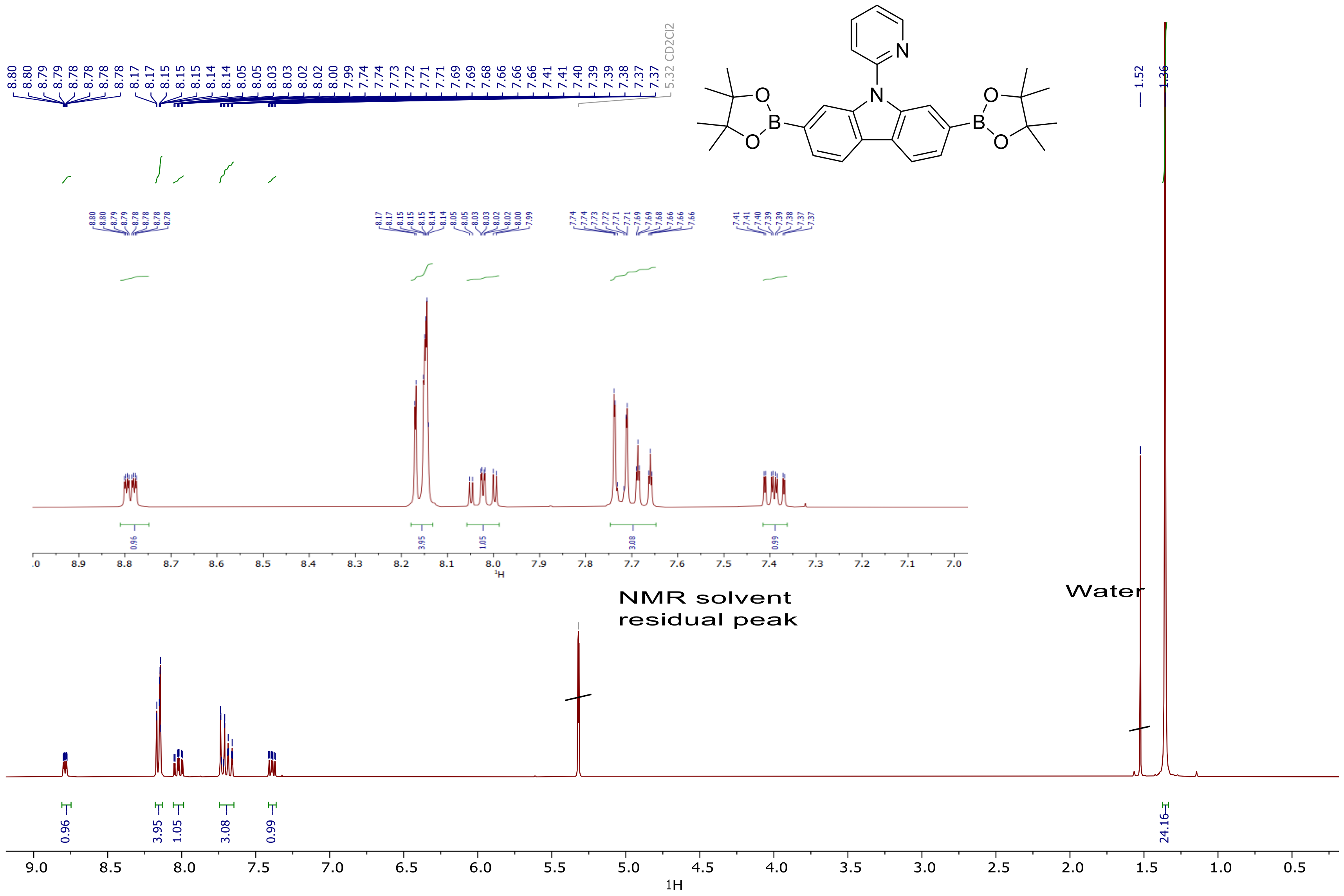

*Figure S 29 ^{1}H-NMR of **2b** in CD_2Cl_2*

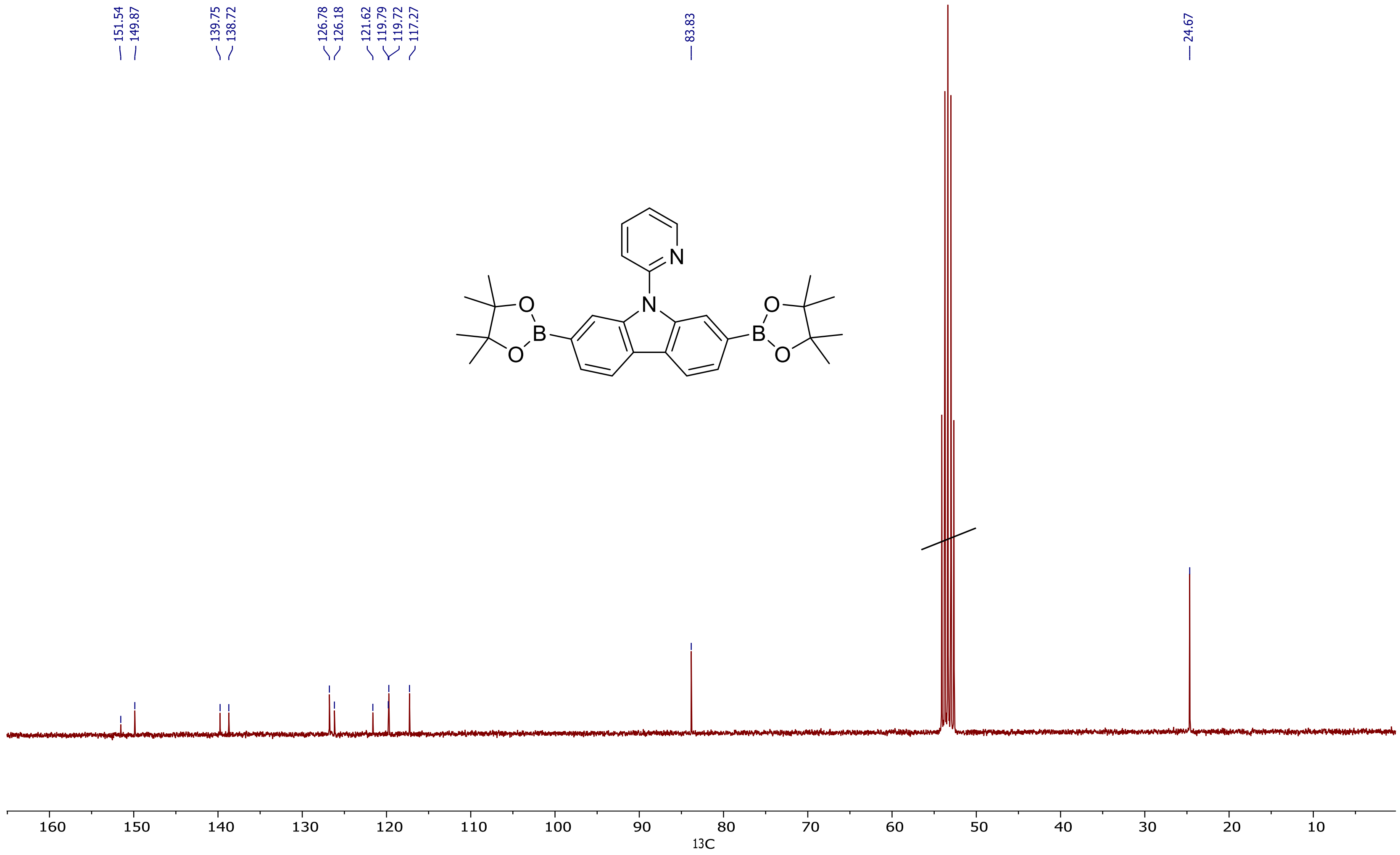

Figure S 30 ^{13}*C-NMR of* ***2b*** *in* CD_2Cl_2

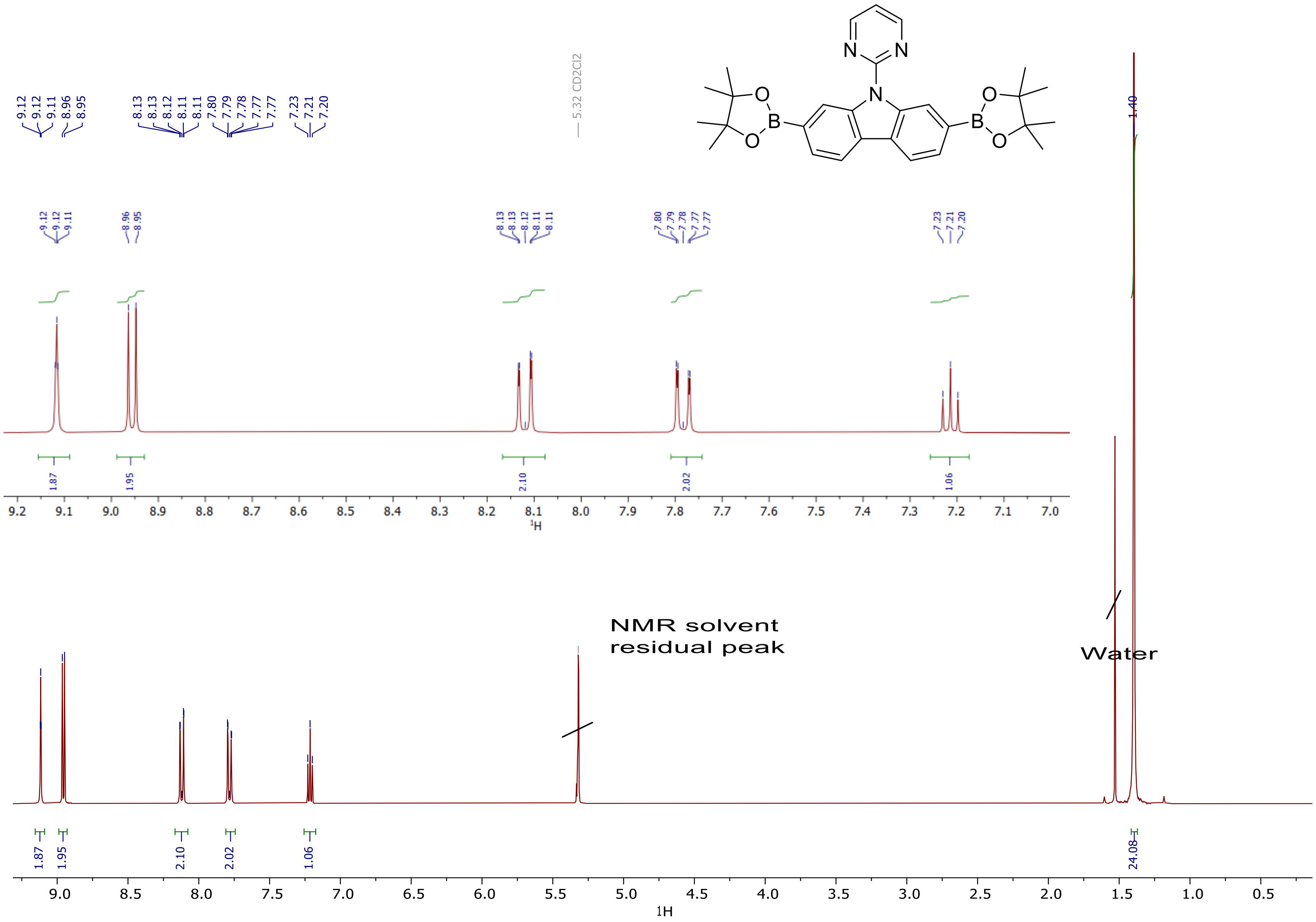

Figure S 31 ^{1}H-NMR of ***2c*** *in CD_2Cl_2*

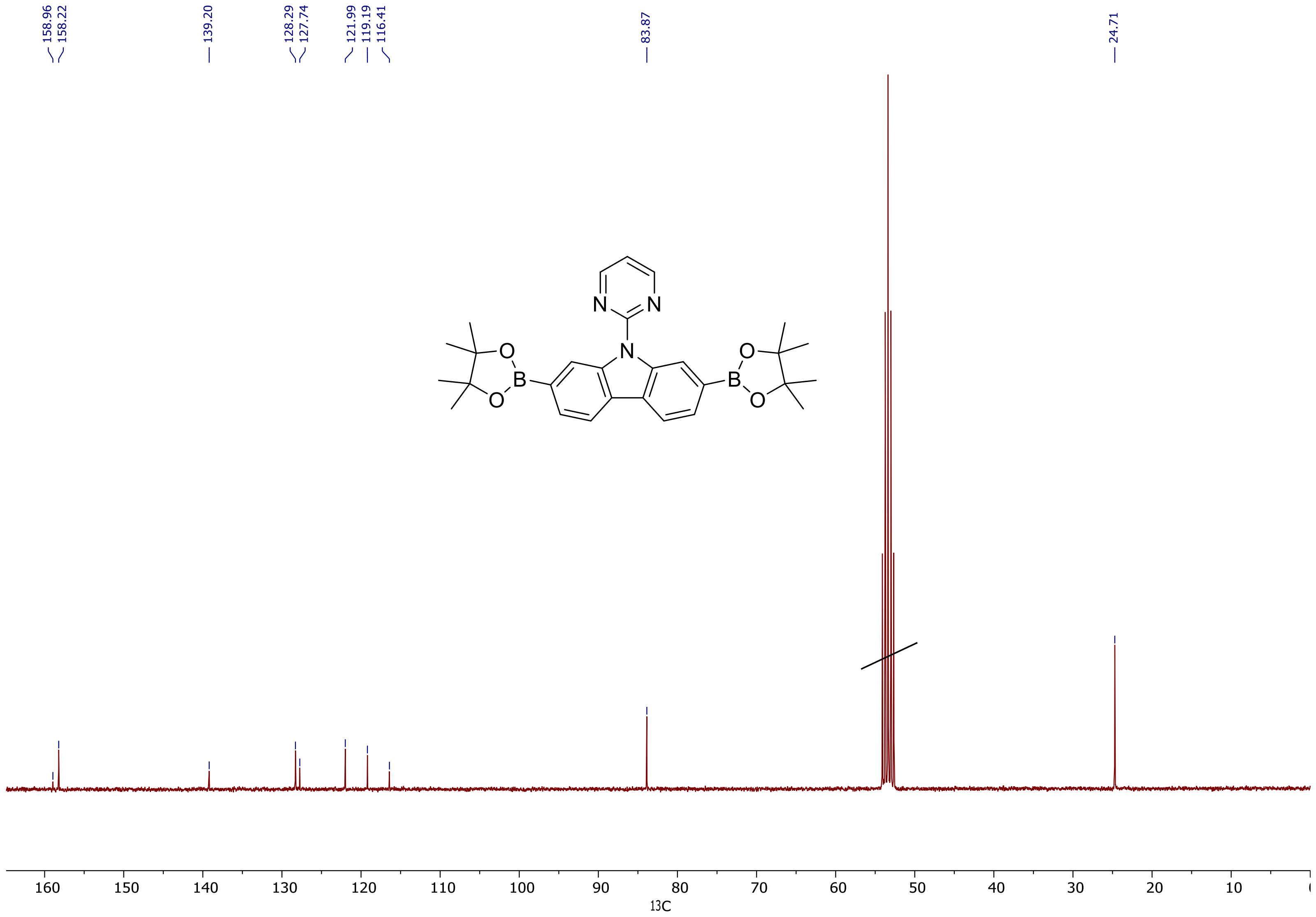

*Figure S 32 ^{13}C-NMR of **2c** in CD_2Cl_2*

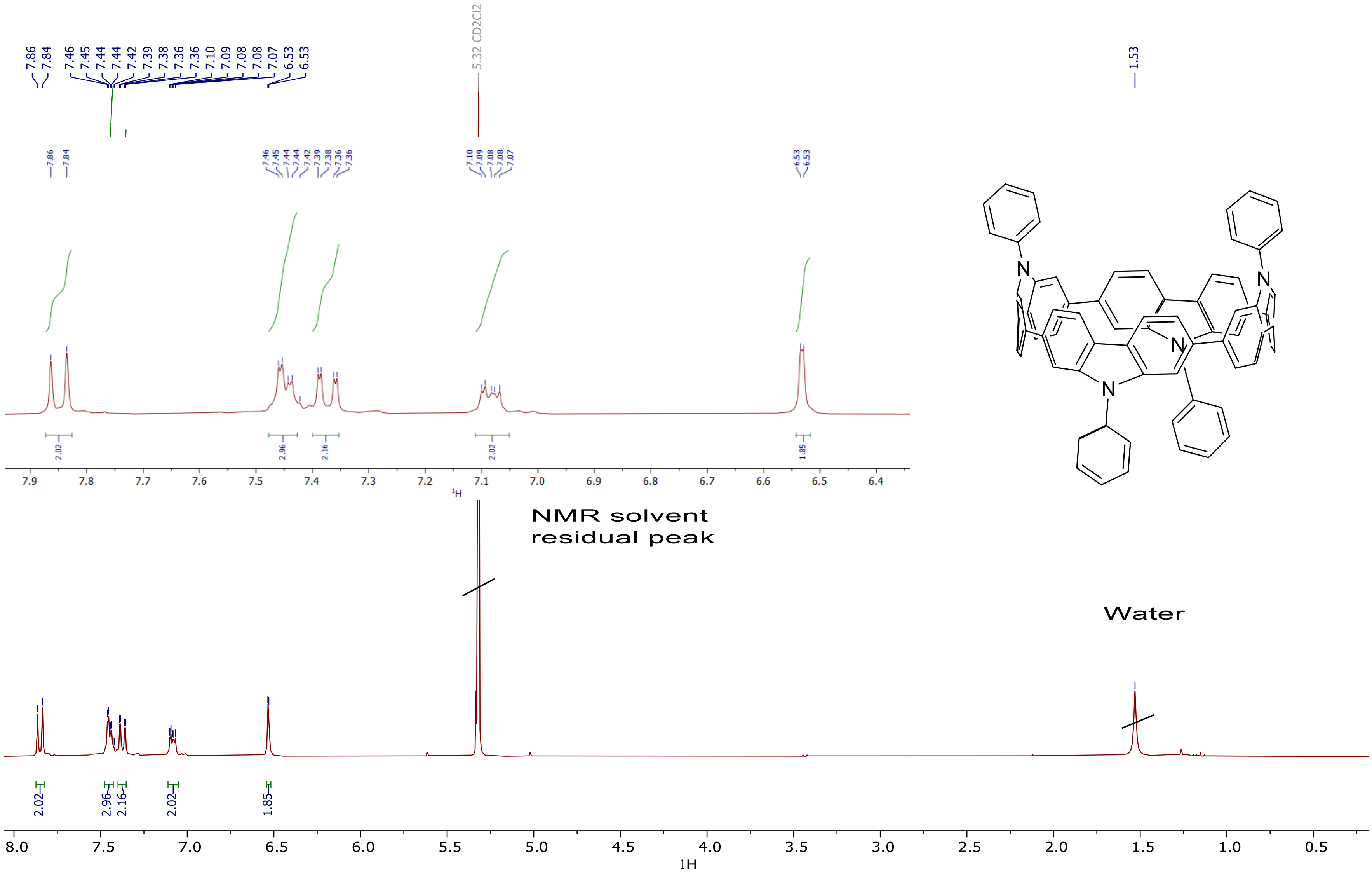

Figure S 33 [1]*H-NMR of* ***[4]C-Ph-Cbz*** *in* CD_2Cl_2

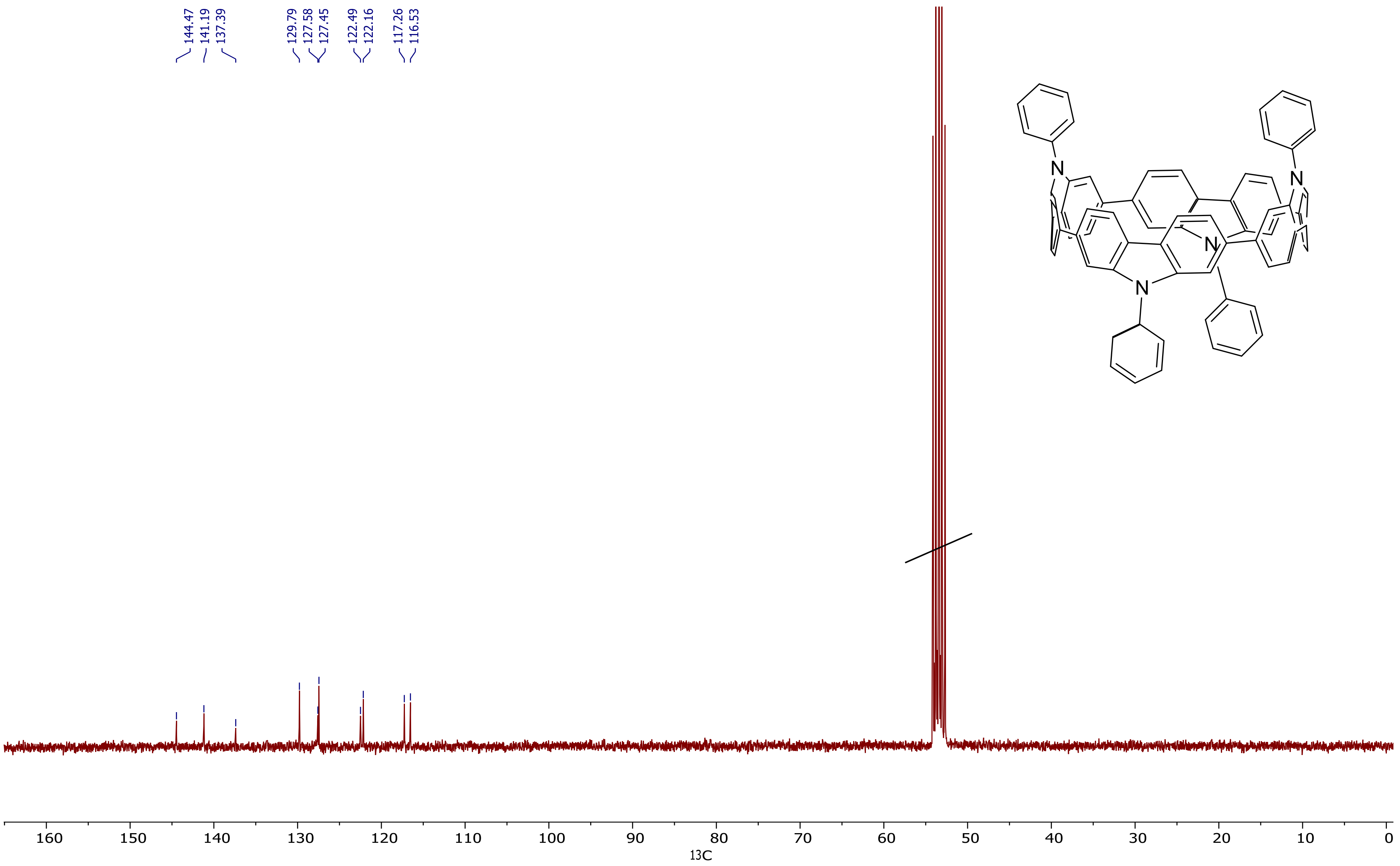

Figure S 34 ^{13}*C-NMR of* ***[4]C-Ph-Cbz*** *in* CD_2Cl_2

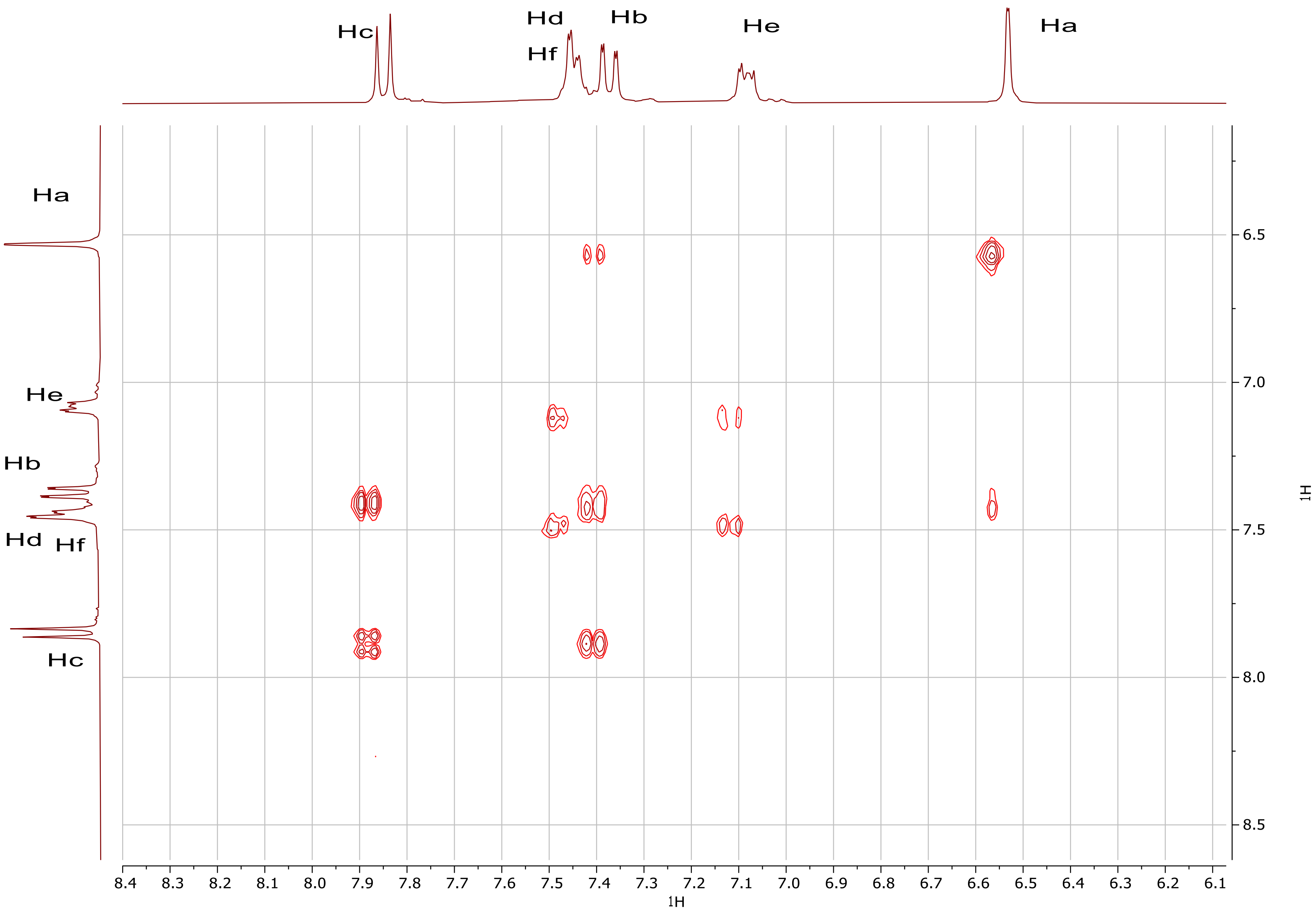

*Figure S 35 COSY NMR of **[4]C-Ph-Cbz** in CD_2Cl_2*

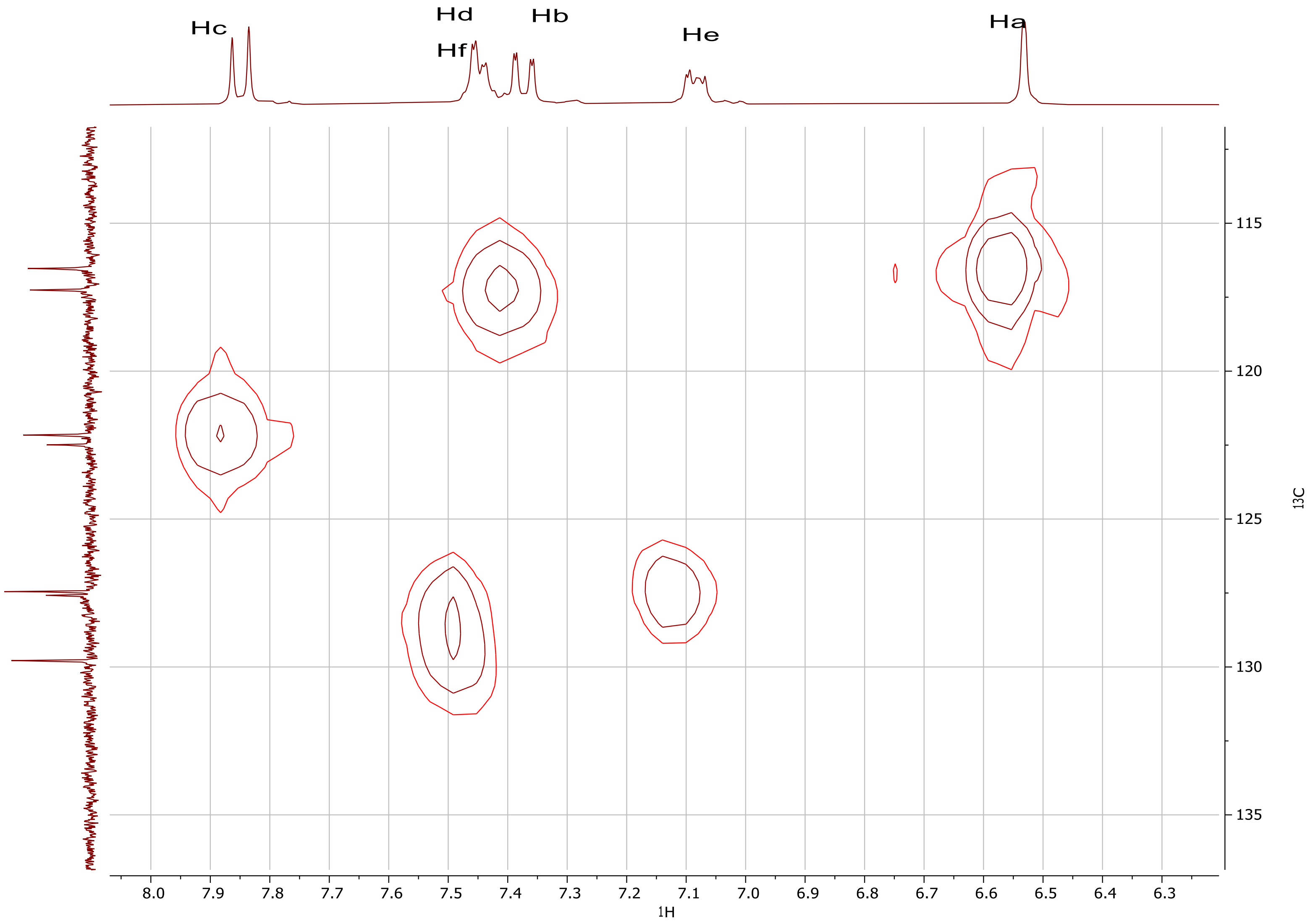

Figure S 36 HMQC NMR of ***[4]C-Ph-Cbz*** *in* CD_2Cl_2

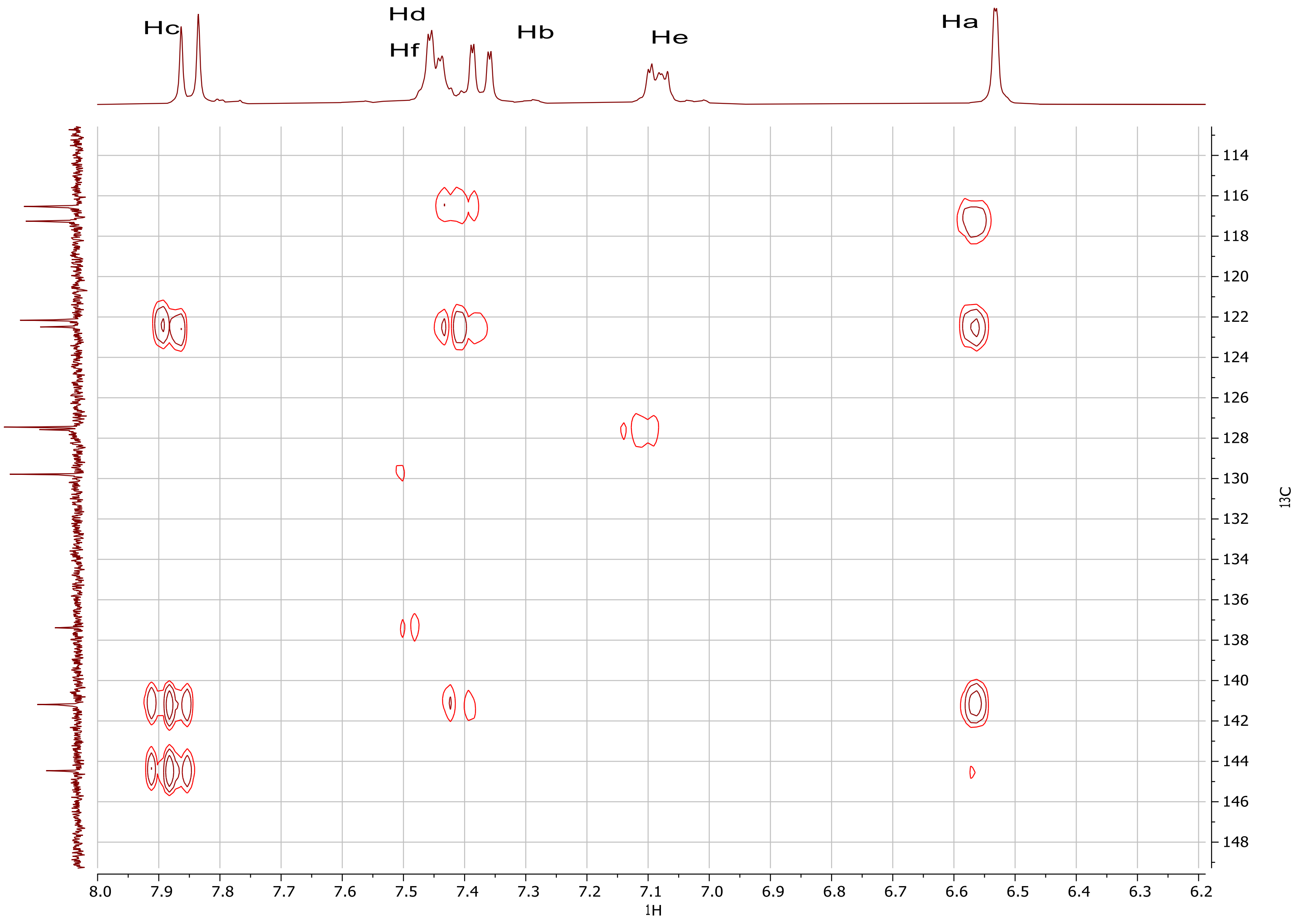

*Figure S 37 HMBC NMR of **[4]C-Ph-Cbz** in CD_2Cl_2*

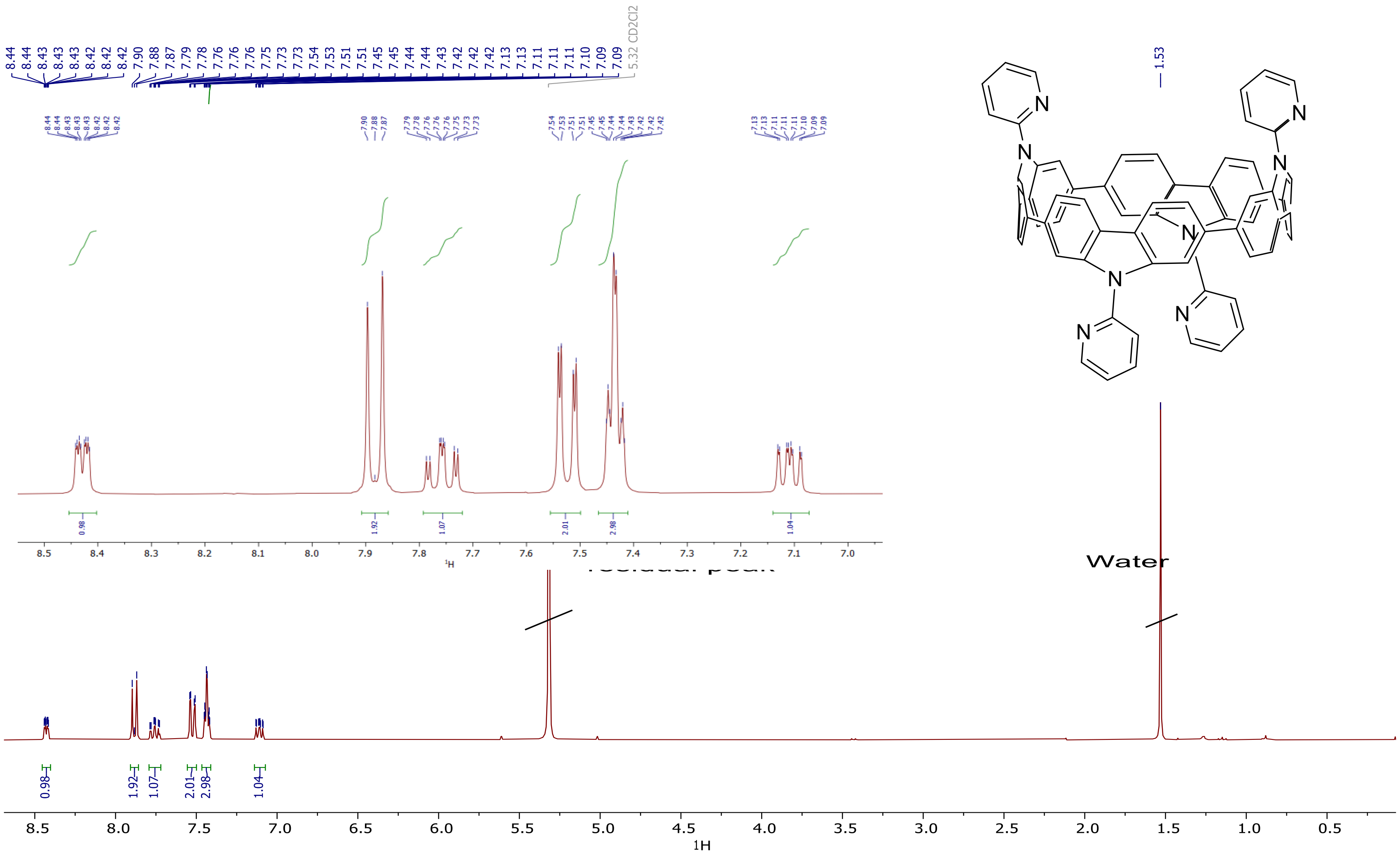

*Figure S 38 ^{1}H-NMR of **[4]C-Py-Cbz** in CD_2Cl_2*

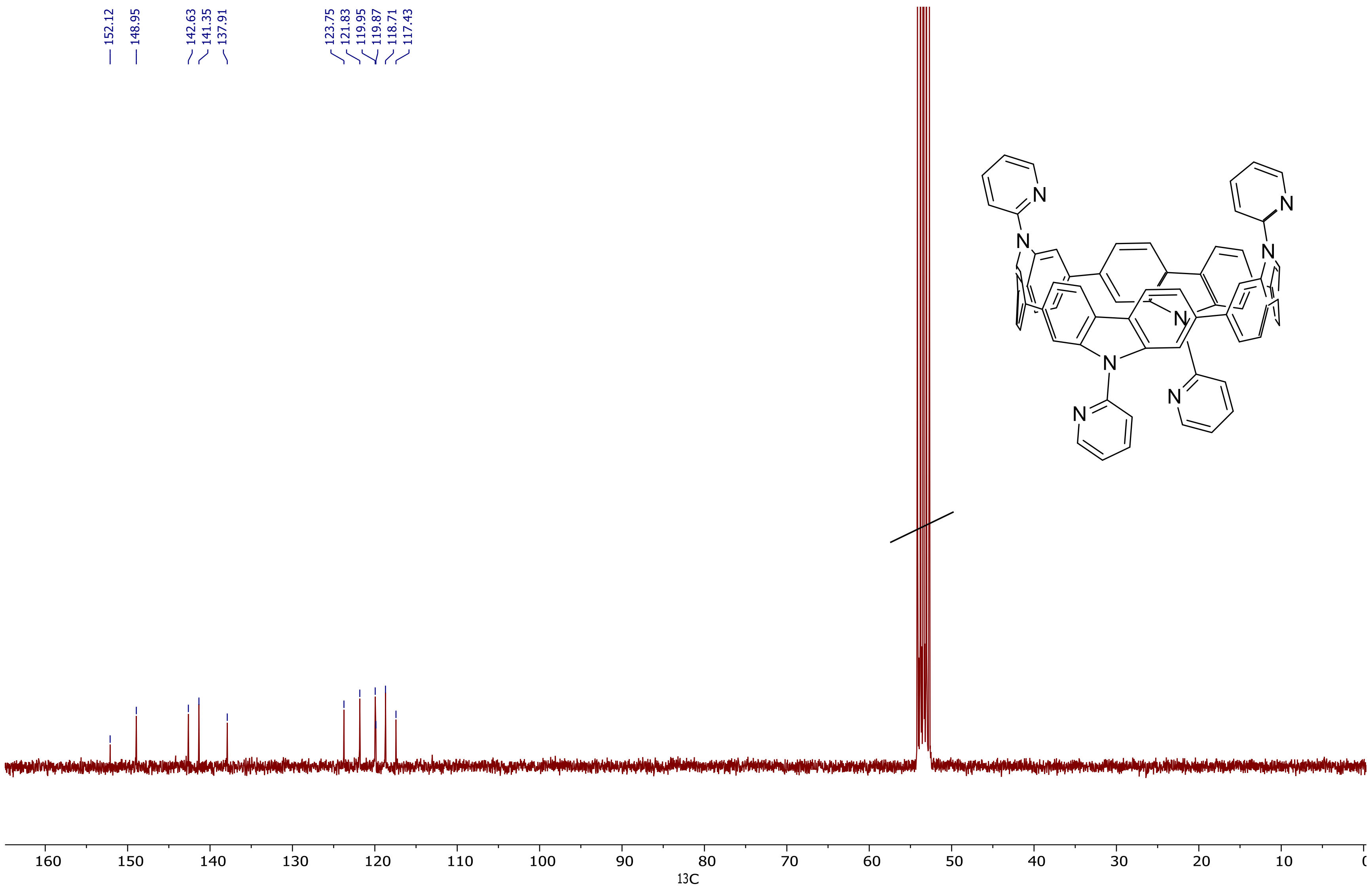

Figure S 39 ^{13}*C-NMR of* ***[4]C-Py-Cbz*** *in* CD_2Cl_2

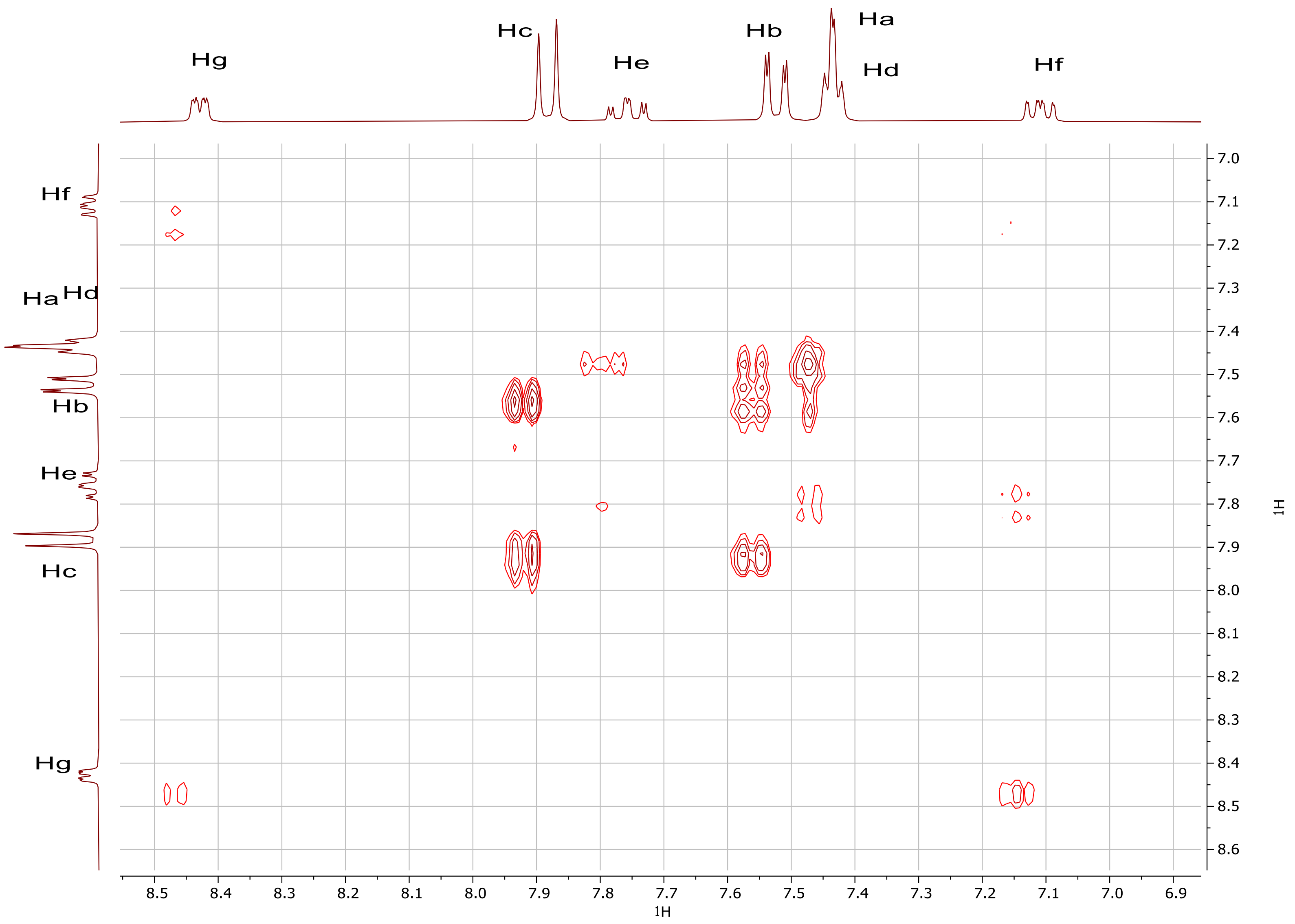

Figure S 40 COSY NMR of ***[4]C-Py-Cbz*** *in* CD_2Cl_2

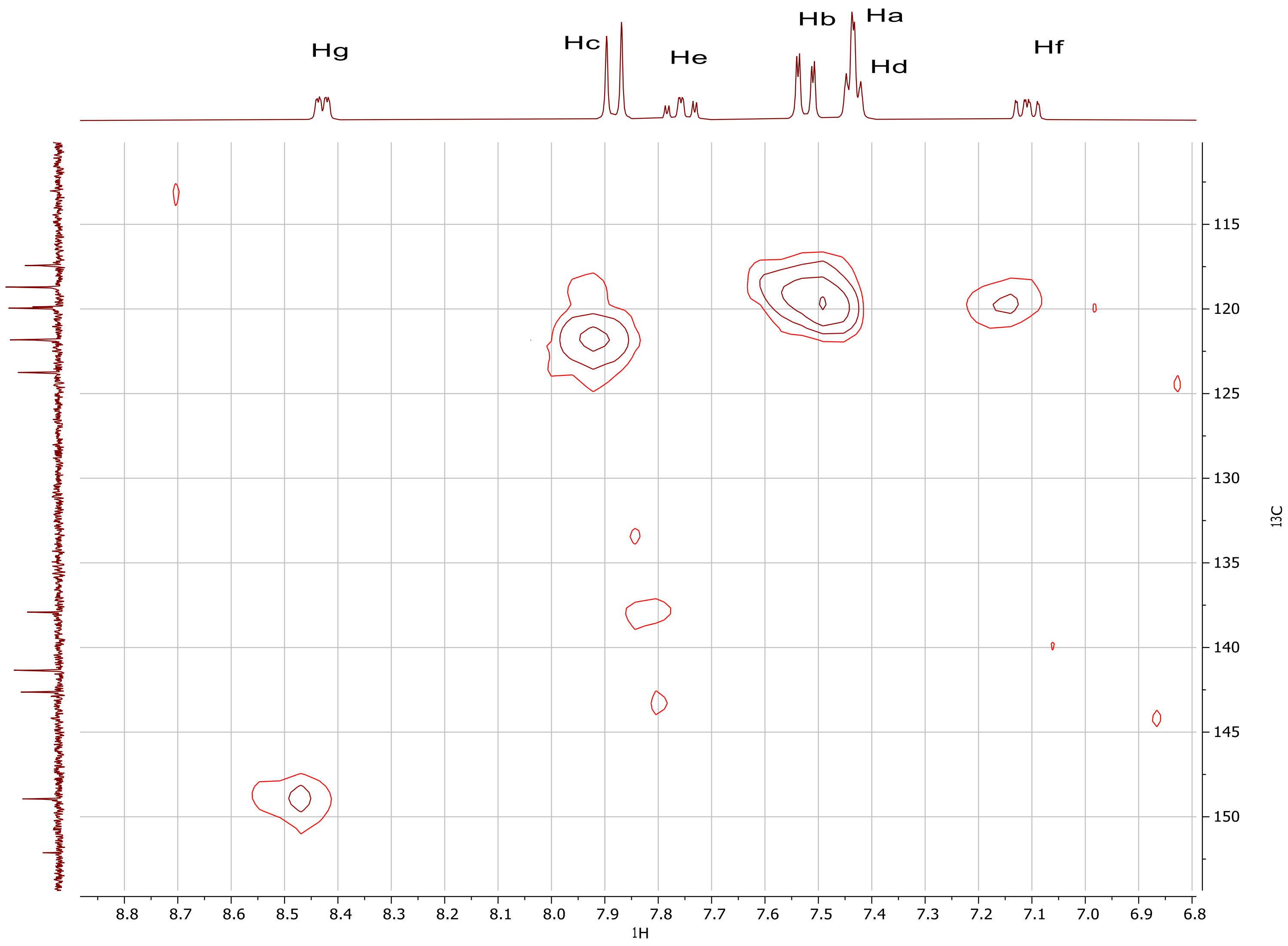

Figure S 41 HMQC NMR of ***[4]C-Py-Cbz*** *in* CD_2Cl_2

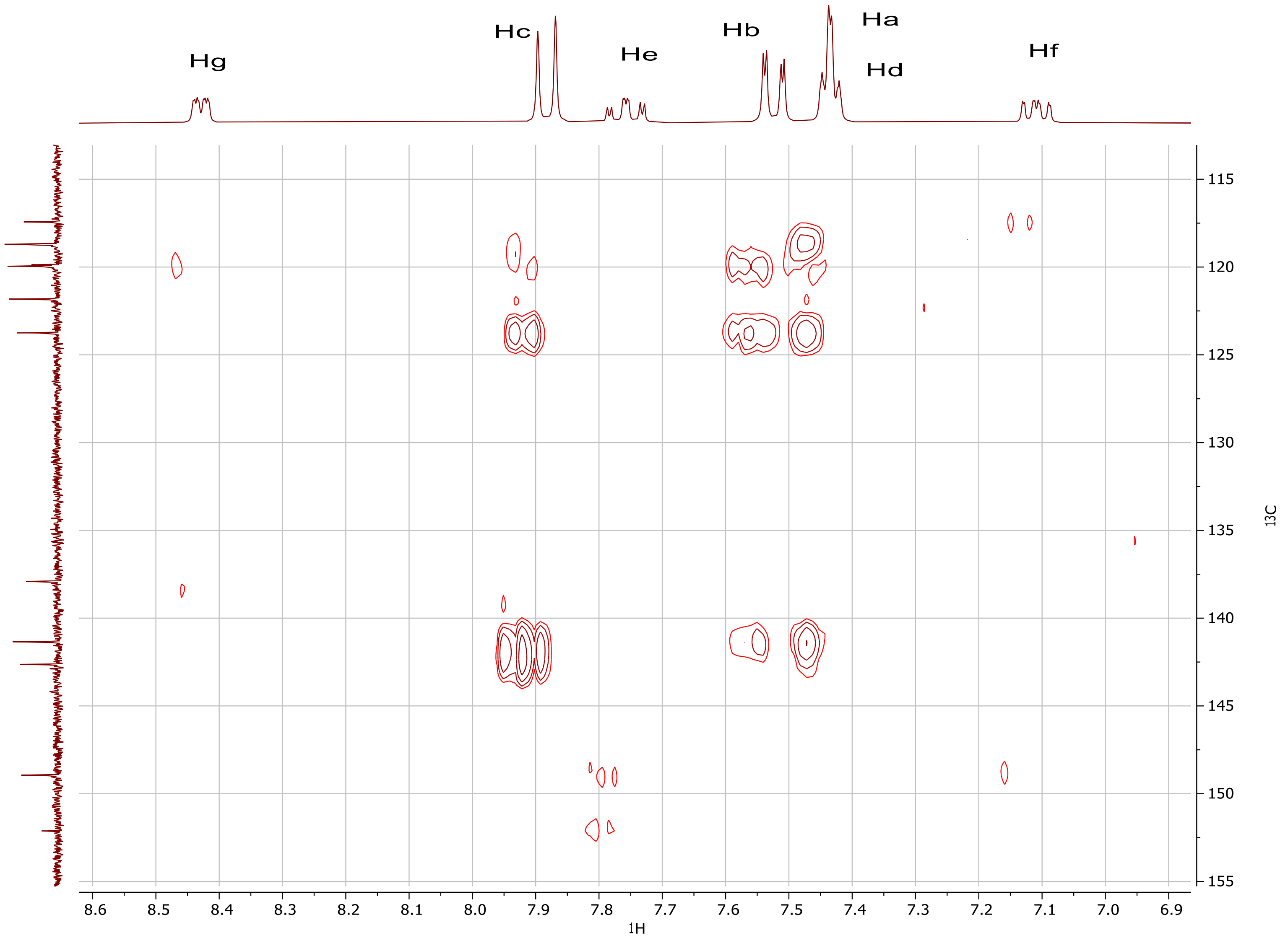

*Figure S 42 HMBC NMR of **[4]C-Py-Cbz** in CD_2Cl_2*

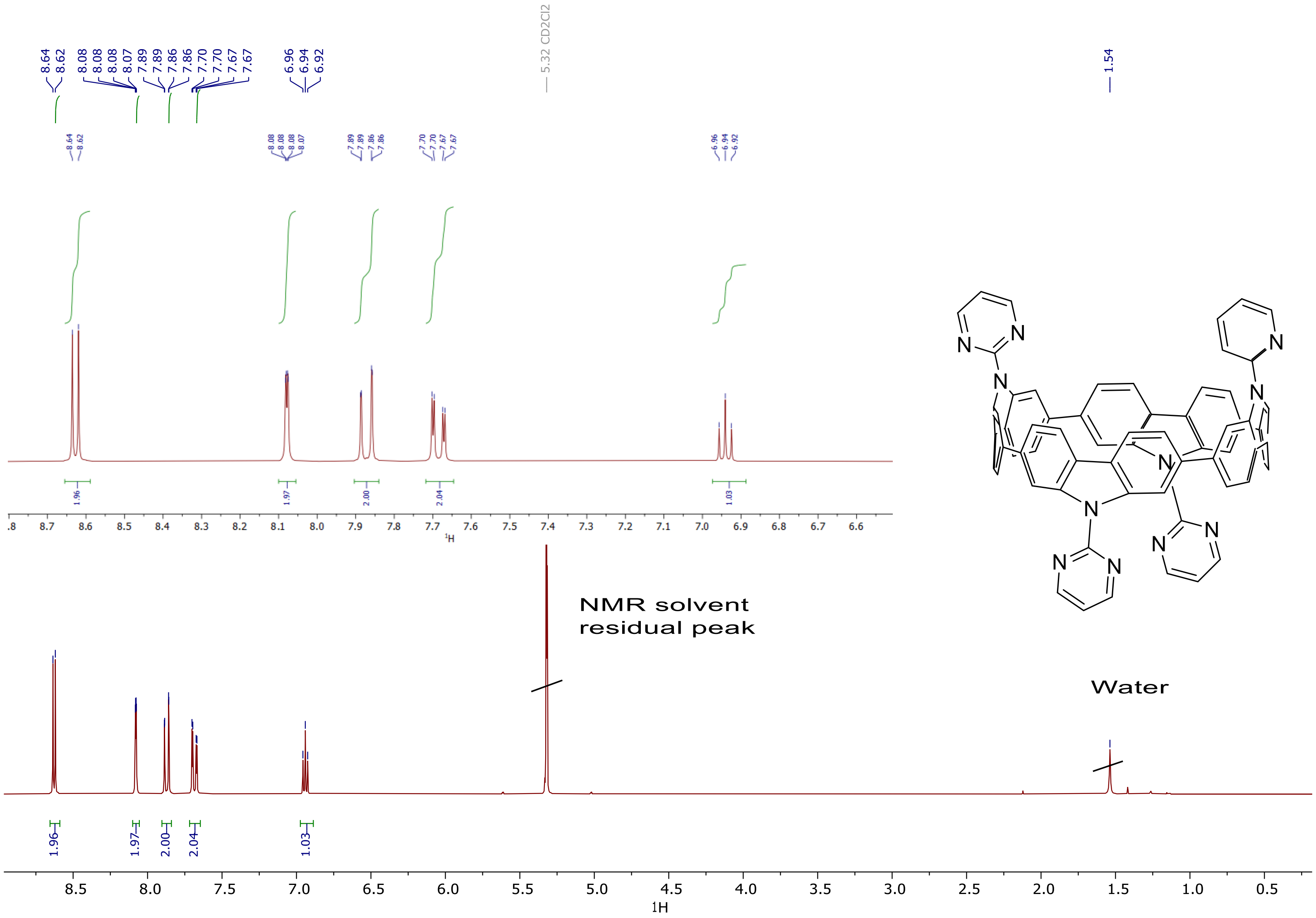

*Figure S 43 ^{1}H-NMR of **[4]C-Pm-Cbz** in CD_2Cl_2*

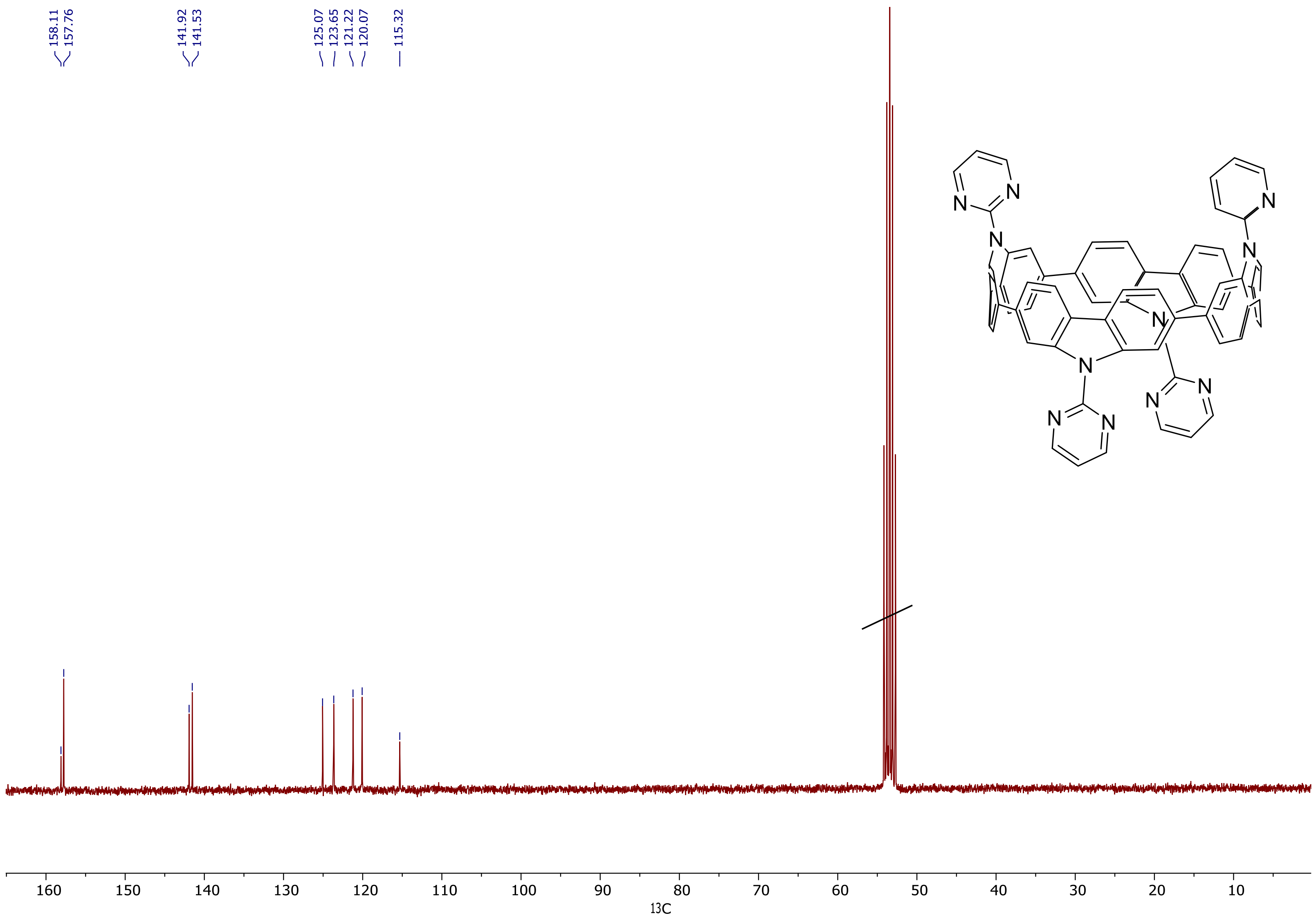

Figure S 44 ^{13}*C-NMR of* ***[4]C-Pm-Cbz*** *in* CD_2Cl_2

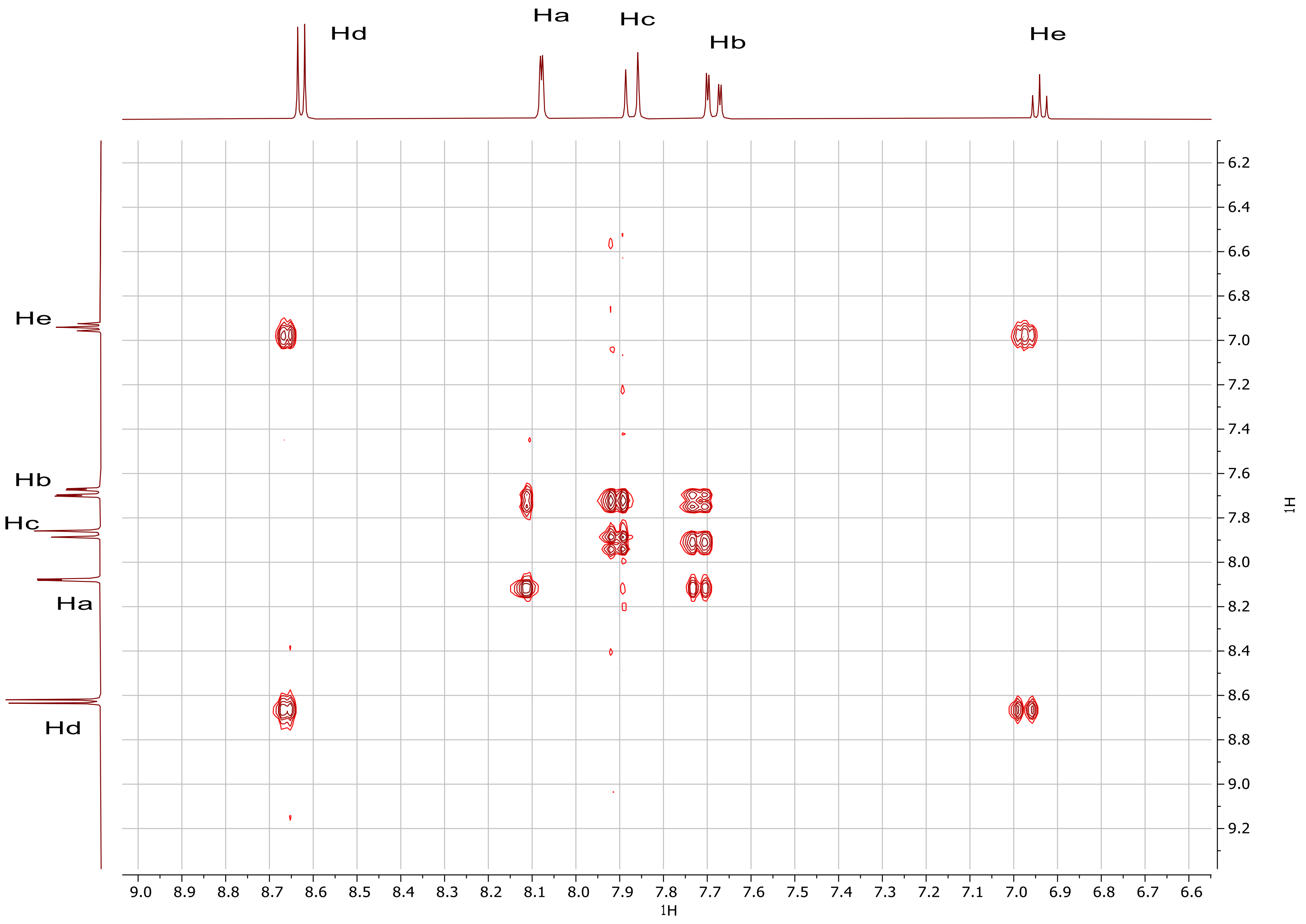

Figure S 45 COSY NMR of ***[4]C-Pm-Cbz*** *in* CD_2Cl_2

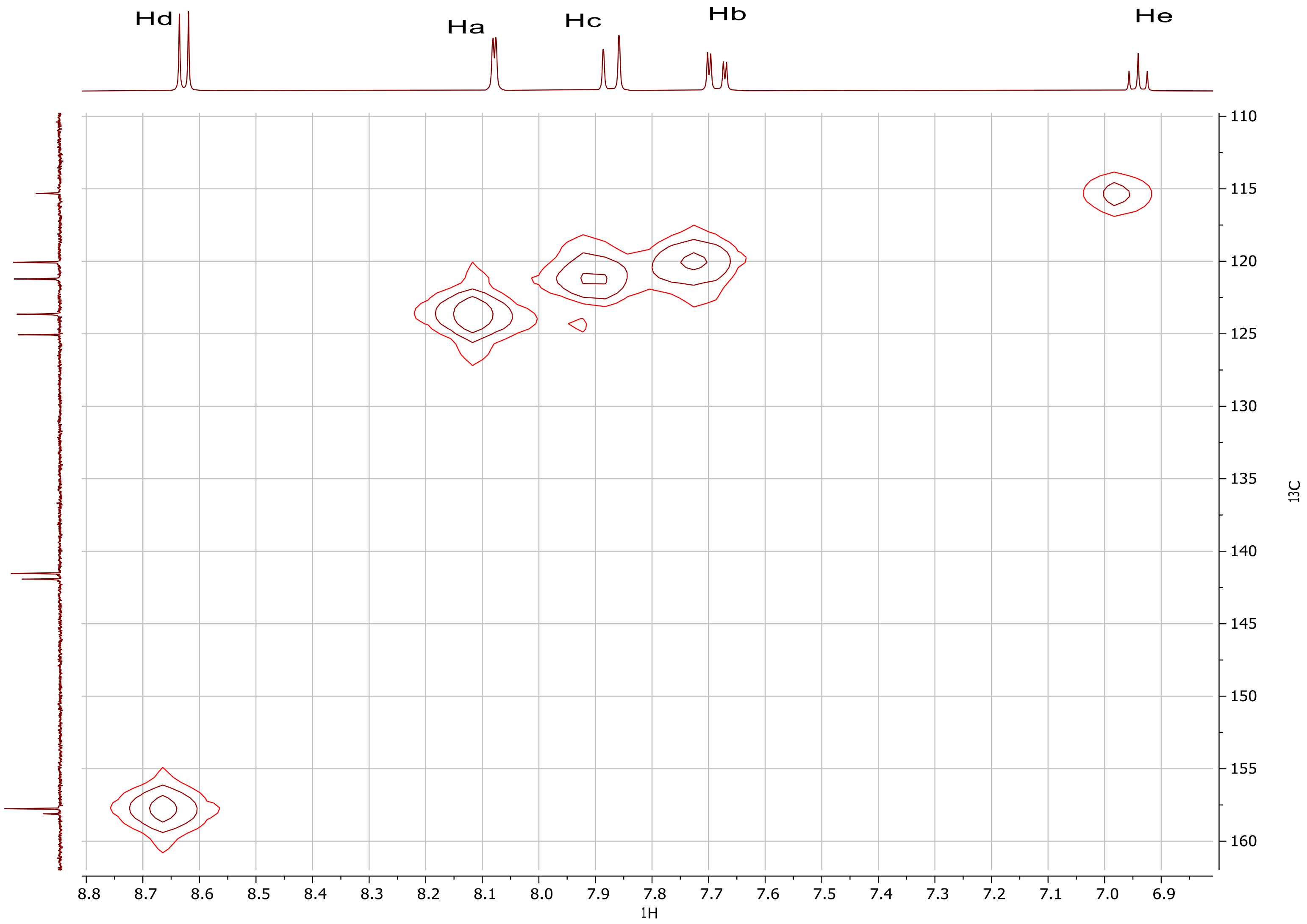

*Figure S 46 HMQC NMR of **[4]C-Pm-Cbz** in CD_2Cl_2*

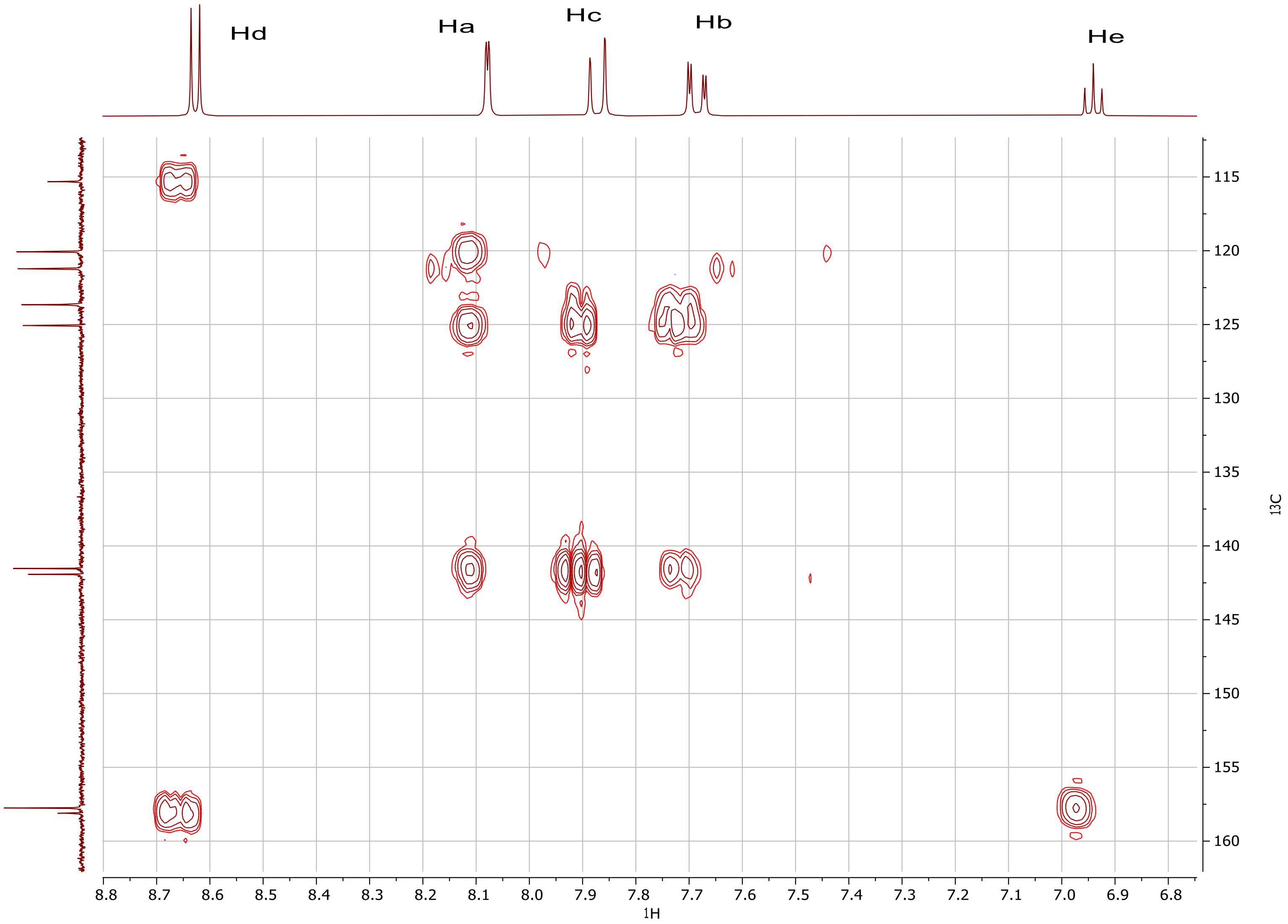

Figure S 47 HMBC NMR of ***[4]C-Pm-Cbz*** *in CD_2Cl_2*

13 References

1. Fulmer, G. R.; Miller, A. J. M.; Sherden, N. H.; Gottlieb, H. E.; Nudelman, A.; Stoltz, B. M.; Bercaw, J. E.; Goldberg, K. I., *Organometallics* **2010,** *29* (9), 2176.
2. Kulkarni, A. P.; Tonzola, C. J.; Babel, A.; Jenekhe, S. A., *Chem. Mater.* **2004,** *16* (23), 4556.
3. Iwamoto, T.; Watanabe, Y.; Sakamoto, Y.; Suzuki, T.; Yamago, S., *J. Am. Chem. Soc.* **2011,** *133* (21), 8354.
4. Jiang, W.; Duan, L.; Qiao, J.; Dong, G.; Zhang, D.; Wang, L.; Qiu, Y., *J. Mater. Chem.* **2011,** *21* (13), 4918.
